# The Careful Combination of Bismuth Compounds and CAAC: Formal Halonium and Nitrenium Transfer via Radical Pathways

**DOI:** 10.1002/chem.202500913

**Published:** 2025-04-17

**Authors:** Gargi Kundu, Ahmed Fetoh, Jordi Poater, Crispin Lichtenberg

**Affiliations:** ^1^ Fachbereich Chemie Philipps‐Universität Marburg Hans‐Meerwein‐Str. 4 Marburg 35037 Germany; ^2^ Department of Chemistry Faculty of Science Mansoura University El Gomhouria, Mansoura Qism 2, Dakahlia Governorate Mansoura 11432 Egypt; ^3^ Departament de Química Inorgànica i Orgànica & IQTCUB Universitat de Barcelona Barcelona 08028 Spain; ^4^ ICREA Barcelona 08010 Spain

**Keywords:** bismuth, carbenes, Lewis pairs, radicals, reactive intermediates

## Abstract

Cyclic (alkyl)(amino)carbenes (CAACs) are key compounds en route to a plethora of unprecedented low‐valent and radical complexes of transition metals and main group elements. For p‐block elements of the sixth period, however, low‐valent and radical complexes of CAACs are extremely rare, and even simple adducts with CAACs are difficult to access. Here we report the full characterization of the first adduct between a CAAC and a bismuth trihalide, [BiCl_3_(^Me2^CAAC)] (**1**), which has previously only been spectroscopically characterized. **1** and its in situ generated bromido analog form intensely colored solutions in solvent molecule (THF) and undergo formal halonium ion (i.e., X^+^) transfer from the Bi atom to the carbene carbon atom to give the complex cations [CAAC─X]^+^ (X = Cl, Br). Starting from bismuth amide complexes, formal nitrenium ion (i.e., [NR_2_]^+^) transfer reactions are observed to give [CAAC─NR_2_]^+^. EPR spectroscopic reaction monitoring indicates the viability of radical processes and suggests the involvement of unprecedented α‐aminyl radicals and mononuclear Bi^0^ species. Our investigations reveal easily accessible radical pathways via peerless molecular complexes, which proceed in the absence of external reducing agents, as a key factor on the way to exploit the full potential of carbene ligands in the chemistry of sixth row p‐block elements.

## Graphical abstract

Fundamental bismuth compounds such as BiCl_3_ and BiBr(NMe_2_)_2_ form simple, colorful adducts with cyclic (alkyl)(amino)carbenes. They undergo facile and selective formal X^+^ and (NMe_2_)^+^ transfer from bismuth to the carbene via peerless radical pathways.

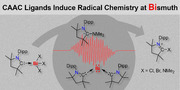

## 1 Introduction

Carbenes have been used extensively to form Lewis pairs and to stabilize low‐valent compounds, covering central atoms across major parts of the periodic table of the elements.^[^ ^1^, ^2^, ^3^, ^4^, ^5^ ^]^ When it comes to sixth row heavy p‐block elements, however, the triumphant march of carbene ligands seemed to come to an end, although a comparatively small number of carbene compounds relevant in this context could be isolated.^[^ ^1^ ^]^

In the case of bismuth, the heaviest p‐block element without relevant radioactivity, the entrance into carbene chemistry was made in 2014, when Dutton and co‐workers first reported adducts of the type NHC·BiCl_3_ (NHC = N‐heterocyclic carbene).^[^ ^6^ ^]^ In the same line, Goicoechea and co‐workers reported an NHC‐stabilized bismuth tribromide adduct through the treatment of IDipp with BiBr_3_, which readily isomerizes to give a bismuth adduct of the abnormal carbene.^[^ ^7^ ^]^ More recently, Tamm and co‐workers presented an NHC ligand with an anionic substituent in the backbone (WCA‐NHC), which could be exploited for the synthesis of [BiCl_2_(WCA‐NHC)] and its subsequent reduction to yield the corresponding dibismuthene.^[^ ^8^, ^9^ ^]^

**Scheme 1:**
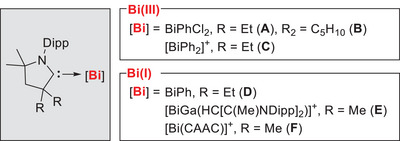
Structurally authenticated CAAC bismuth compounds. Dipp = 2,6‐*i*Pr_2_‐C_6_H_3_.

Within the large and ever‐growing number of carbene‐stabilized species, cyclic (alkyl)(amino)carbenes (CAACs) adopt a special role since they have facilitated the isolation of numerous compounds where NHCs have been inefficient, due to the stronger σ‐donating and π‐accepting nature of CAACs.^[^ ^3^, ^5^, ^10^, ^11^ ^]^ In 2018, Gilliard and co‐workers reported that their initial efforts to access low‐valent bismuth compounds starting from CAAC and BiCl_3_ were unsuccessful. This was attributed to the fact that even these simple mixtures of CAAC and BiCl_3_ were unstable, leading to the precipitation of elemental bismuth prior to reactions with other reagents.^[^ ^12^ ^]^ Schulz and co‐workers recently presented a protocol to access [BiCl_3_(^Me2^CAAC)], but the compound was only characterized by ^1^H and ^13^C NMR spectroscopy.^[^ ^13^, ^14^ ^]^ Taming the Lewis acidity of the bismuth component by exchanging one Cl atom for a Ph group,^[^ ^15^ ^]^ the first CAAC bismuth(III) complexes could be obtained and thoroughly characterized, CAAC·PhBiCl_2_ (**A** and **B**, Scheme 1).^[^ ^12^ ^]^ However, the direct reduction of **A** to afford bismuthinidine **D** was unsuccessful, and could only be achieved via reduction and ligand transfer by a Be(0) complex.^[^ ^16^ ^]^ The complexity of bismuth‐CAAC chemistry was further underlined by the fact that the reaction of ^Et2^CAAC with BiPh_2_Cl did not yield a simple adduct, but instead the CAAC‐stabilized cation [BiPh_2_^Et2^CAAC]^+^ (**C**, Scheme 1).^[^ ^17^ ^]^ Low‐valent compounds including the structurally authenticated cationic species **E** have been obtained by Schulz et al., exploiting a gallium‐based ligand system.^[^ ^13^, ^14^ ^]^ Recently, Roesky and co‐workers reported the CAAC‐stabilized Bi(I) compound **F** obtained by *in‐situ* reduction of ^Dipp^CAAC─LiOTf and BiCl_3_.^[^ ^18^ ^]^

Despite these recent advances, the questions remain: why is the predictable synthesis of Lewis‐acid‐base adducts between carbenes and compounds based on sixth row p‐block elements such as bismuth so challenging, leading to unforeseen reaction pathways, temperature‐sensitive compounds, and seemingly intractable degradation reactions?

Here we report the isolation and thorough characterization of Lewis‐acid‐base adducts between CAACs and Lewis acidic bismuth compounds and elucidate their redox‐governed decomposition pathways, which include formal halonium and nitrenium transfer to CAACs via radical intermediates.

## 2 Results and Discussion

**Scheme 2:**
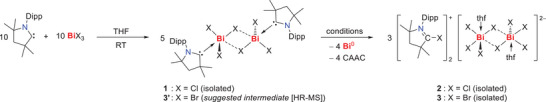
Reactivity of ^Me2^CAAC with bismuth halides (BiX_3_; X = Cl, Br), and isolation of **1**, **2,** and **3**. Conditions for X = Cl: 7 days, RT; for X = Br: 2 days, −30 °C.

Despite reports on its inherent instability^[^ ^7^ ^]^ and in view of recent advances in the field,^[^ ^13^, ^14^ ^]^ we wondered: is it possible to thoroughly characterize the structurally elusive parent CAAC─BiX_3_ complex? To explore this, we conducted the reaction of ^Me2^CAAC with one equivalent of BiCl_3_ in solvent molecule (THF), resulting in the immediate formation of an intensely green solution. After maintaining this solution at room temperature for 24 hours, the adduct ^Me2^CAAC─BiCl_3_ (**1**) could be isolated as a green crystalline solid (Scheme 2). ^Me2^CAAC─BiCl_3_ was thoroughly characterized using NMR spectroscopy, mass spectrometry, elemental analysis, and single‐crystal X‐ray diffraction studies.

The ^1^H NMR spectrum of **1** in CD_2_Cl_2_ revealed a characteristic singlet at 2.24 ppm for the CH_2_ protons of the five‐membered heterocycle, which is shifted downfield compared to 1.54 ppm for the free ^Me2^CAAC. The ^15^N NMR chemical shift for compound **1** at −137.3 ppm indicates that the CAAC ligand acts as strong σ‐donor towards BiCl_3_ with negligible Bi→CAAC back donation.^[^ ^19^ ^]^ This is in agreement with the relativistic contraction of the s(Bi) atomic orbital, which tends to enable Bi→acceptor interactions preferably towards sufficiently strong and soft Lewis bases such as GaR_3_, [BiR_2_]^+^, and Pt^2+^.^[^ ^20^, ^21^, ^22^ ^]^

**Figure 1:**
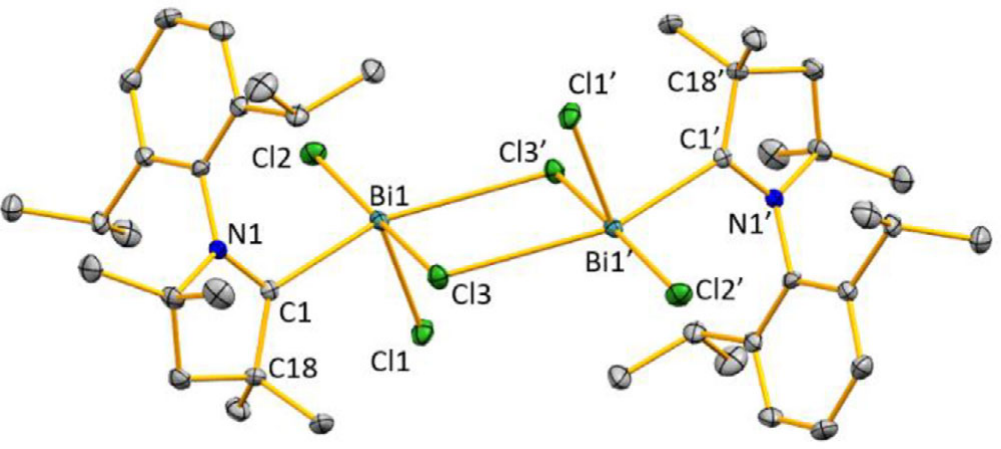
The molecular structure of **1** in the solid state. Hydrogen atoms are omitted for clarity. Selected bond lengths (Å) and angles (°): N1─C1 1.295(3), C1─C18 1.513(3), Bi1─C1 2.394(2), Bi1─Cl1 2.4662(5), Bi1─Cl2 2.5951(5), Bi1─Cl3 2.7621(5), Bi1─Cl3’ 3.1302(5); N1─C1─Bi1 116.48(14), N1─C1─C18 111.04(18), Cl1─Bi1─Cl2 92.501(18), Cl1─Bi1─Cl3 95.200(18), Cl2─Bi1─Cl3 172.096(17).

While ^R2^CAAC─PhBiCl_2_ (R_2_ = Et_2_, C_5_H_10_) have been reported to decompose to metallic bismuth and free CAAC in THF solutions above −20 °C, ^Me2^CAAC─BiCl_3_ (**1**) remains stable at 20 °C in THF solution for 24 hours according to NMR spectroscopy was isolated in 60% yield. In the solid state, **1** is stabilized in a dimeric form, featuring a Bi_2_Cl_2_ core formed by the *μ*_2_‐bridging chlorine atoms (Figure 1). The mononuclear subunits are symmetry‐related by a crystallographic inversion center, so bonding parameters of one subunit are discussed. The bismuth atoms adopt a distorted square pyramidal coordination geometry with Cl1 in the apical position. The Bi1–C1 distance in **1** amounts to 2.394(2) Å, which is slightly shorter than those reported for the CAAC‐Bi(III) adducts **A** (Bi─C^CAAC^: 2.4566(15) Å) and **B** (Bi─C^CAAC^: 2.4123(19) Å), reflecting the higher Lewis acidity of the BiCl_3_ complex fragment (compared to BiPhCl_2_).^[^ ^12^ ^]^ The ^Me2^CAAC ligand in **1** is located in the equatorial plane of the square pyramid, labilizing the chlorido ligand in *trans*‐position due to its excellent σ‐donor abilities. All three types of Bi─Cl bonds show different bond lengths, where the Bi1─Cl3 bond with a bridging Cl ligand is longer (2.7621(5) Å) than the Bi1─Cl2 bond with Cl2 in the equatorial plane (Bi1─Cl2 2.5951(5) Å) and Bi1─Cl1 with Cl1 in the apical position (Bi1─Cl1 2.4662(5) Å). The Bi1─Cl3′ contacts between mononuclear subunits (3.1302(5) Å) are similar to those in NHC·BiCl_3_ (3.129(2) Å).^[^ ^6^ ^]^

After keeping **1** at room temperature for more than 7 days, colorless needle‐shaped crystals were observed, along with a dark precipitate, presumably elemental bismuth. The colorless crystalline material was isolated in 65% yield and identified as ([^Me2^CAAC─Cl]^+^)_2_[Bi_2_Cl_8_(ligand [thf])_2_]^2−^ (**2**) (Scheme 2). Its formation can formally be rationalized by the transfer of a chloronium ion (Cl^+^) to the carbene carbon atom, inducing the reduction of Bi(III) according to the idealized reaction equation 5 ^[^ **^1^** ^]^ + 6 thf → 3 ^[^ **^2^** ^]^ + 4 ^Me2^CAAC + 4 Bi^0^. The ^1^H NMR spectrum of **2** in CD_2_Cl_2_ revealed a characteristic singlet at 2.99 ppm for the CH_2_ protons of the five‐membered heterocycle, compared to 2.28 ppm for **1**. The molecular ion peak for the cationic part at m/z = 320.21205 further confirms the formation of **2**. A closely related phenomenon has recently been reported by Radius and co‐workers in reactions of ^Me2^CAAC with SbMesCl_2_.^[^ ^23^ ^]^ While the adduct was not isolable, the ion pair [^Me2^CAAC─SbClMes] [SbCl_3_Mes] was formed initially and subsequently reacted with ^Me2^CAAC in a putative Cl^+^ transfer reaction to give [^Me2^CAAC─Cl] [SbCl_3_Mes] and [^Me2^CAAC─SbMes]. So, despite the absence of a stabilizing bulky organic ligand in compound **1**, the isolation of this simple Lewis acid/base pair could be achieved, strongly suggesting that the formal Cl^+^ ion transfer can proceed intramolecularly, rather than through the attack of an external ^Me2^CAAC ligand into a σ*(Cl─Bi) orbital. The mechanism involving the elimination of [^Me2^CAAC─Cl]^+^ from **1** could be regarded as a reductive elimination at the bismuth center. These observations inspired us to explore the reactivity of CAAC with other bismuth halides (BiX_3_) and halo amides (BiX_n_(NMe_2_)_3−n_), keeping in mind that the previously reported complex CAAC·BiPhCl_2_ did not show halide or R group transfer.^[^ ^12^ ^]^

The addition of ^Me2^CAAC to a solution of one equivalent of BiBr_3_ in THF at room temperature resulted in an instantaneous color change from pale yellow to dark green, suggesting the formation of the adduct [^Me2^CAAC·BiBr_3_]_2_ (**3′**). While **3′** could not be isolated to date, its intermediate formation is supported by high‐resolution mass spectrometric (HR‐MS) analyses, which yielded the molecular ion peak at *m/z* 654.0624 corresponding to [^Me2^CAAC─BiBr_2_]^+^. After keeping the reaction mixture at −30 °C for 2 days, colorless crystals of ([^Me2^CAAC─Br]^+^)_2_[Bi_2_Br_8_(thf)_2_]^2−^ (**3**) were obtained via formal Br^+^ ion transfer from bismuth to the carbene carbon center (Scheme 2). Compound **3** was isolated in 66% yield and characterized by NMR spectroscopy, mass spectrometry, elemental analysis, and single crystal X‐ray diffraction. The highest molecular ion peak at *m/z* 364.1634 in the HR‐MS spectrum confirms the presence of the ^Me2^CAAC─Br^+^ moiety. In the ^1^H NMR spectrum, the peak for the CH_2_ protons of the five‐membered heterocycle appears at 3.04 ppm, which is slightly down‐field shifted compared to the chlorine analogue. Both compounds **2** and **3** do not show significant halide→CAAC back donation, as indicated by ^15^N NMR chemical shifts of −148.3 and −142.2 ppm, respectively.^[^ ^19^ ^]^

**Figure 2:**
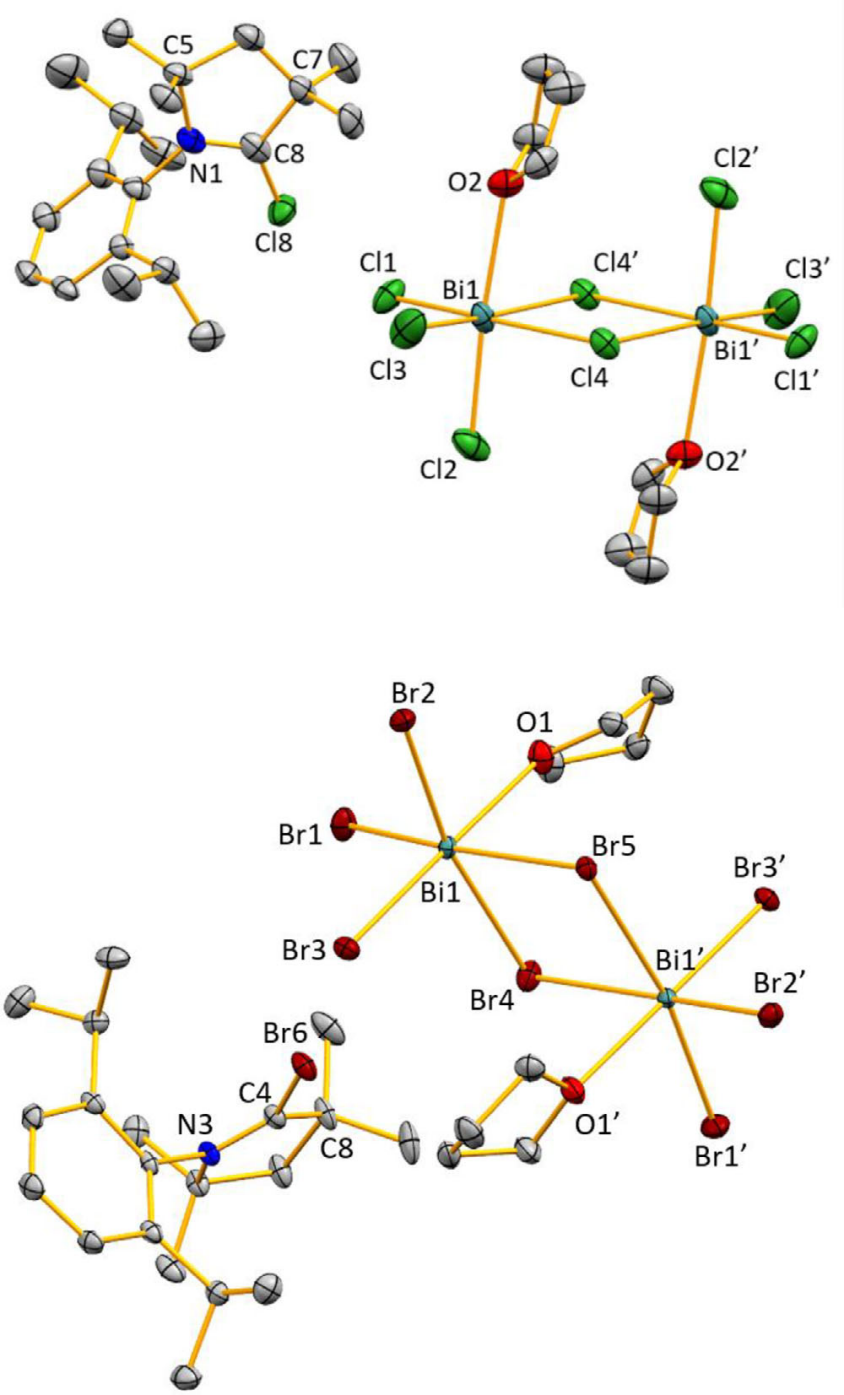
The molecular structures of **2** (top) and **3** (bottom). Hydrogen atoms are omitted for clarity. Selected bond distances (Å) and bond angles (deg): for **2**: N1─C8 1.280(8), C8─Cl1 1.518(7), C5─C6 1.540(9), Bi1─Cl1 2.562(2), Bi1─Cl2 2.549(2), Bi1‐Cl3 2.538(2), Bi1‐Cl4 2.888(2), Bi1─Cl4’ 2.916(2), Bi1─O2 2.599(5); N1─C8─C7 114.4(6), N1─C8─Cl6 122.9(5), C7─C8─Cl6 122.7(5), Cl1─Bi1─Cl4 173.57(5), Cl1─Bi1─Cl4’ 92.61(6), Cl3─Bi1─Cl4’ 92.57(6), Cl3─Bi1─Cl4 168.18(5), Cl2─Bi1–O2 176.25(11), Bi1─Cl4─Bi1’ 98.56(5); **3**: C4─N3 1.282(3), C4─C8 1.502(3), C4─Br6 1.851(2), Bi1─Br1 2.6966(3), Bi1─Br2 2.7301(3), Bi1─Br3 2.7571(3), Bi1─Br4 3.0191(3), Bi1─Br5 3.0522(3), Bi1─O1 2.5455(19); N3─C4─C8 115.4(2), N3─C4─Br6 123.03(18), C8─C4─Br6 121.45(17), Br1─Bi1─Br3 92.470(9), Br2─Bi─1Br3 92.991(8), Br1─Bi1─Br4 90.872(10), Br3─Bi1─Br4 95.018(7), Br1─Bi1─Br5 170.406(7), O1─Bi1─Br1 86.84(5), O1─Bi1─Br2 88.53(5), O1─Bi1─Br3 178.40(5).The molecular structure of **1** in the solid state. Hydrogen atoms are omitted for clarity. Selected bond lengths (Å) and angles (°): N1─C1 1.295(3), C1─C18 1.513(3), Bi1─C1 2.394(2), Bi1─Cl1 2.4662(5), Bi1─Cl2 2.5951(5), Bi1─Cl3 2.7621(5), Bi1─Cl3’ 3.1302(5); N1─C1─Bi1 116.48(14), N1─C1─C18 111.04(18), Cl1─Bi1─Cl2 92.501(18), Cl1─Bi1─Cl3 95.200(18), Cl2─Bi1─Cl3 172.096(17).

Compounds **2** and **3** crystallized in the triclinic space group *P* $\bar{1}$ and the monoclinic space group *C*2/*c*, respectively (Figure 2). The bismuth atoms in **2** and **3** are six‐coordinate. The O─Bi─Hal axes are close to linear (**2**: Cl2─Bi1–O2 176.25(11)°, **3**: O1─Bi1─Br3, 178.40(5)°). The equatorial Hal─Bi─Hal angles range between 81.43(5)°and 98.09(7)° in compound **2** and between 83.92(9)° and 96.32(10)° in **3**. Thus, the dianions [Bi_2_X_8_(thf)_2_]^2−^ (X = Cl, Br) adopt a slightly distorted octahedral coordination geometry. One halide atom and one thf ligand are located in the axial positions and the four remaining halide atoms are found in the equatorial plane with only minor deviations from the ideally expected bond angles of 180°(O─Bi─X) and 90° (X─Bi─X), respectively. The average Bi─Hal terminal distances are (**2**: 2.549 Å; **3**: 2.728 Å) significantly shorter than the bridging Bi─Hal distances (**2**: 2.902 Å; **3**: 3.036 Å). The dative type Bi─O bonds (**2**: 2.599(5) Å; **3**: 2.5455(19) Å) are shorter than that in SIDipp─BiPhCl_2_(thf) (2.814(2) Å),^[^ ^12^ ^]^ but in the range reported for Bi─O^thf^ bond lengths (2.404(7)−3.016(9) Å).^[^ ^24^, ^25^ ^]^

Room temperature UV/vis absorption spectroscopy of a green solution of compound **1** in THF shows absorption maxima at 324 and 457 nm (Figures S24). Density functional theory (DFT) calculations including a concentration correction indicate that the dissociation of **1** to give mononuclear [BiCl_3_(CAAC)(thf)] is possible (Δ*G* = −3.1 kcal·mol^−1^, Table S15). Time‐dependent density functional theory (TD‐DFT) calculations further support the presence of the mononuclear compound under conditions of the UV/vis spectroscopic experiment and assign the band in the visible region to a HOMO→LUMO transition (Figure S30). While the HOMO shows significant contributions by the 6s(Bi) and the 3p(Cl) atomic orbitals as well as the sp^2^(C^CAAC^) hybrid orbital, the LUMO largely reflects an antibonding π*(C─N) orbital. Qualitatively identical results were obtained for the bromo analogue **3′** (Figure S26), but the data are not discussed in detail due to uncertainties linked to the lower life‐time of the compound at ambient temperature (Supporting Information).

We were wondering, if the halonium transfer from bismuth to the CAAC entity could be extended to other functional groups. Since this reaction is coupled to an electron transfer to the pnictogen atom (to give low‐valent byproducts; cf. Scheme 2), bismuth amides appeared to be a promising choice. This class of compounds has not only enabled unusual electronic structures,^[^ ^26^ ^]^ intriguing insertion reactions,^[^ ^27^, ^28^, ^29^, ^30^, ^31^ ^]^ C─H activation,^[^ ^32^, ^33^, ^34^, ^35^, ^36^ ^]^ small molecule activation,^[^ ^27^, ^37^, ^38^, ^39^ ^]^ and polymerization catalysis,^[^ ^40^ ^]^ but also allowed for radical reactions with selective NR_2_ group transfer and the reduction of the Bi(III) center of the starting material: 2 Bi(NR_2_)_3_ → 3 R_2_N─NR_2_ + 2 Bi^0^.^[^ ^36^, ^41^ ^]^

Specifically, we aimed at the little‐investigated class of halobismuthamides [Bi(NR_2_)_3−n_Hal_n_] to facilitate the coordination of the CAAC ligand by C^CAAC^→σ*(Bi─Hal) interactions in a Lewis pair formation.^[^ ^15^, ^42^ ^]^

**Scheme 3:**
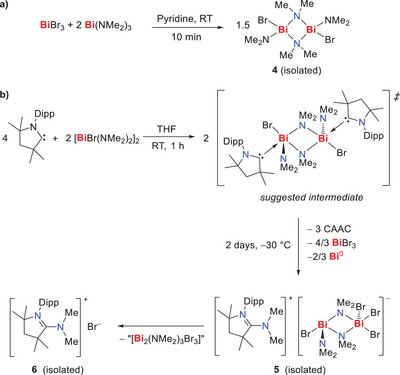
a) Synthesis of diamido bismuth bromide complex **4** and b) its reactivity toward ^Me2^CAAC. The species listed under (or next to) the reaction arrows indicate plausible by‐products, which may undergo further reactions (especially [Bi_2_(NMe_2_)_3_Br_3_], see main text).

To test this hypothesis, we identified the bromo bismuth amide Bi(NMe_2_)_2_Br (**4**) as a suitable model system. This compound could successfully be prepared in 75% isolated yield through a comproportionation reaction between Bi(NMe_2_)_3_ and BiBr_3_ in pyridine (Scheme 3). Schulz and co‐workers have reported a series of mono‐, di‐, and tri‐aminobismuthanes with lower yields, starting from the BiCl_3_ and the lithium *N*‐trimethylsilyl‐amides but their reactivity has not been explored in detail, yet.^[^ ^43^ ^]^

**Figure 3:**
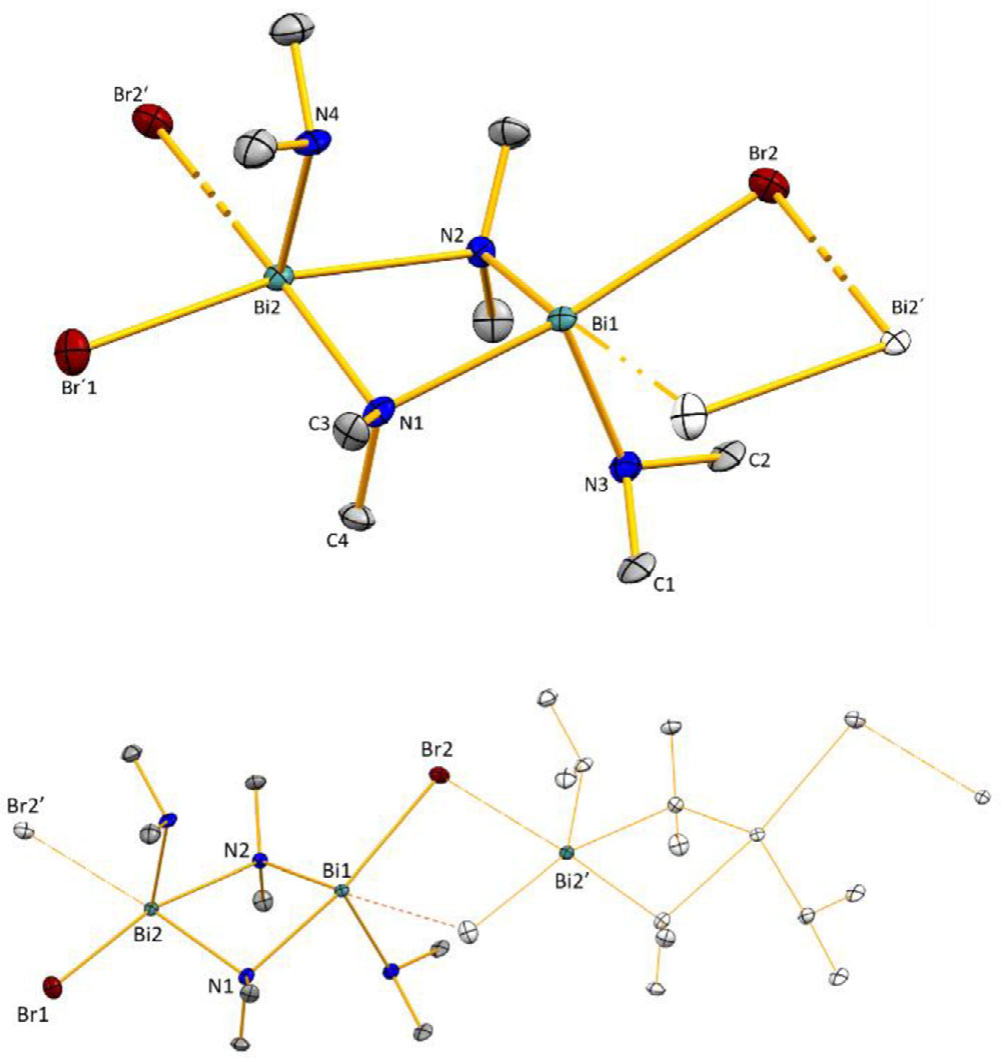
Molecular structure of **4** in the solid state. Displacement ellipsoids are shown at the 50% probability level. Hydrogen atoms are omitted for clarity. Colorless ellipsoids represent atoms exceeding the dinuclear motif. Top: Dinuclear complex **4**. Bottom: arrangement of **4** as a 1‐D coordination polymer in the solid state. Selected bond distances (Å) and angles (°): Bi1─Bi2 3.549(2), Bi1─Br2 2.9489(13), Bi2─Br2’ 3.3038(15), Bi1─Br1’ 3.4800(16), Bi1─N1 2.412(6), Bi1─N2 2.261(6), Bi1─N3 2.130(6), Br2─Bi1 2.9489(13), Bi2─N1 2.307(6), Bi2─N2 2.514(6), Bi2─N4 2.166(6); Bi1─N2─Bi2 95.9(2), Bi1─N1─Bi2 97.5(2), N2─Bi1─Br2 44.81(14), N1─Bi2─Br1 90.45(14), N4─Bi2─Br1 96.22(16), N2─Bi1─N1 78.88(19), N2─Bi2─N1 75.94(19), N3─Bi1─N1 87.6(2).

Compound **4** is sensitive to moisture, temperature, and light. Exposure to light at room temperature for more than 3 hours in both solid and solution resulted in the formation of metallic bismuth. Compound **4** was thoroughly characterized using NMR spectroscopy, mass spectrometry, elemental analysis, and single‐crystal X‐ray diffraction. In the ^1^H NMR and ^13^C NMR spectra in pyridine‐*d* ^5^, the peaks at 4.57 ppm and 45.4 ppm, respectively, were attributed to the amide groups. Orange‐colored single crystals suitable for X‐ray diffraction analysis were obtained by re‐crystallization from a pyridine solution layered with hexane. Here, **4** crystallized in the monoclinic space group *P*2_1_/*n*. The asymmetric unit of compound **4** is best described as a dimer without any additional solvent coordination to the bismuth centers (Figure 3). Each bismuth atom interacts with a bromido ligand of a neighboring formula unit. Overall, this leads to a 1‐D coordination polymer that extends along the crystallographic b‐axis. Each bismuth center adopts a distorted square pyramidal coordination geometry, with μ_2_‐Br and μ_2_‐NMe_2_ ligands in a *cis*‐configuration and an amide group in the apical position. The Bi─N bond lengths in the Bi_2_N_2_ core reveal an asymmetry of the Bi─N─Bi interactions (Bi1–N1/2, 2.412(6) / 2.261(6) Å, and Bi2–N1/2, 2.307(6) / 2.514(6) Å), and an elongation compared to the terminal Bi─N bond lengths (Bi─N^apical^, 2.130(6)–2.166(6) Å).

With compound **4** in hands, its reactivity toward ^Me2^CAAC was tested. Specifically, **4** was added to an equimolar amount of ^Me2^CAAC in THF, resulting in the immediate formation of an intensely blue solution (Scheme 3b). Keeping the solution at low temperature for 2 days yielded yellow crystals of [^Me2^CAAC─NMe_2_]^+^[Bi_2_(NMe_2_)_3_Br_4_]^−^ (**5**), along with a dark precipitate (presumably bismuth black). The Lewis acidic properties of bismuth compounds,^[^ ^15^, ^40^, ^44^, ^45^ ^]^ the Lewis basic nature of the carbene,^[^ ^11^ ^]^ and the color change similar to that in the reactions shown in Scheme 2 let us suggest the initial formation of the Lewis acid/base adduct [Bi(^Me2^CAAC)(NMe_2_)_2_Br]. The formation of **5** clearly indicates that the intermediate is unstable, undergoing NMe_2_ group transfer to the carbene carbon atom. Interestingly, the yellow solution of **5** slowly decomposes further, producing colorless crystals of [CAAC─NMe_2_]^+^Br^−^ (**6**) along with unidentified bismuth compounds.^[^ ^46^ ^]^ Both **5** and **6** were isolated and characterized by single crystal X‐ray diffraction, NMR spectroscopy, and mass spectrometry. Due to high sensitivity toward temperature and light, we could not get a satisfactory elemental analysis data for **5**, but **6** was characterized by elemental analysis. In the ^1^H NMR spectra of both, **5** and **6**, the two methyl peaks of the NMe_2_ groups resonate in different regions due to their distinct environments and hindered rotation about the C─NMe_2_ bond, as confirmed by NOESY‐NMR spectroscopic analyses. The presence of the [CAAC─NMe_2_]^+^ cation was further confirmed by the molecular ion peaks at *m/z* 329.2956 and 329.2945 in HR‐MS spectra of **5** and **6**, respectively. ^15^N NMR spectroscopy on compound **6** confirms the expected π‐contributions in the Me_2_N‐C^CAAC^ bond (δ = −230.3 (N^CAAC^) ppm).^[^ ^19^ ^]^

**Figure 4:**
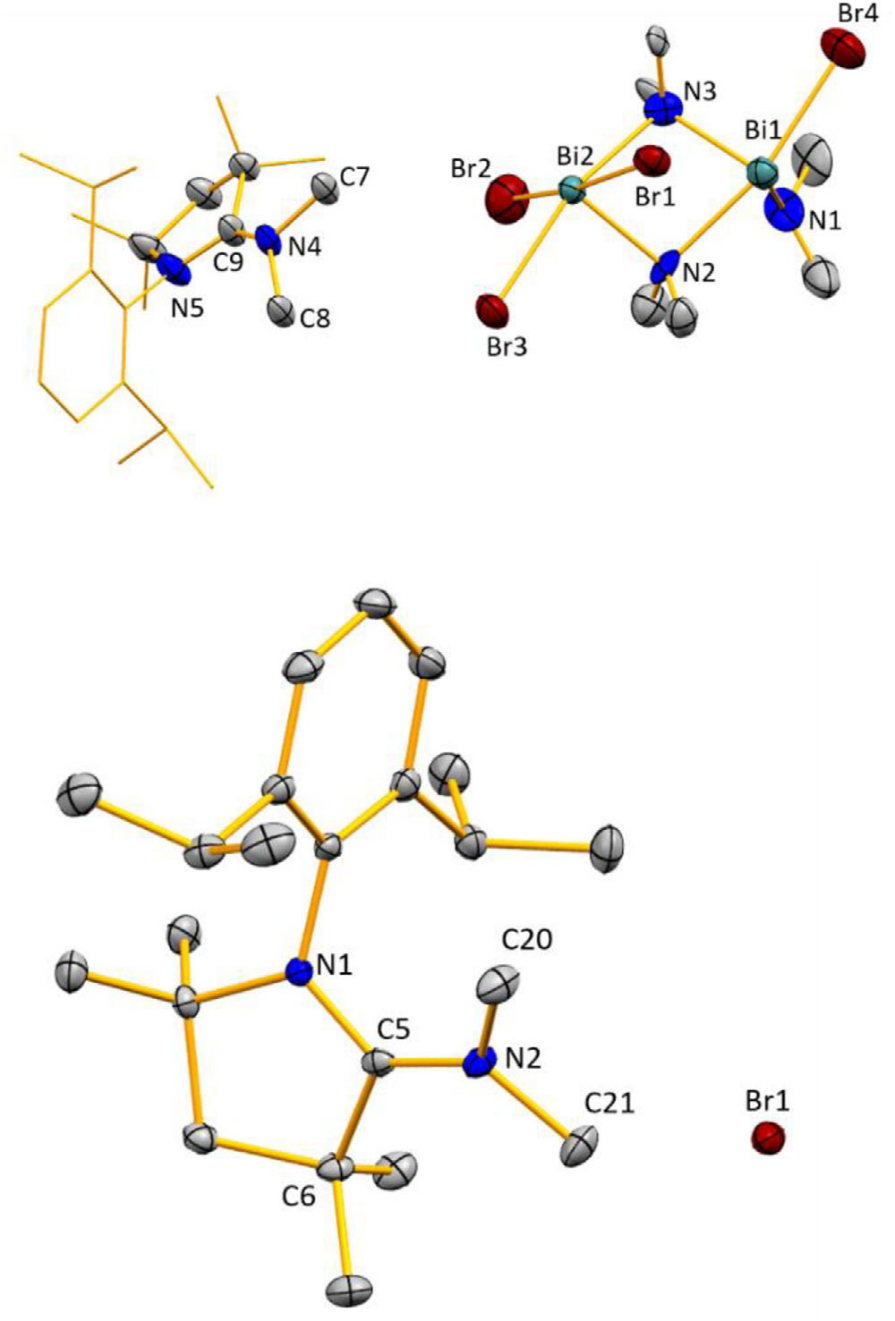
The molecular structure of **5** (above) and **6** (down). Hydrogen atoms are omitted for clarity. Selected bond distances (Å), bond angles (deg) and torsion angle (deg): for **5**: C9─N4 1.31(2), C9─N5 1.36(3), N4─C7 1.49(3), N4─C8 1.48(3), Bi1─Bi2 3.5953(10), Bi1─Br4 2.790(3), Bi12─Br1 2.952(2), Bi2─Br2 2.715(3), Bi2─Br3 2.862(2), Bi1─N2 2.517(17), Bi2─N2 2.309(13), Bi1─N3 2.258(16), Bi2─N3 2.488(17), Bi1─N1 2.190(15); N2─Bi2─N3 76.8(5), Bi2─N2─Bi1 96.2(6),N2─Bi1─N3 77.1(5), Bi1─N3─Bi2 98.4(6), N4─C9─N5 123(2), C9─N4─C7 121(2), C8─N4─C7 111(3). For **6**: N1─C5 1.3391(16), C5─N2 1.3219(16), C6─C5 1.5297(17), N2─C20 1.4710(16), N2─C21 1.4680(16); N1─C5─C6 111.84(11), N1─C5─N2 123.82(11), C6─C5─N2 124.30(11), C20─N2─C21 113.44(11).

The formation of **5** is unequivocally confirmed by single‐crystal X‐ray diffraction studies (Figure 4; triclinic space group *P* $\bar{1}$). In the anionic part of compound **5**, one bismuth atom is pentacoordinate, while the other bismuth atom is tetracoordinate. The core Bi_2_N_2_ structure present in the starting material **4** is retained in **5**. The Bi─N bond lengths in the Bi_2_N_2_ core reveal an asymmetry of the Bi─N─Bi interactions (Bi1─N2/3, 2.517(17)/2.258(16) Å, and Bi2─N2/3, 2.307(6)/2.488(17) Å), and an elongation compared to the terminal Bi─N bond lengths (Bi─N^apical^, 2.190(15) Å), which is very similar to the bonding scheme found in compound **4**.

Compound **6** crystallizes in the monoclinic *P*2_1_/*n* space group (Figure 4). The central carbon atom (C5) is tricoordinate and shows a trigonal planar coordination geometry, with bond angles N2─C5─N1 at 123.82(11)°, N2─C5─C6 at 124.30(11)°, and N1─C5─C6 at 111.84(11)° (angle sum around C5: 359.96(11)°). The N2─C^CAAC^ distance (1.3219(16) Å) is marginally shorter than the C5─N1 distance (1.3391(16) Å), which indicates partial multiple bond character in both cases, confirming the results of spectroscopic analyses.

**Figure 5:**
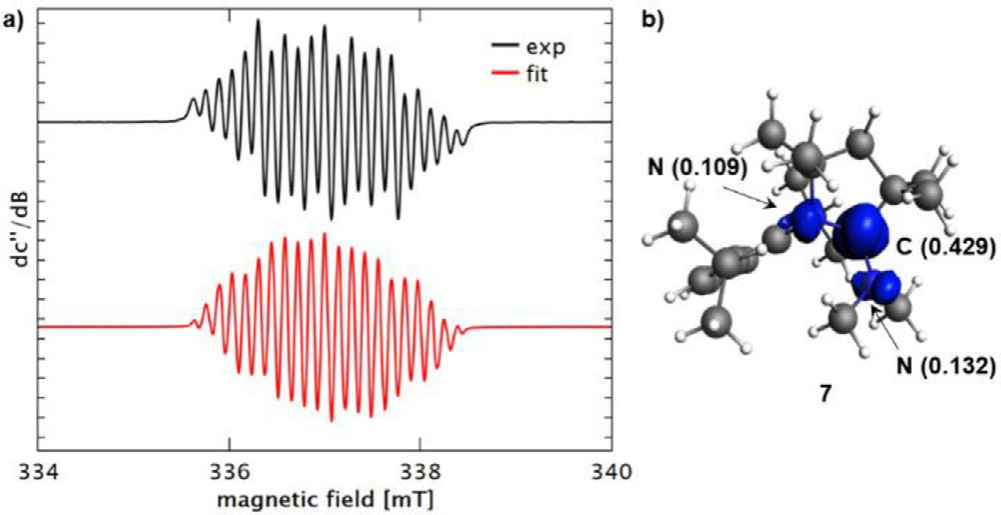
a) Experimental (black) and simulated (red) continuous‐wave (CW) X‐band EPR spectra of a solution containing 1 equiv. BiBr(NMe_2_)_2_ (c = 7.0·10^−2^ mol/L) and 1 equiv. ^Me2^CAAC in THF. b) Spin density plot of **7** at an isovalue of 0.005 as determined by DFT calculations (spin density at selected atoms given in parentheses).

**Scheme 4:**
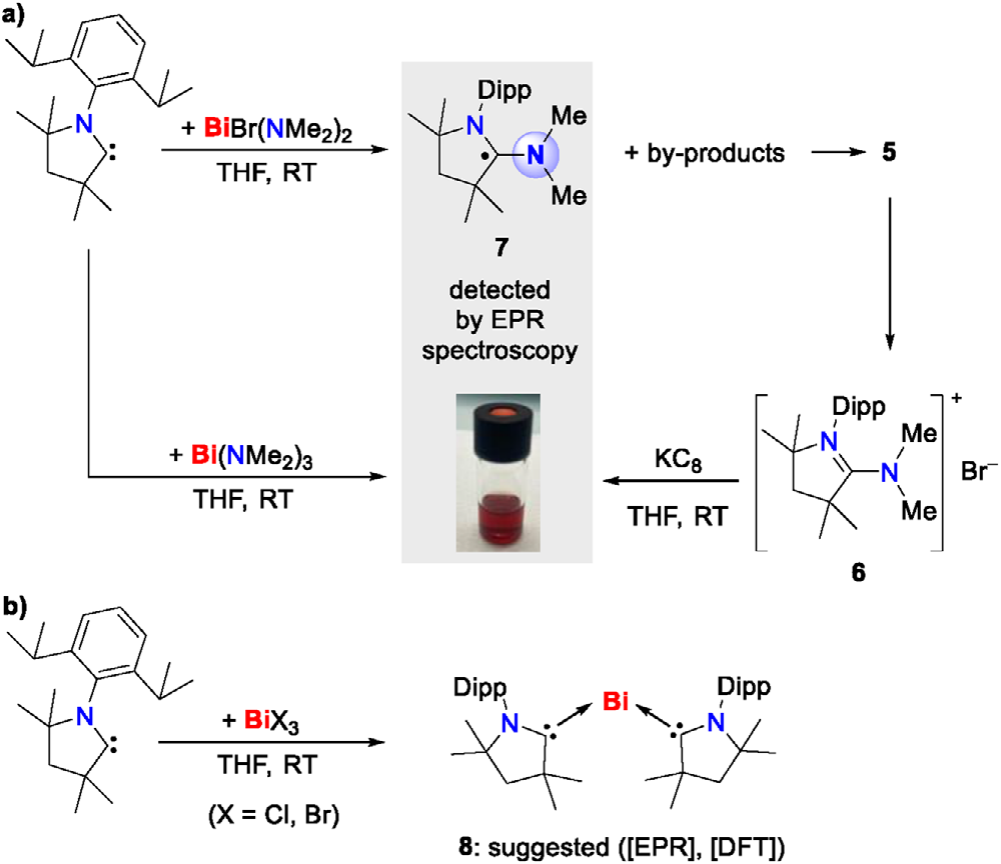
a) In situ generation of **7** via three independent approaches. b) EPR spectroscopic monitoring of the reaction between ^Me2^CAAC and BiX_3_ (X = Cl, Br).

**Figure 6:**
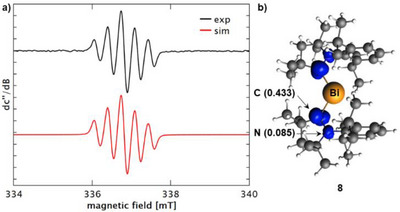
a) Experimental (black) and simulated (red) CW X‐band EPR spectra of a solution containing 1 equiv. BiBr_3_ (c = 3·10^−2^ mol/L) and 1 equiv. ^Me2^CAAC in THF. b) Spin density plot of **8** at an isovalue of 0.005 as determined by DFT calculations (spin density at selected atoms given in parentheses).

Remarkably, the formation of the [CAAC─NMe_2_]^+^ cation in compounds **5** and **6** is formally obtained through the transfer of a nitrenium group, [NMe_2_]**^+^**, from bismuth to carbon. Both a concerted reductive elimination of [CAAC─NMe_2_]^+^ from the suggested intermediate [Bi(^Me2^CAAC)(NMe_2_)_2_Br] and a stepwise reaction through a single‐electron transfer pathway seem plausible.^[^ ^47^, ^48^, ^49^, ^50^ ^]^ Thus, the reaction of ^Me2^CAAC and **4** in THF was monitored by EPR spectroscopy. At early stages of the reaction, a mixture of at least two different radical species was present, changing their relative intensity ratios as the reaction proceeds. After 21 hours at room temperature, a single species was finally observed, which showed hyperfine coupling of the unpaired spin with two nitrogen atoms and two methyl groups, all of which were spectroscopically inequivalent: a(1 × ^14^N) = 15.8 MHz, a(1 × ^14^N) = 11.3 MHz, a(3 × ^1^H) = 4.29 MHz, a(3 × ^1^H) = 3.23 MHz. The *g*_iso_ value amounts to 2.0026 (Figure 5). This resonance was assigned to the radical compound [CAAC─NMe_2_]^•^ (**7**), in agreement with theoretically predicted EPR spectroscopic parameters of this compound (Scheme 4a, Figure 6, Supporting Information). DFT analysis revealed the spin density to be mainly centered at the former carbene carbon atom (0.429) with less pronounced, but still significant contributions by the N^Dipp^ atom (0.109) and the N^Me2^ atom (0.132) (Figure 5). The persistent nature of **7** is reflected by a half‐life time of > 3 days at room temperature in dilute THF solution according to EPR spectroscopic spin count experiments.

Pioneering work by the group of Lee has significantly advanced the field by developing NHC‐stabilized nitroxyl, aminyl, and triazenyl radicals, which are synthetically challenging to access.^[^ ^51^, ^52^, ^53^ ^]^ While [CAAC‐PR₂]^•^ radicals—so‐called α‐phosphanyl radicals—were successfully isolated by Alcarazo and co‐workers in 2017, simple carbene‐based α‐aminyl radicals have not yet been reported to date.^[^ ^54^ ^]^

As an important cross‐check, the reaction of Bi(NMe_2_)_3_ with ^Me2^CAAC in THF was also monitored by EPR spectroscopy, revealing a resonance identical to that of **7** after 5.5 hours reaction time (Scheme 4a). Importantly, the [CAAC─NMe_2_]^+^ ion was also detected by HR‐MS analyses of reactions between Bi(NMe_2_)_3_ and ^Me2^CAAC, demonstrating that this reaction pathway does not require the presence of halide ions. To further confirm the formation of the [CAAC─NMe_2_]**·** (**7**) radical, [CAAC─NMe_2_]^+^ Br^−^ (**6**) was envisioned as a starting material in a reductive approach. Cyclic voltammograms (CVs) of **6** (Figure S27) exhibited a quasi‐reversible one‐electron reduction at *E*_1/2_ = 2.75 V versus *Fc*/*Fc* ^+^. Spurred‐on by these results, **6** was reacted with KC_8_ in THF, which resulted in the immediate formation of a suspension with a dark‐pink liquid phase. The work‐up afforded a pink oil as a crude product. While the presence of **7** in the crude product was confirmed by EPR spectroscopy (and was in agreement with HR‐MS data and elemental analyses), NMR data and magnetic susceptibility measurements (Evans’ method) indicated the presence of significant amounts of diamagnetic impurities. Thus, despite a half‐life time of > 3 days in dilute solution (*vide supra*) compound **7** proved too sensitive to be isolated in pure form so far.

These findings motivated us to also check for potential radical intermediates in the reaction leading to compounds ([^Me2^CAAC─X]^+^)_2_[Bi_2_X_8_(thf)_2_]^2−^ (X = Cl (**2**), Br (**3**)). Indeed, a resonance could be detected, which was identical in both cases (Figure 6). The signal could be modeled taking into account hyperfine coupling of the unpaired spin with two spectroscopically equivalent nitrogen nuclei showing coupling constants of a(2 × ^14^N) = 9.58 MHz and a g_iso_ value of 2.0039. In agreement with the identical nature of the spectra obtained in reactions starting from either BiCl_3_ or BiBr_3_, all structural models with halide atoms that we considered were not in congruency with the experimentally observed EPR spectrum according to DFT calculations. Specifically, [BiX_2_(CAAC)_2_]^•^ (both isomers with X in *cis*‐ and in *trans*‐position), [BiX(CAAC)_2_]^+•^, and [X(CAAC)_2_]^•^ with X = Cl, Br were investigated (Supporting Information, Figure S33). While radical cations derived from the one‐electron oxidation of tethered CAAC‐CAAC dimers have been reported,^[^ ^55^ ^]^ the potential formation of radical cations of the type [(CAAC)_2_]^+•^ was ruled out, since the oxidation of ^Me2^CAAC with NOSbF_6_ in varying stoichiometries did not reproduce the spectrum shown in Figure 6. Surprisingly, the spin density plot of the calculated species [Bi(CAAC)_2_]^•^ (**8**) promised to properly reproduce the spectrum that was experimentally observed, while also offering an explanation for the observed species being independent of the bismuth source (BiCl_3_ versus BiBr_3_; Scheme 4b, Figure 6b). More detailed theoretical analysis confirmed that a compound with the stoichiometry [Bi(CAAC)_2_] shows a doublet ground state, which is favored over the quartet state by 28.7 kcal·mol^−1^. The spin density is evenly distributed over the two CAAC ligands, predominantly at the C^Carbene^ atom (0.429 and 0.433), with significant spin density being also localized at the nitrogen atoms (0.084 and 0.085). Thus, the electronic structure analysis of **8** confirms that the unpaired electron is predominantly delocalized between the two vacant p‐orbitals of the two CAAC ligands (Figure 6b). The calculated EPR spectroscopic parameters are in good agreement with experimental values, showing a close‐to‐zero coupling constant for the bismuth atom (Table S17). In the course of the reaction between CAAC and BiX_3_, compound **8** is tentatively suggested as a fleeting intermediate en route to elemental bismuth and free CAAC. This is in agreement with a reduction wave at moderate potentials of ca. −1.0 V versus *Fc*/*Fc* ^+^ in the CV of [Bi(CAAC)_2_]^+^ reported by Roesky and co‐workers.^[^ ^18^ ^]^

## 3 Conclusion

In conclusion, this study elucidates the complex reactivity patterns between CAACs and bismuth(III) compounds. A simple adduct of a CAAC with a bismuth trihalide was isolated and for the first time fully characterized, revealing visible‐light‐induced electronic transitions from the largely bismuth‐ and halogen‐based HOMO to the carbene‐based LUMO. Its metastable nature was tracked down to the formal halonium ion transfer from a bismuth atom to the carbene to give [CAAC─X]^+^ (X = Cl, Br). This likely involves radical intermediates such as the tentatively suggested [Bi(CAAC)_2_]^•^. When bismuth amides (containing Bi─NR_2_ bonds) were exposed to CAACs, formal nitrenium (i.e., [NR_2_]^+^) ion transfer from the bismuth atom to the carbene takes place, which was demonstrated to involve unprecedented α‐aminyl radicals [CAAC─NR_2_]^•^ as intermediates, which form isolable products via one‐electron oxidation, with Bi(III) acting as an electron sink. This unforeseen pathway to α‐aminyl radicals will help to shed light on the properties of this class of highly reactive compounds, which includes a rich variety of synthetically important representatives.^[^ ^56^, ^57^, ^58^ ^]^

Overall, our findings uncover unusual reactivity patterns in the carbene chemistry of compounds with heavy p‐block elements, including a rich variety of radical and one‐electron‐transfer reactions in the absence of external redox reagents. It is anticipated that these results will aid in the design of new carbene‐stabilized Lewis acidic compounds of heavy elements and will stimulate the exploitation of their unusual reactivity patterns.

## Supporting Information

Please see the Supporting Information for experimental details, crystallographic details, quantum chemical data, and the relevant spectroscopic data. CCDC 2420687–2420692 (**1)**, (**2**), (**3**), (**4**), (**5**), and (**6**) contain the supplementary crystallographic data for this paper. These data can be obtained free of charge from The Cambridge Crystallographic Data Centre. The authors have cited additional references within the Supporting Information.^[^ ^59^, ^60^, ^61^, ^62^, ^63^, ^64^, ^65^, ^66^, ^67^, ^68^, ^69^, ^70^, ^71^, ^72^, ^73^, ^74^ ^]^

## Conflict of Interests

The authors declare no conflict of interest.

## Supporting information



Supporting Information

## Data Availability

The data that support the findings of this study are available in the supplementary material of this article.
