# Supplementary material for: The Careful Combination of Bismuth Compounds and CAAC: Formal Halonium and Nitrenium Transfer via Radical Pathways
Source: Chemistry. 2025 Apr 17;31(28):e202500913. doi: 10.1002/chem.202500913 (PMC12089909; doi:10.1002/chem.202500913)
Supplement: Supplementary file 1 — Supporting Information [file CHEM-31-e202500913-s001.pdf]

Table of Contents

1. Experimental section……………………………………………………………………S2
2. NMR and mass spectra of isolated compounds…………………………………….….…. S7
3. Liquid state UV-Vis spectra of **1**, **3** and **3’** and thermochromism of **1**………………... S19
4. Cyclic voltammetry of **6**…………………………………………..…………………. S21
5. EPR spectroscopy………………………………………………………………………. S22
6. Bond lengths and angles of **1-6** as determined by single-crystal XRD……..………… S24
7. Crystallographic data for the structural analysis of compounds **1-6**…………………...S32
8. Quantum chemical calculations………………………………………………………... S35

1. Experimental Section

All air- and moisture-sensitive steps have been carried out under an inert atmosphere of purified argon (5.0) using standard Schlenk techniques and a glovebox (GS systems) up to the point when an aqueous workup was started (if applicable). Solvents were dried over the appropriate drying agents, distilled, and stored over 3 or 4 Å molecular sieves. Bismuth(III) chloride was sublimed prior to use. $Bi(NMe_2)_3$ and ^{Me2}CAAC were prepared according to literature protocols.[10,59] Commercially available Bismuth(III) bromide was used as received. NMR spectra were recorded on Bruker AV400, AV500, or Bruker 500 HD spectrometers and referenced against residual ^{1}H and ^{13}C signals of the respective deuterated solvent. ^{15}N NMR data of **1**, **2**, **3**, **6** were obtained from ^{15}N-^{1}H HMBC measurements using CH_3NO_2 as a standard.[19] ^{15}N NMR spectra were recorded using a Bruker Avance III HD 600 MHz spectrometer. LIFDI-mass spectra were recorded on a JEOL Accu TOF GCv time-of-flight mass spectrometer. Single-crystal X-ray diffraction data were recorded on a Bruker D8 Quest diffractometer equipped with a Photon 100 detector using Mo−Kαradiation.

Synthesis of [^{Me2}CAAC–$BiCl_3$]$_2$ (1). One equivalent of $BiCl_3$ (0.055 g, 0.18 mmol) and ^{Me2}CAAC (0.050 g, 0.18 mmol) were taken in a vial and THF (2 mL) was added to the reaction mixture. The color of the reaction mixture immediately changed from colorless to green, and the mixture was kept for crystallization at room temperature. Green crystals suitable for single crystal X-ray diffraction analysis were obtained after 24 h. The solution part was decanted and the solid crystals of **1** were isolated and dried in vacuo. Yield: 63 mg, 0.052 mmol, 60 %.

^{1}H NMR (600 MHz, 298 K, CD_2Cl_2): δ = 1.30 (d, *J* = 6.54 Hz, 6 H, CH(C*H*$_3$)$_2$), 1.39 (d, *J* = 6.54 Hz, 6 H, CH(C*H*$_3$)$_2$), 1.57 (s, 6 H, NCC (C*H*$_3$)$_2$), 1.92 (s, 6 H, NC(C*H*$_3$)$_2$), 2.24 (s, 2 H, CC*H*$_2$N), 2.89 (sept, 2 H, C*H*(C*H*$_3$)$_2$), 7.35 (d, *J* = 7.81 Hz, 2 H, Dipp-*m*-H), 7.50 (d, *J* = 7.81 Hz, 1 H, Dipp-*p*-H) ppm. **^{13}C{^{1}H} NMR (126 MHz, 298 K, CD_2Cl_2):** δ = 26.4 (*i*Pr-*C*H$_3$), 27.6 (*i*Pr-*C*H$_3$), 29.0 (*i*Pr-*C*H), 29.6 (NCC(*C*H$_3$)$_2$), 30.6 (NC(*C*H$_3$)$_2$), 61.4 (*C*H$_2$), 68.3 (N*C*(CH$_3$)$_2$), 88.2 (NC*C*(CH$_3$)$_2$), 126.82 (Dipp-*m*-*C*H), 128.8 (Dipp-*p*-*C*H), 131.5 (Dipp-*i*-*C*), 146.3 (Dipp-*o*-*C*) ppm. **^{15}N–^{1}H HMBC NMR (61 MHz, 298 K, CD_2Cl_2):** δ = –137.3 ppm. **HR-MS (LIFDI, pos.):** Calc. for ($^{12}C_{20}{}^{1}H_{31}{}^{209}Bi^{35}Cl_2{}^{14}N_1$)$^{+}$ ([**1**-Cl]$^{+}$): m/z = 564.16374, found m/z = 564.16274 . **Elemental analysis (%),** calc. for $C_{20}H_{31}BiCl_3N_1$ (600.81 gmol^{-1}): C 39.98, H 5.20; N 2.33; found: C 39.87, H 5.43, N 2.57.

Compound **1** was also obtained, when reacting $BiCl_3$ and ^{Me2}CAAC in a 1:2 molar ratio.

Solid samples of **1** can be stored at low temperature (–30 °C) without degradation being observed over a period of at least a several weeks. However, solid samples of **1** and solutions of **1** in THF slowly degrade to form black solids along with **2**.

Synthetic access to compound **1** has previously been reported with ^{1}H and ^{13}C NMR spectroscopic analysis as the only means of characterization, and was used as a starting material for successful follow-up chemistry.[14] In the previous contribution, **1** has been reported as a white, analytically pure precipitate. When reproducing the literature-reported protocol, in which the reaction is first started at –78 °C using Et_2O as the solvent and then warmed to ambient temperature during work-up, the product precipitated as a colorless powder, but turned pale greenish-yellow during work-up. To our surprise, the solid sample of **1** proved to show thermochromism, being colorless at low temperature and greenish-yellow at ambient temperature (see section "3. Liquid state UV-Vis spectra of **1**, **3** and **3'** and thermochromism of **1**" in the SI). NMR spectroscopic analyses of the sample obtained in this work were identical to those previously reported.[14] Powder X-ray diffraction analysis of the sample obtained from the reproduced literature method[14] gave the same pattern that was predicted based on the single-crystal X-ray analysis of a sample obtained from the procedure reported in this work.

In general, the choice of a rather weakly- to non-polar solvent appears to be beneficial when targeting the isolation of CAAC adducts of bismuth compounds, in order to precipitate the desired product. Keeping the targeted adduct in solution for extended periods of time should be avoided, since radical transfer appears to be a viable reaction pathway. Of course, the deliberate exploitation of this reaction pathway (beyond the mere isolation of bismuth-carbene adducts) is a promising approach.

Synthesis of ($[^{Me2}CAAC–Cl]^+)_2[Bi_2Cl_8(thf)_2]^{2–}$ (2). One equivalent of $BiCl_3$ (0.055 g, 0.18 mmol) and ^{Me2}CAAC (0.050 g, 0.18 mmol) were taken in a vial and of THF (2 mL) was added to the reaction mixture. The color of the reaction mixture changed from colorless to green. At room temperature over 7 days, compound **1** undergoes degradation in THF and forms colorless needle-shaped crystals of ($[^{Me2}CAAC–Cl]^+)_2[Bi_2Cl_8(thf)_2]^{2–}$ (**2**). The solvent was decanted and the solid crystals were washed with hexane (2 × 5 mL) and dried in vacuo. Yield: 52 mg, 0.035 mmol, 65%.

^{1}H NMR (500 MHz, 298 K, CD_2Cl_2): 1.17 (d, J = 6.75 Hz, 6 H, CH(CH_3)$_2$), 1.38 (d, J = 6.75 Hz, 6 H, CH(CH_3)$_2$), 1.69 (s, 6 H, NCC(CH_3)$_2$), 1.81 (s, 6 H, NC(CH_3)$_2$), 2.53 (sept, 2 H, CH(CH_3)$_2$), 2.99 (s, 2 H, CCH_2N), 7.42 (d, J = 8 Hz, Dipp-*m*-H), 7.61 (d, J = 8 Hz, 1 H, Dipp-*p*-H) ppm. **^{13}C{^{1}H} NMR (126 MHz, 298 K, CD_2Cl_2):** δ = 23.7(*i*Pr-*C*H$_3$), 26.4(*i*Pr-*C*H$_3$), 28.7(*i*Pr-*C*H), 29.4 (NCC(*C*H$_3$)$_2$), 30.4 (NC(*C*H$_3$)$_2$), 48.2 (*C*H$_2$), 68.3(N*C*(CH$_3$)$_2$), 85.8(NC*C*(CH$_3$)$_2$), 126.8 (Dipp-*m*-*C*H), 128.2 (Dipp-*p*-*C*H), 132.9 (Dipp-*i*-*C*), 145.1(Dipp-*o*-*C*) ppm. **^{15}N–^{1}H HMBC NMR (61 MHz, 298 K, CD_2Cl_2):** δ = –148.3 ppm. **HR-MS (LIFDI, pos.):** calc. for $(^{12}C_{20}{}^1H_{31}{}^{35}Cl^{14}N)^+$ ($[^{Me2}CAAC–Cl]^+$): m/z = 320.21450, found m/z =320.21205. **Elemental analysis (%),** calc. for $C_{24}H_{39}BiCl_5NO$ (743.13 gmol^{-1}): C 38.75, H 5.29, N 1.88; found: C 38.55, H 5.56, N 2.12.

Synthesis of ([^{Me2}CAAC–Br]$^+$)$_2$[$Bi_2Br_8(thf)_2$]$^{2-}$ (3). One equivalent of $BiBr_3$ (0.079 g, 0.18 mmol) and ^{Me2}CAAC (0.050 g, 0.18 mmol) were taken in a vial and THF (2 mL) was added to the reaction mixture. The color of the reaction mixture immediately changed from colorless to dark blue, and the mixture was kept for crystallization at –30 ℃. Yellow crystals suitable for single crystal X-ray diffraction were obtained after 48 h. The solution part was decanted and the solid crystals of **3** were isolated. Yield: 64.2 mg, 0.035 mmol, 66%.

^{1}H NMR (500 MHz, 298 K, $CDCl_3$): δ = 1.22 (d, *J* = 6.75 Hz, 6 H, CH(C*H*$_3$)$_2$), 1.37 (d, *J* = 6.75 Hz, 6 H, CH(C*H*$_3$)$_2$), 1.71 (s, 6 H, NCC(C*H*$_3$)$_2$), 1.82 (s, 6 H, NC(C*H*$_3$)$_2$)), 2.52 (sept, 2 H, C*H*(C*H*$_3$)$_2$), 3.04 (s, 2 H, CC*H*$_2$N), 7.43 (d, *J* = 8 Hz, 2 H, Dipp-*m*-H), 7.61 (d, *J* = 8 Hz, 1 H, Dipp-*p*-H) ppm. **^{13}C{^{1}H} NMR (126 MHz, 298 K, $CDCl_3$):** δ = 24.1 (*i*Pr-*C*H$_3$), 26.6 (*i*Pr-*C*H$_3$), 29.6 (*i*Pr-*C*H), 29.8 (NCC(*C*H$_3$)$_2$), 30.4 (NC(*C*H$_3$)$_2$), 48.1(*C*H$_2$), 68.3 (N*C*(CH$_3$)$_2$), 87.8 (NC*C*(CH$_3$)$_2$), 126.1 (Dipp-*m*-*C*H), 126.9 (Dipp-*p*-*C*H), 132.8 (Dipp-*i*-*C*), 144.9 (Dipp-*o*-*C*) ppm. **^{15}N–^{1}H HMBC NMR (61 MHz, 298 K, CD_2Cl_2):** δ = –142.2 ppm. **HR-MS (LIFDI, pos.):** calc. for ($^{12}C_{20}{}^1H_{31}{}^{79}Br^{14}N_1$)$^+$ ([^{Me2}CAAC–Br]$^+$): m/z = 364.1634, found m/z = 364.1620. Depending on the exact conditions of crystallization, the compound ([^{Me2}CAAC–Br]$^+$)$_3$[Bi_2Br_9]$^{3-}$ with the same type of cation as in compound **3** may also be obtained, **Elemental analysis (%),** calc. for $C_{64}H_{101}Bi_2Br_{12}N_3O$ (2305.34 gmol^{-1}): C 33.34, H 4.42; N 1.82; found: C 33.23, H 4.75, N 2.41.

Synthesis of [$BiBr(NMe_2)_2$]$_2$ (4). Two equivalents of $Bi(NMe_2)_3$ (200 mg, 0.59 mmol) and one equivalent of $BiBr_3$ (132 mg, 0.30 mmol) were mixed together in pyridine (5 mL). The reaction mixture was stirred for one hour at room temperature and filtered. The filtrate was layered with hexane (0.5 mL) and kept for crystallization at –30 °C in the dark. Yellow block-shaped crystals of **4** were obtained after two days, isolated by decantation and dried in vacuo. Yield: 165 mg, 0.22 mmol, 75 %.

^{1}H NMR (500 MHz, 298 K, Py-d^5): δ = 4.57 (bs, 12 H, N(CH$_3$)$_2$) ppm. **^{13}C{^{1}H} NMR (126 MHz, 298 K, Py-d^5):** δ = 45.4 ppm. Residual amounts of pyridine may be left in the isolated compound, which should be quantified for every batch individually. **LIFDI-MS:** calc. for ($^{12}C_5{}^1H_5Bi^{79}Br^{14}N$)$^+$ ([BiBr(NC_5H_5)]$^{\bullet+}$): m/z = 366.9404, found m/z = 366.9402; calc. for ($^{12}C_{10}{}^1H_{10}Bi^{81}Br^{14}N_2$)$^+$ ([BiBr(NC_5H_5)$_2$]$^{\bullet+}$): m/z = 447.9806, found m/z = 447.9804; calc. for ($^{12}C_{10}{}^1H_{10}Bi^{79}Br^{81}Br^{14}N_2$)$^+$ ([$BiBr_2$(NC_5H_5)$_2$]$^+$): m/z = 526.8986, found m/z = 526.8970. (i.e. ligand exchange processes and radical formation take place under conditions of mass spectrometric measurements). **Elemental analysis (%),** calc. for $C_4H_{12}BiBrN_2$ (C_5H_5N)$_{0.25}$ (773.85 gmol^{-1}): C 14.36, H 3.29, N 7.69; found: C 14.68, H 3.45, N 7.47.

Synthesis of [^{Me2}CAAC–NMe$_2$]$^+$[Bi$_2$Br$_4$(NMe$_2$)$_3$]$^-$ (5). To a solution of ^{Me2}CAAC (200 mg, 0.69 mmol) in THF (5 mL), **4** (259 mg, 0.69 mmol) was added at room temperature, and the color of the solution immediately turned dark blue. The reaction mixture was stirred for one hour at room temperature. Afterwards all volatiles were removed under reduced pressure, and the remaining solid was washed with hexane (2× 5 mL). The blue powder was re-dissolved in THF (3 mL), layered with hexane (1 mL) and kept for crystallization –30 °C. The formation of yellow crystals of **5** was observed after two days, which were isolated by decantation (thereby also removing a dark precipitate) and dried in vacuo. Notably Compound **5** is light- and temperature-sensitive, so the anionic part [Bi$_2$(NMe$_2$)$_3$Br$_4$]$^-$ further undergoes fast decomposition to give colorless crystals of [^{Me2}CAAC·NMe$_2$]$^+$ Br$^-$ (**6**). Yield: 145 mg, 0.12 mmol, 35 % (yield calculated based on bismuth).

^{1}H NMR (500 MHz, 298 K, CD$_2$Cl$_2$): δ = 1.19 (d, *J* = 6.59 Hz, 12 H, CH(C*H*$_3$)$_2$), 1.22 (d, *J* = 6.65 Hz, 12 H, CH(C*H*$_3$)$_2$), 1.37 (s, 6 H, NCC(C*H*$_3$)$_2$), 1.81 (s, 6 H, NC(C*H*$_3$)$_2$), 2.74 (s, 3 H, NC*H*$_3$, *Me group pointing towards the dipp group*), 2.84 (sept, *J* = 6.70 Hz 2 H, C*H*(CH$_3$)$_2$), 3.49 (s, 3 H, NC*H*$_3$*, Me group pointing towards the CMe$_2$ group*), 4.38 (s, Bi(NMe$_2$)$_3$) 7.32 (d, *J* = 7.79 Hz, 2 H, Dipp-*m*-H), 7.50 (d, *J* = 7.68 Hz, 1 H, Dipp-*p-H*) ppm. **^{13}C{^{1}H} NMR (126 MHz, 298 K, CD$_2$Cl$_2$):** 24.8 (*i*Pr-*C*H$_3$), 25.4 (*i*Pr-*C*H$_3$) 29.4(*i*Pr-*C*H), 29.6 (NCC(*C*H$_3$)$_2$), 29.7 (NC(*C*H$_3$)$_2$), 39.2 (BiN*C*H$_3$), 41.57 (BiN*C*H$_3$), 43.7 (N(*C*H$_3$)), 44.9 (N*C*H$_3$), 46.4 (*C*H$_2$), 55.3 (N*C*(CH$_3$)$_2$), 70.9 (NC*C*(CH$_3$)$_2$), 126.7 (Dipp-*m*-*C*H),131.4 (Dipp-*i-C*), 133.6 (Dipp-*p-C*), 146.9 (Dipp-*o-C*), 175.2 (*C*NMe$_2$) ppm. Due to fast degradation of **5** in the deuterated solvent, we could not obtain ^{1}H and ^{13}C spectra without trace amounts of degradation products. **HR-MS (LIFDI, pos.):** calc. for (^{12}C$_{22}$^{1}H$_{37}$^{14}N$_2$)$^+$ ([CAAC–NMe$_2$]$^+$): m/z = 329.29567, found m/z = 329.29569.

Synthesis of [^{Me2}CAAC–NMe$_2$]$^+$Br$^-$ (6). To a solution of ^{Me2}CAAC (200 mg, 0.69 mmol) in THF (5 mL), **4** (259 mg, 0.69 mmol) was added at room temperature, and the color of the solution immediately turned dark blue. The reaction mixture was stirred for 18 hours at room temperature. The solution color changed to yellow along with the formation of black precipitate of metallic bismuth. Afterwards all volatiles were removed under reduced pressure, and the remaining solid was washed with hexane (3× 5 mL). The yellow powder was re-dissolved in THF (3 mL), layered with hexane (2 mL) and kept for crystallization –30 °C. The formation of yellow crystals of **6** was observed after two days. Notably, compound **5** is light- and temperature-sensitive, so the anionic part [Bi$_2$(NMe$_2$)$_3$Br$_4$] further undergoes decomposition to give colorless crystals of [^{Me2}CAAC·NMe$_2$]$^+$ Br$^-$. Yield: 156 mg, 0.38 mmol, 55 % (yield calculated assuming **6** is obtained by degradation of intermediate **5**).

^{1}H NMR (500 MHz, 298 K, CD$_2$Cl$_2$): δ = 1.19 (d, *J* = 6.75 Hz, 6 H, CH(C*H*$_3$)$_2$), 1.33 (d, *J* = 6.75 Hz, 6 H, CH(C*H*$_3$)$_2$), 1.37 (s, 6 H, NCC (C*H*$_3$)$_2$), 1.79 (s, 6 H, NC(C*H*$_3$)$_2$), 2.35 (s, 2 H, CC*H*$_2$N), 2.73 (s, 3 H,

N*CH*$_3$*, Me group pointing towards the dipp group*), 2.83 (sept, 2 H, C*H*(C*H*$_3$)$_2$), 3.47 (s, 3 H, NC*H*$_3$*, Me group pointing towards the CMe*$_2$), 7.32 (d, *J* = 8 Hz, 2 H, Dipp-*m*-H), 7.50 (d, *J* = 8 Hz, 1 H, Dipp-*p*-H) ppm. **^{13}C{^{1}H} NMR (126 MHz, 298 K, CD$_2$Cl$_2$):** δ = 24.8 (*i*Pr-*C*H$_3$), 25.4 (*i*Pr-*C*H$_3$) 29.4(*i*Pr-*C*H), 29.6(NCC(*C*H$_3$)$_2$), 29.8 (NC(*C*H$_3$)$_2$), 44.8 (N(*C*H$_3$)), 45.2 (N*C*H$_3$), 46.3 (*C*H$_2$), 55.3 (N*C*(CH$_3$)$_2$), 70.9 (NC*C*(CH$_3$)$_2$), 126.8 (Dipp-*m*-*C*H),131.5(Dipp-*i*-*C*),146.9 (Dipp-*o*-*C*) ppm. **^{15}N–^{1}H HMBC NMR (61 MHz, 298 K, CD$_2$Cl$_2$):** δ = –230.3(*N*-^{Me2}CAAC), –271.7 (*N*Me$_2$)ppm. **HR-MS (LIFDI, pos.):** calc. for ($^{12}C_{22}{}^{1}H_{37}{}^{14}N_2$)$^+$ ([CAAC–NMe$_2$]$^+$): m/z = 329.2951, found m/z = 329.2945. **Elemental analysis (%),** calc. for $C_{22}H_{37}BrN_2$ (409.46 gmol^{-1}): C 64.56, H 9.11, N 6.84; found: C 64.50, H 9.72, N 7.71.

Synthesis of [^{Me2}CAAC−NMe$_2$]· (7). Potassium on graphite (67.6 mg, 0.50 mmol) was added to a solution of **6** (205 mg, 0.55 mmol) in THF (6 ml) at room temperature and further stirred for 1 h. The solvent was removed in vacuo and the resulting solid extracted with 10 ml of pentane affording the desired product **7** as a pink oil. Yield: 91 mg, 0.28 mmol, 55 %.

HR-MS (LIFDI, pos.): calc. for ($^{12}C_{22}{}^{1}H_{37}{}^{14}N_2$)$^+$ ([CAAC–NMe$_2$]$^+$): m/z = 329.2951, found m/z = 329.2945. **Elemental analysis (%),** calc. for $C_{22}H_{37}BrN_2$ (409.46 gmol^{-1}): C 64.56, H 9.11, N 6.84; found: C 64.50, H 9.72, N 7.71.

2. NMR and mass spectra of isolated compounds

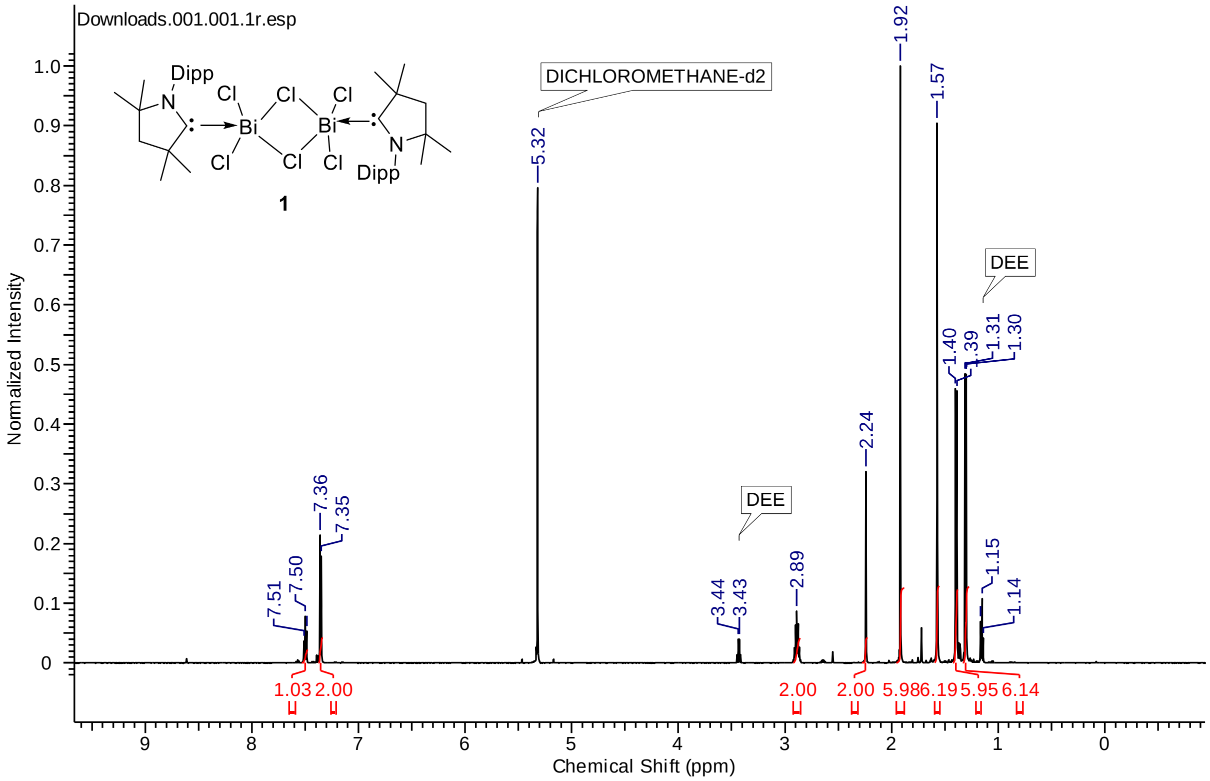

Figure S1. ^{1}H NMR spectrum of **1**.

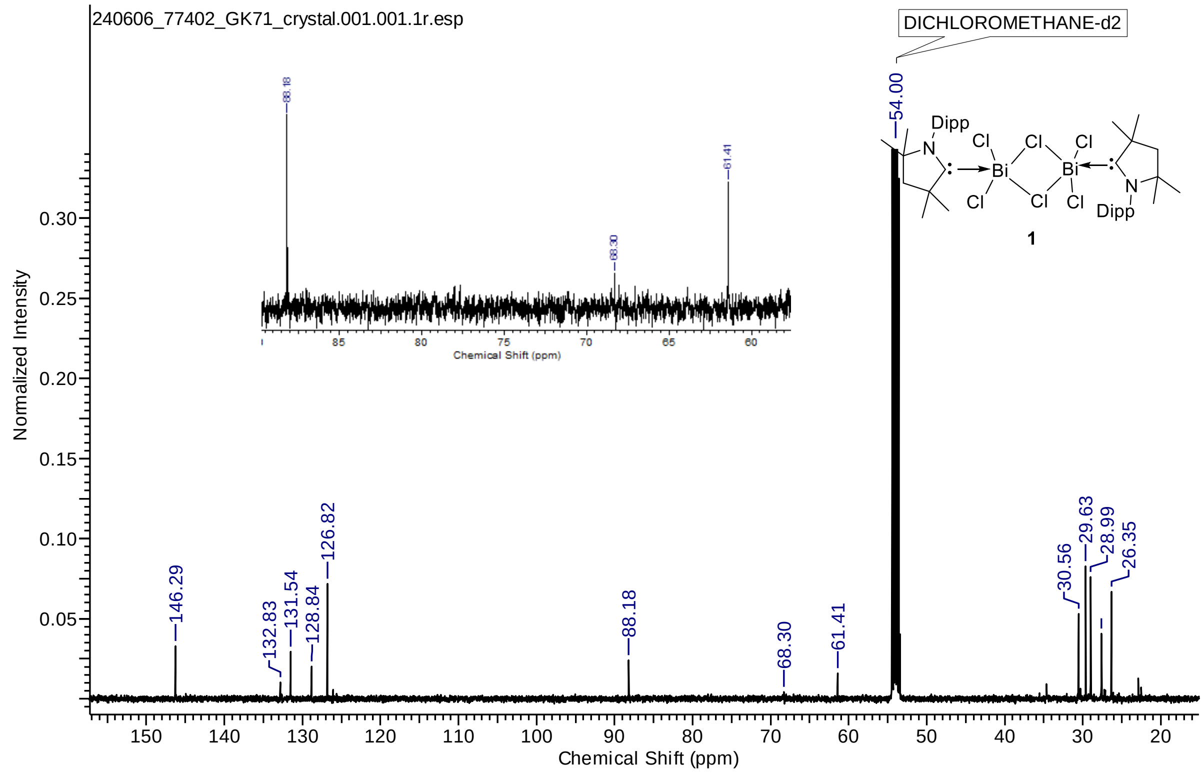

Figure S2. ^{13}C NMR spectrum of **1**.

Data:240502_FD_367_Lb
Comment:
Description:
Ionization Mode:FD+
History:Average(MS[1] 0.71..0.73)

Acquired:14.05.2024 10:30:14
Operator:AccuTOF
m/z Calibration File:FD_Calib_082222_b
Created:16.05.2024 14:33:39
Created by:Lorakis

Charge number:1
Element:^{12}C:0 .. 20, ^{1}H:0 .. 31, ^{209}Bi:0 .. 1, ^{35}Cl:0 .. 2, ^{14}N:0 .. 1
Tolerance:10.00[ppm], 2.00 .. [mDa]
Unsaturation Number:-1.5 .. 50.0 (Fraction:Both)

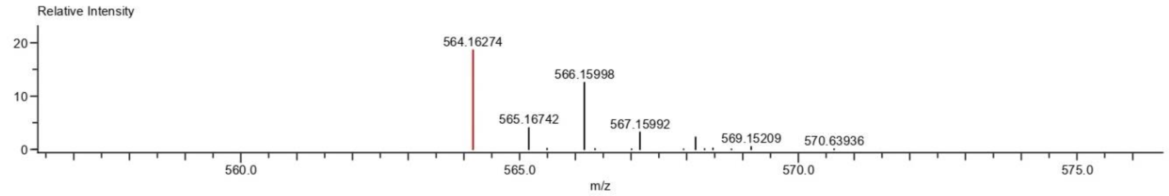

Composition:$C_{20}H_{31}BiCl_2N$
Mono Isotopic Mass:564.16374
Description:
Average Mass:565.35266
Created:16.05.2024 14:33:41
Nominal Mass:564
Created by:Lorakis

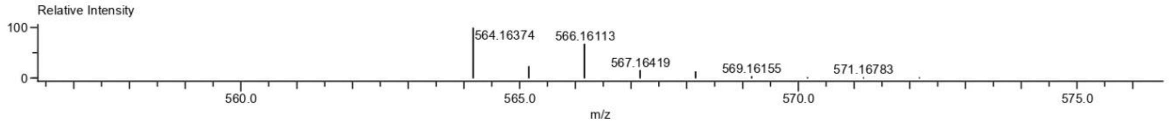

Mass	Intensity	Relative Intensity	Calc. Mass	Mass Difference [mDa]	Mass Difference [ppm]	Possible Formula	Unsaturation Number
564.16274	52594.35	18.58	564.16374	-1.00	-1.76	$^{12}C_{20}{}^{1}H_{31}{}^{209}Bi_1{}^{35}Cl_2{}^{14}N_1$	5.5

Figure S3. HR-MS spectrum of **1**.

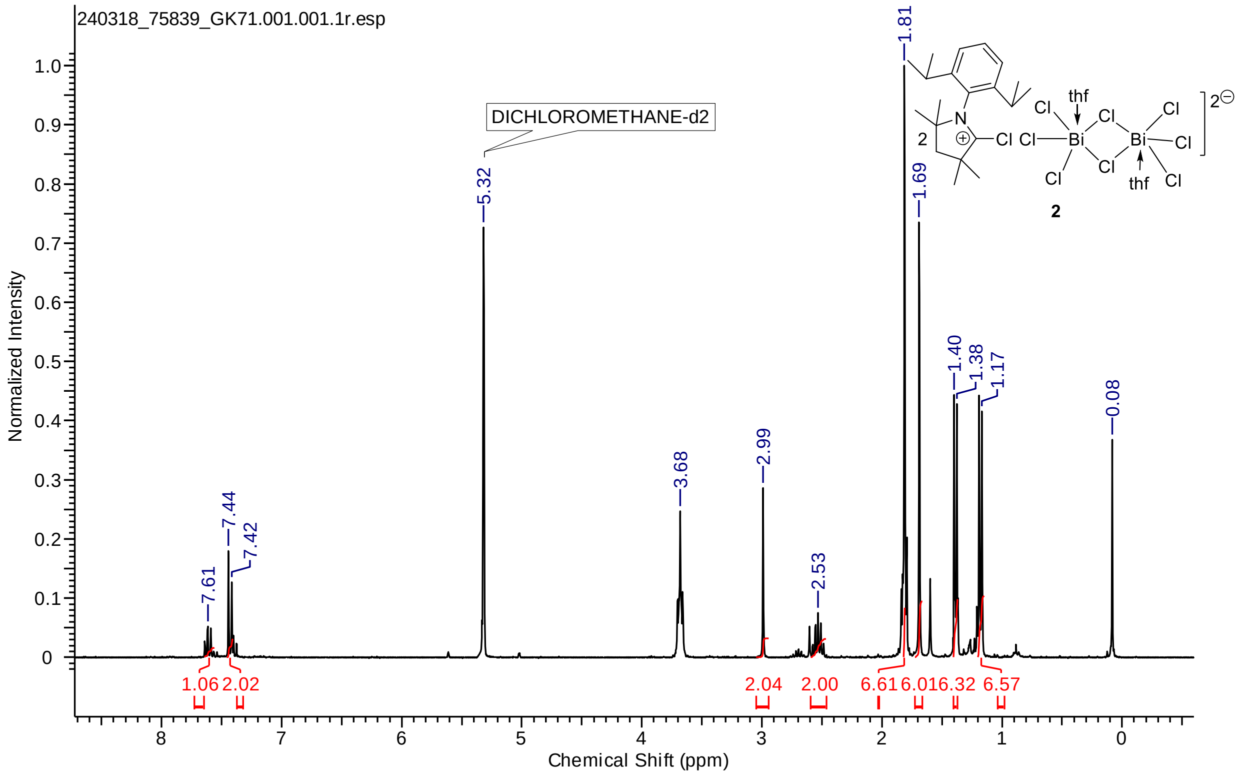

Figure S4. ^{1}H NMR spectrum of **2**.

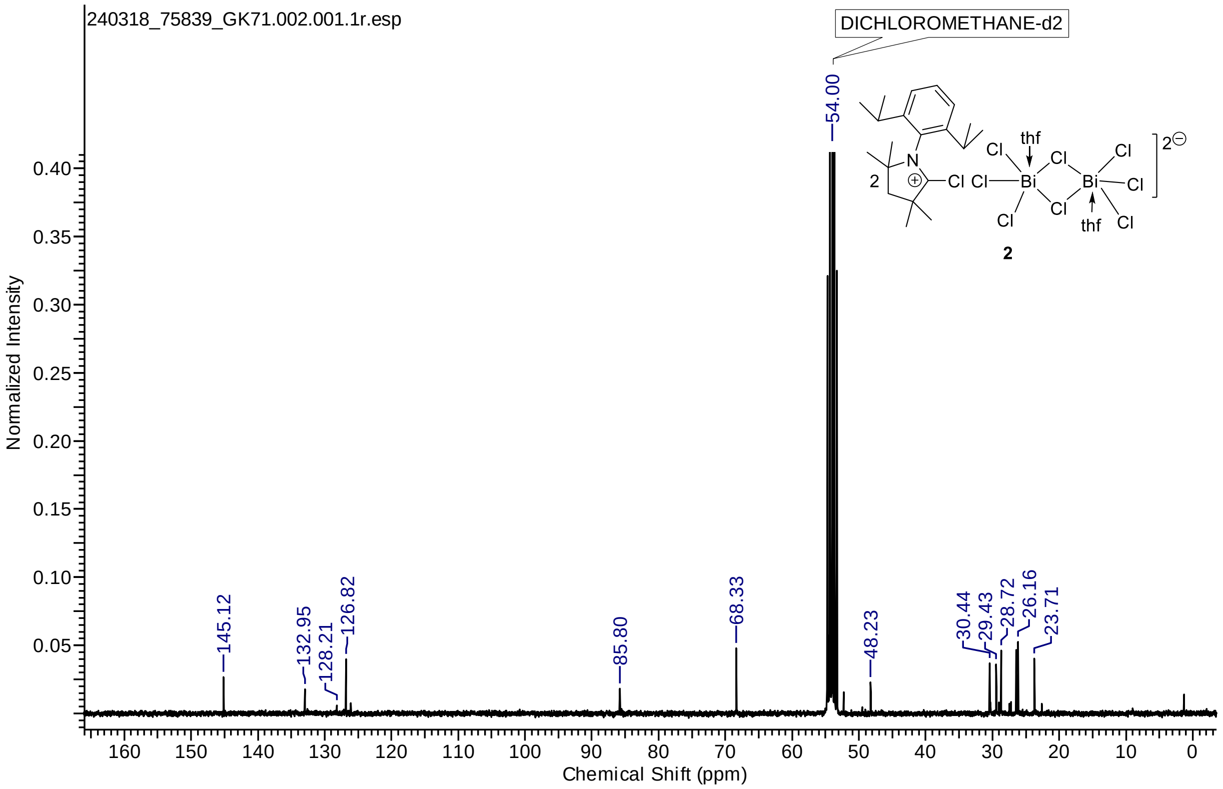

Figure S5. ^{13}C NMR spectrum of **2**.

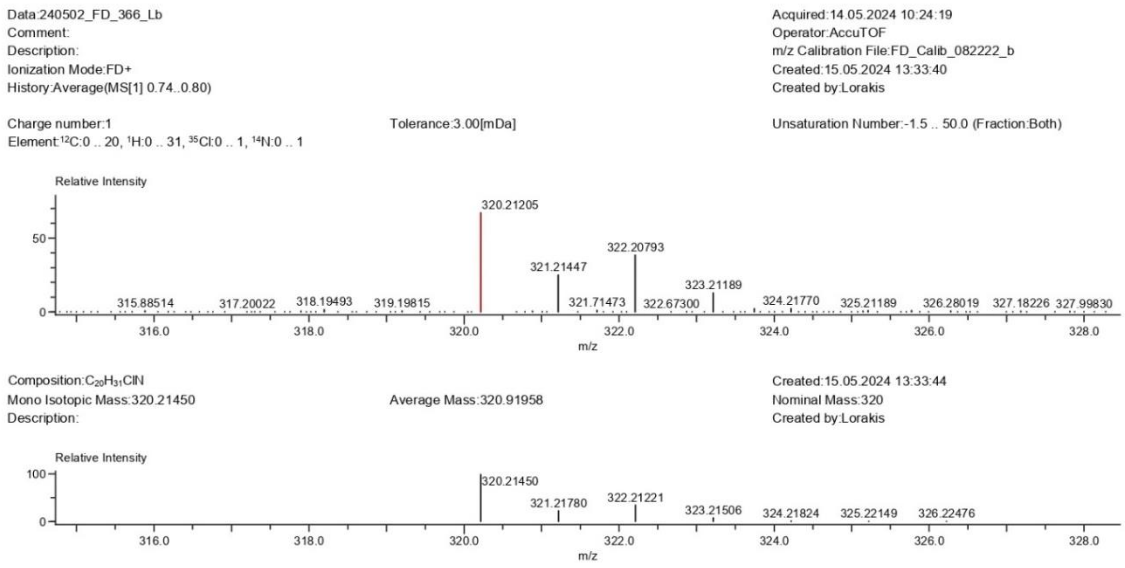

Mass	Intensity	Relative Intensity	Calc. Mass	Mass Difference [mDa]	Mass Difference [ppm]	Possible Formula	Unsaturation Number
320.21205	1344894.47	66.98	320.21450	-2.46	-7.67	$^{12}C_{20}{}^{1}H_{31}{}^{35}Cl_{1}{}^{14}N_{1}$	5.5

Figure S6. HR-MS spectrum of **2**.

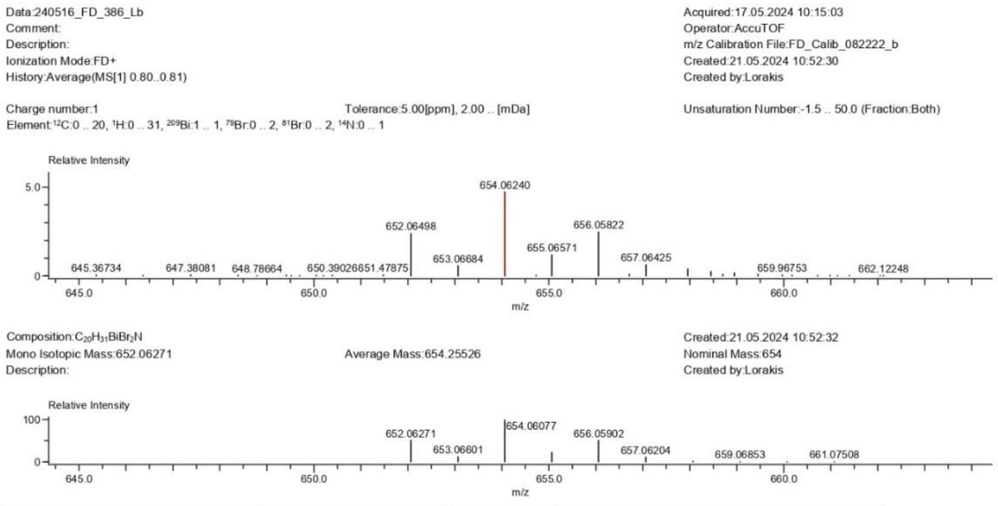

Mass	Intensity	Relative Intensity	Calc. Mass	Mass Difference [mDa]	Mass Difference [ppm]	Possible Formula	Unsaturation Number
654.06240	319850.25	4.70	654.06066	1.74	2.66	$^{12}C_{20}{}^{1}H_{31}{}^{209}Bi_1{}^{79}Br_1{}^{81}Br_1{}^{14}N_1$	5.5

Figure S7. HR-MS spectrum of **3'**.

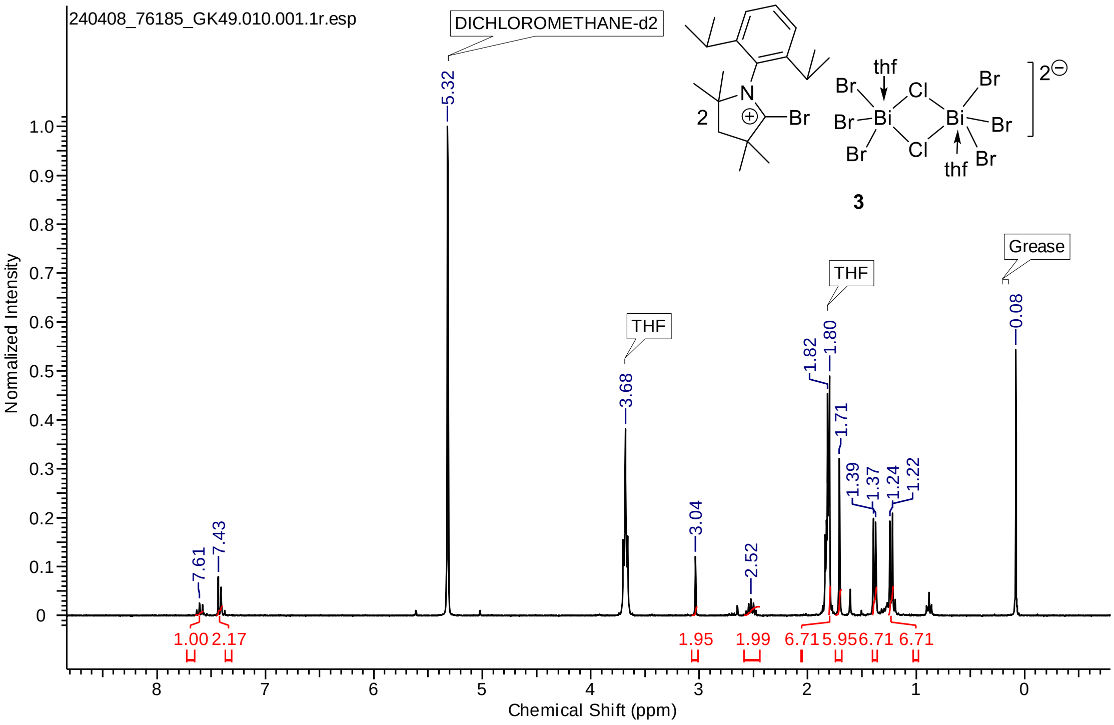

Figure S8. ^{1}H NMR spectrum of **3**.

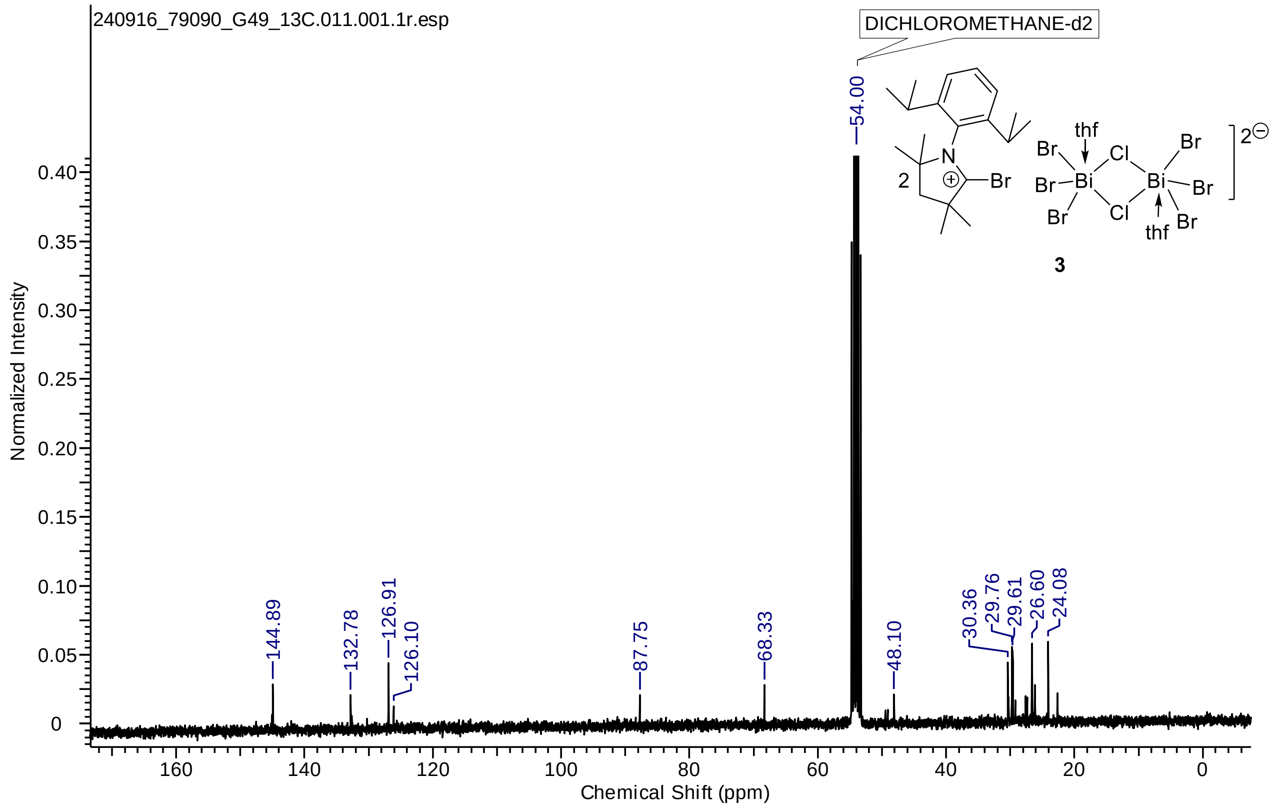

Figure S9. ^{13}C NMR spectrum of 3.

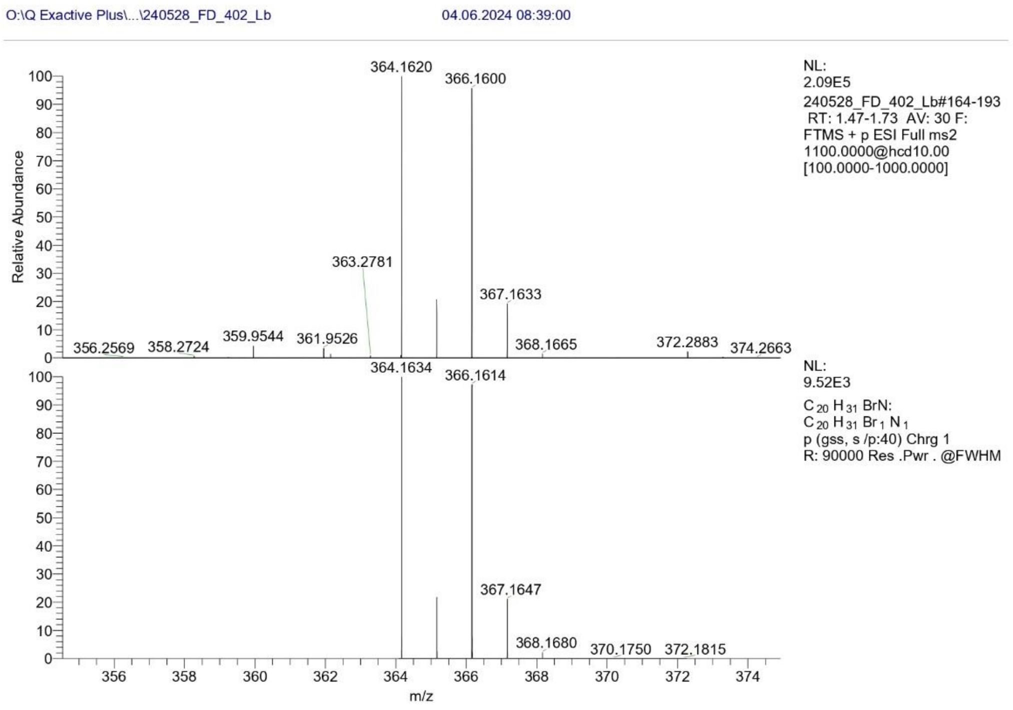

Figure S10. HR-MS spectrum of **3**.

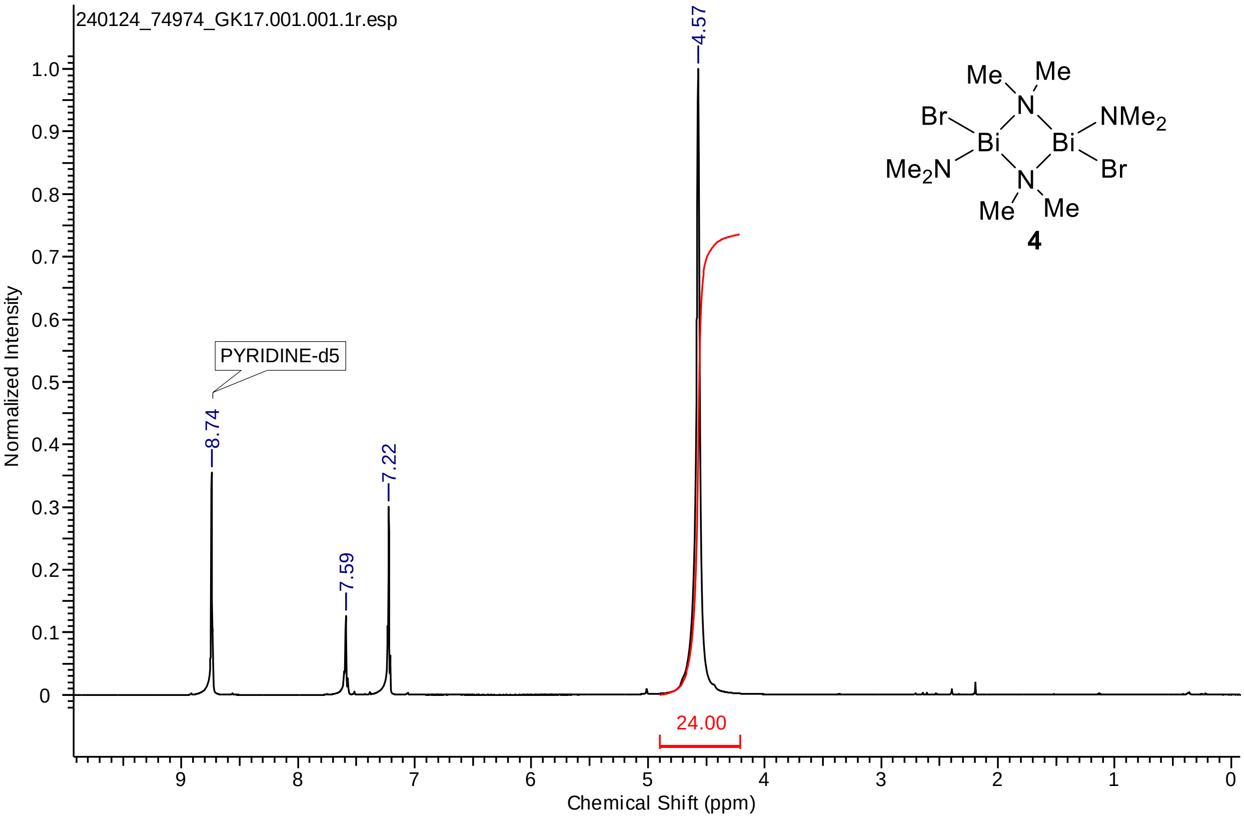

Figure S11. ^{1}H NMR spectrum of **4**.

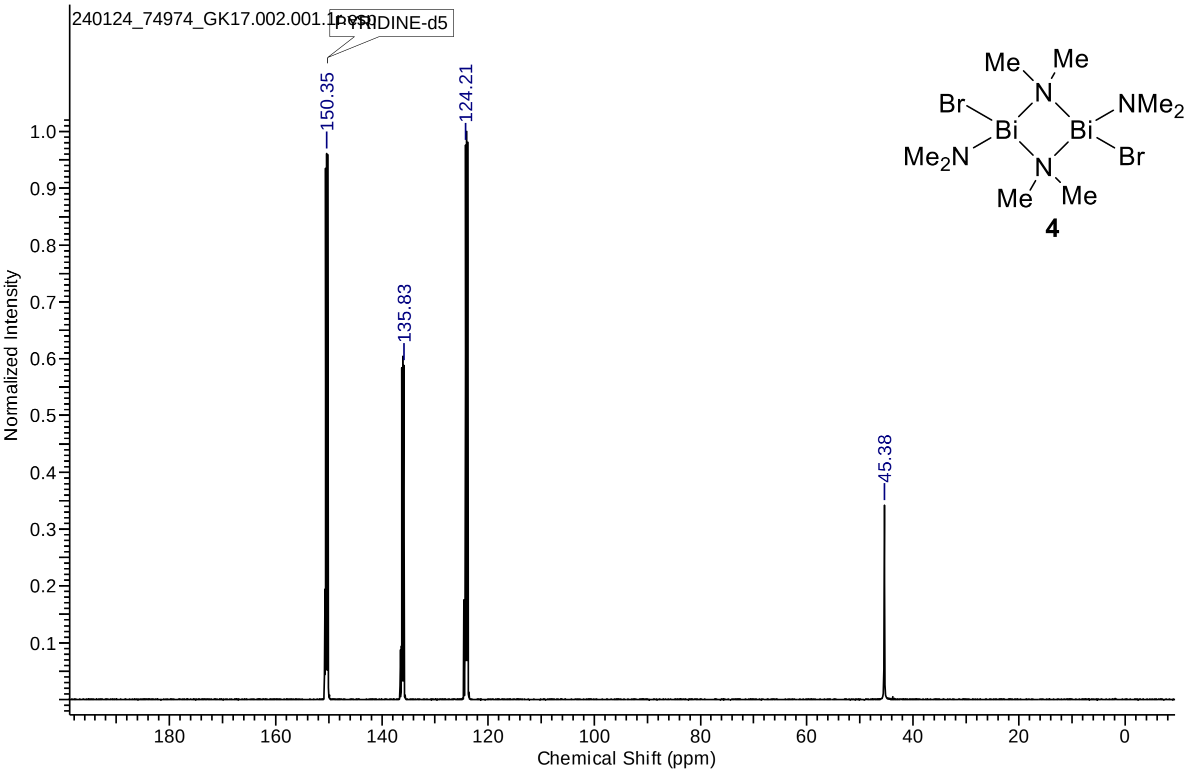

Figure S12. ^{13}C NMR spectrum of **4**.

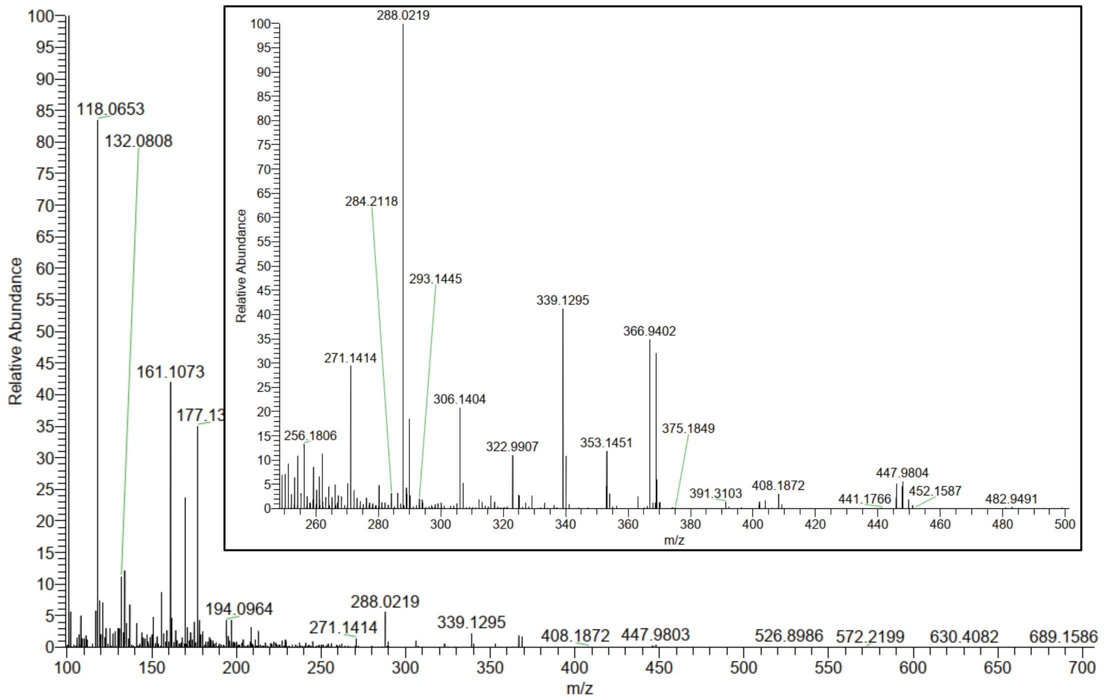

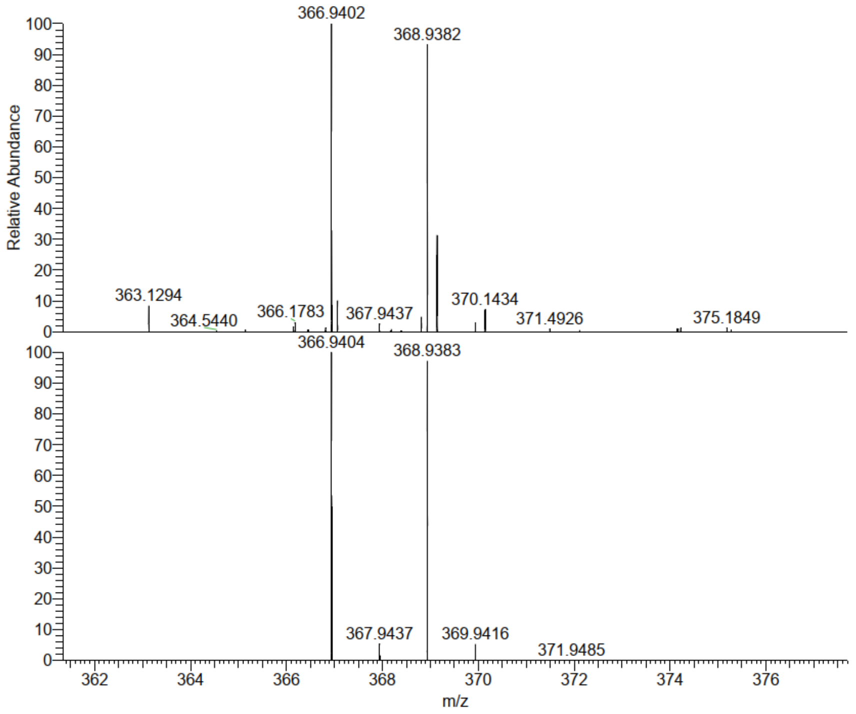

Figure S13. LIFDI-MS spectra of **4**. (top: overview (with inlet: zoom-in), bottom: experimental and simulated spectrum for C_5H_5BiBrN).

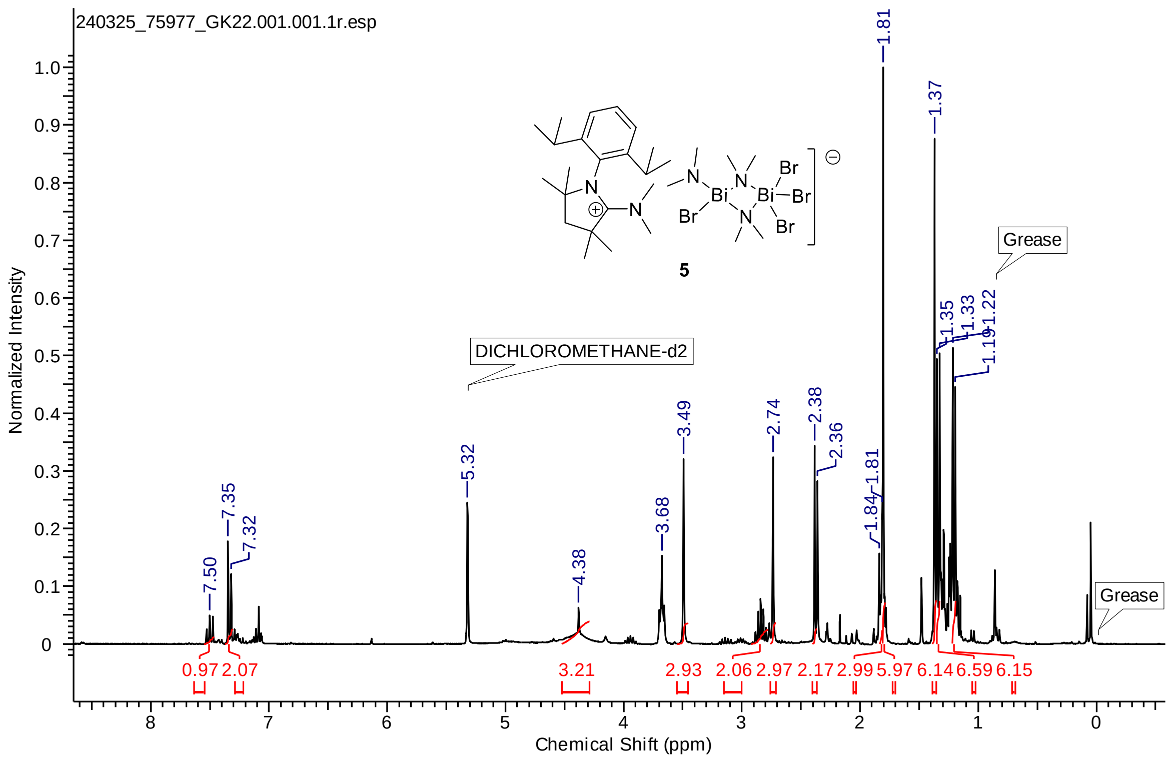

Figure S14. ^{1}H NMR spectrum of **5**.

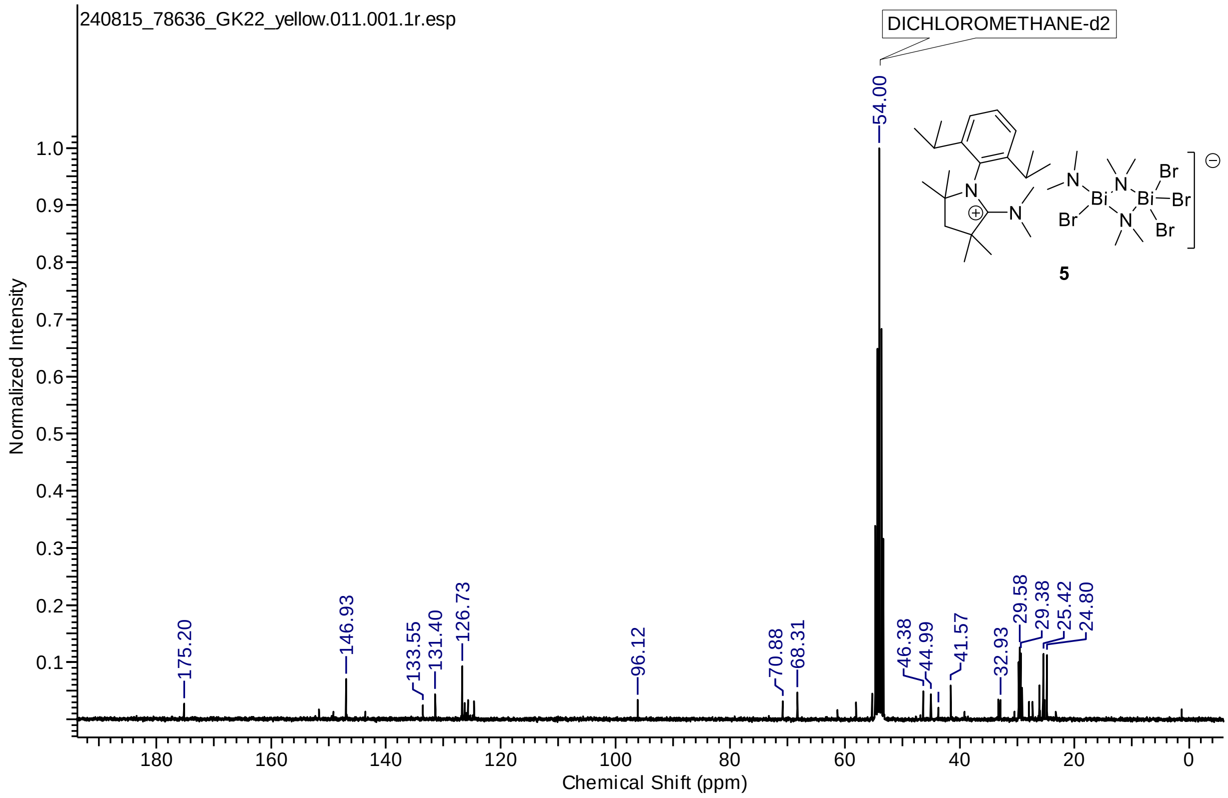

Figure S15. ^{13}C NMR spectrum of **5**.

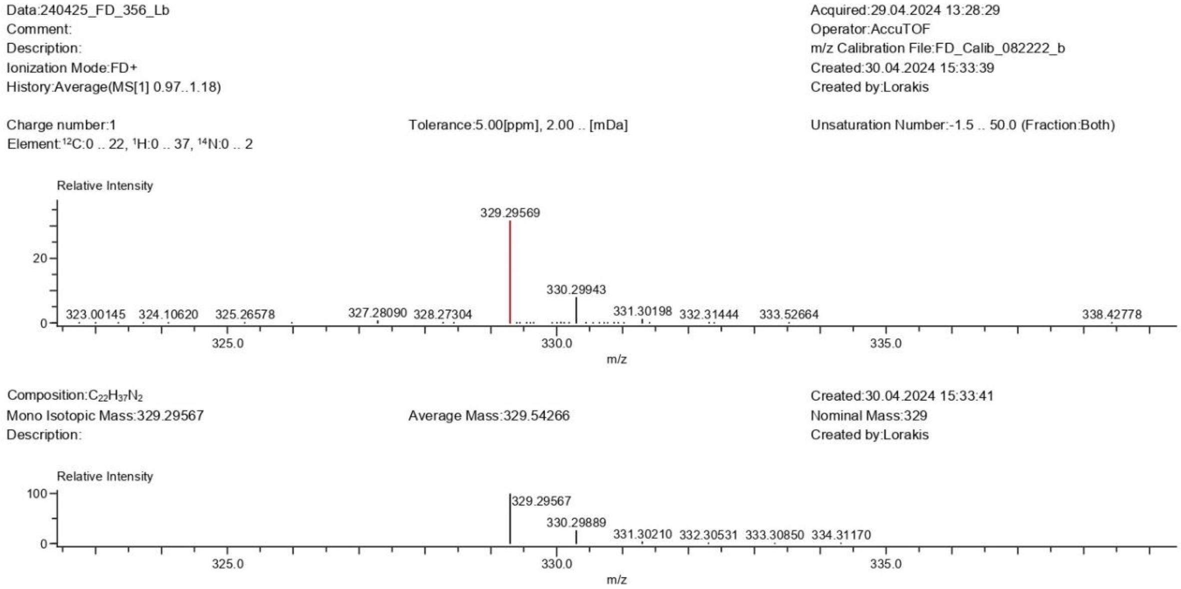

Mass	Intensity	Relative Intensity	Calc. Mass	Mass Difference [mDa]	Mass Difference [ppm]	Possible Formula	Unsaturation Number
329.29569	39065.10	31.39	329.29567	0.02	0.05	$^{12}C_{22}{}^{1}H_{37}{}^{14}N_2$	5.5

Figure S16. HR-MS spectrum of **5**.

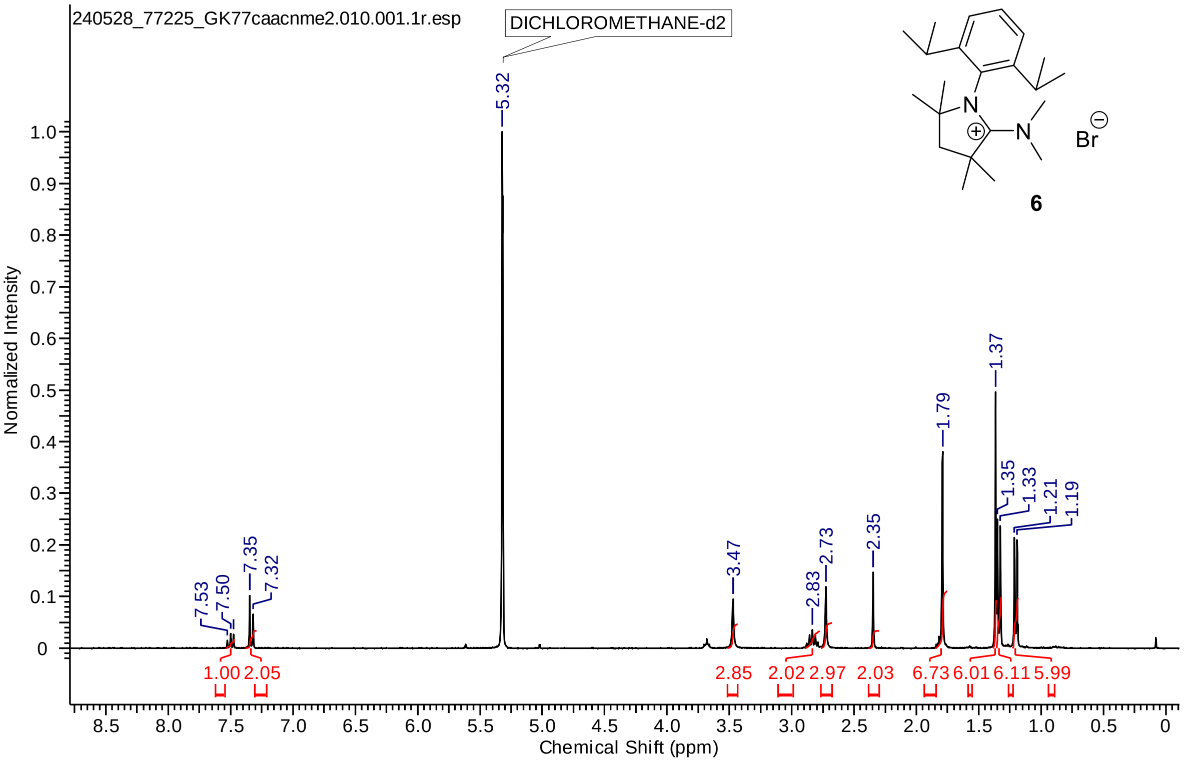

Figure S17. ^{1}H NMR spectrum of **6**.

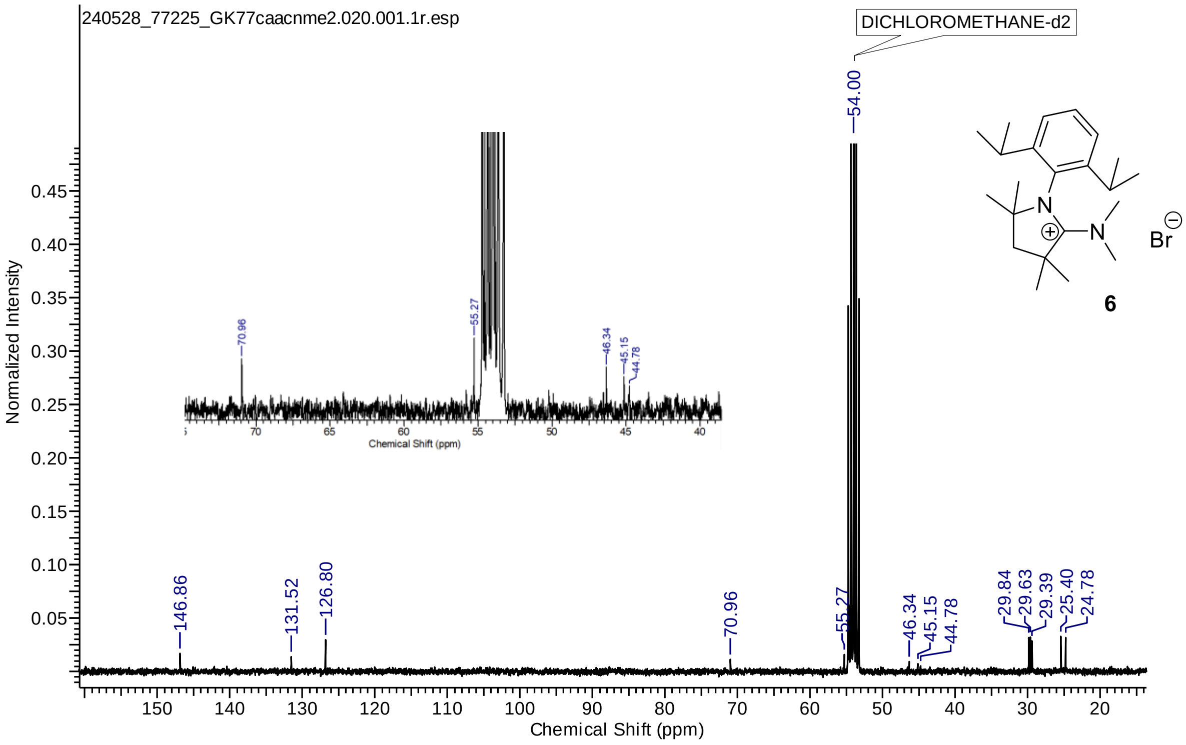

Figure S18. ^{13}C NMR spectrum of **6**.

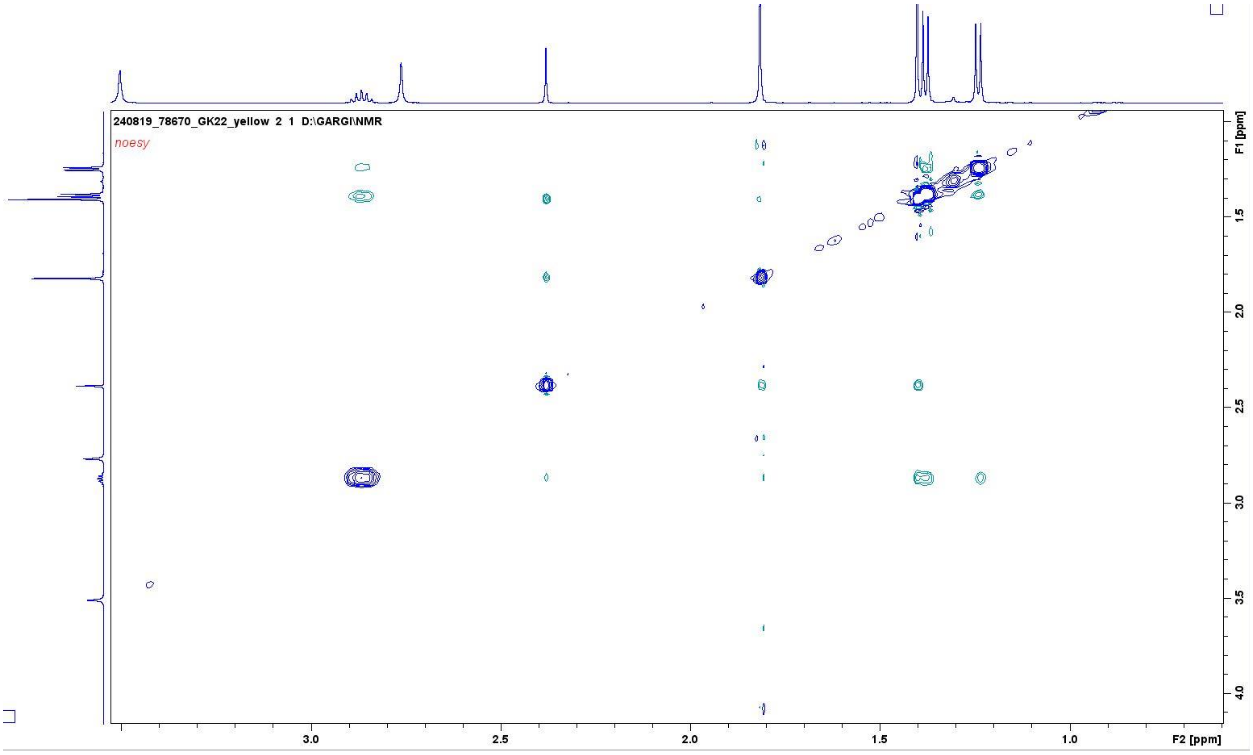

Figure S19. NOESY NMR spectrum of **6**.

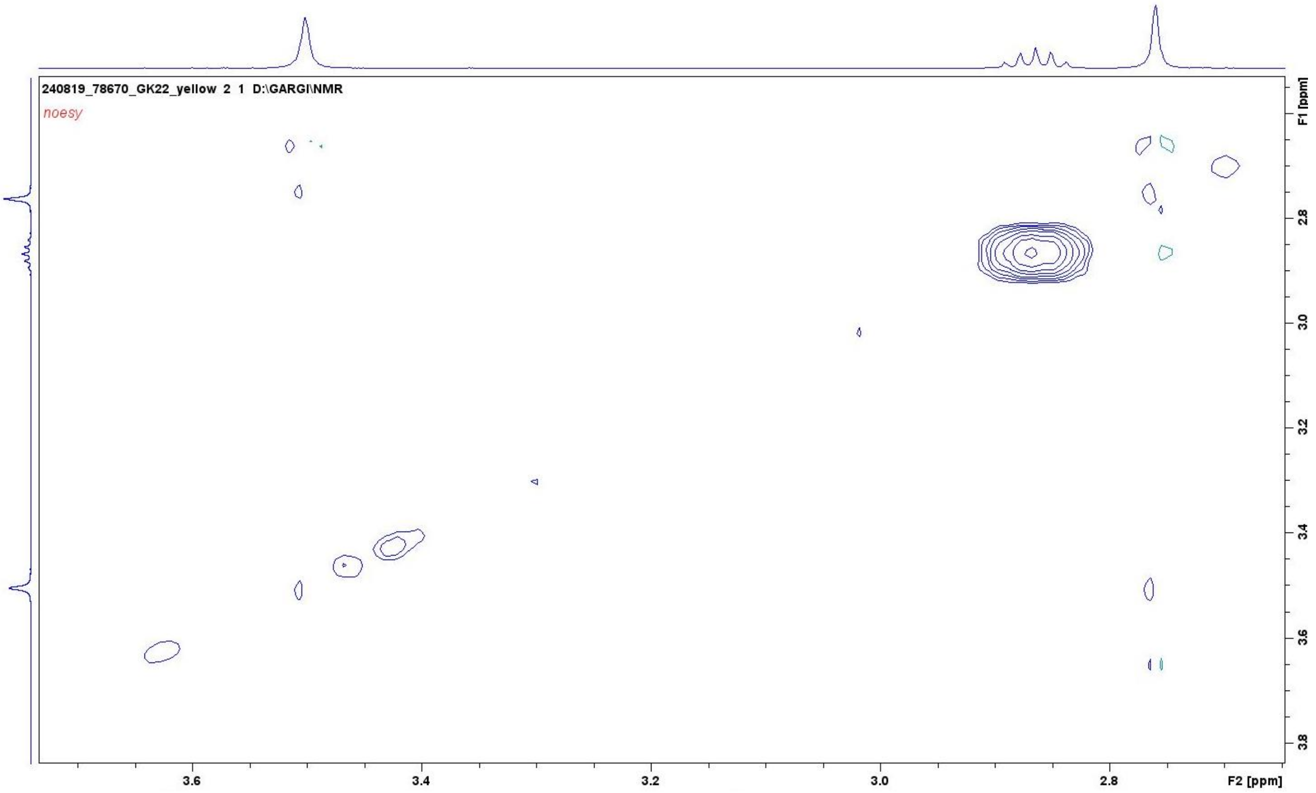

Figure S20. NOESY NMR spectrum of **6**.

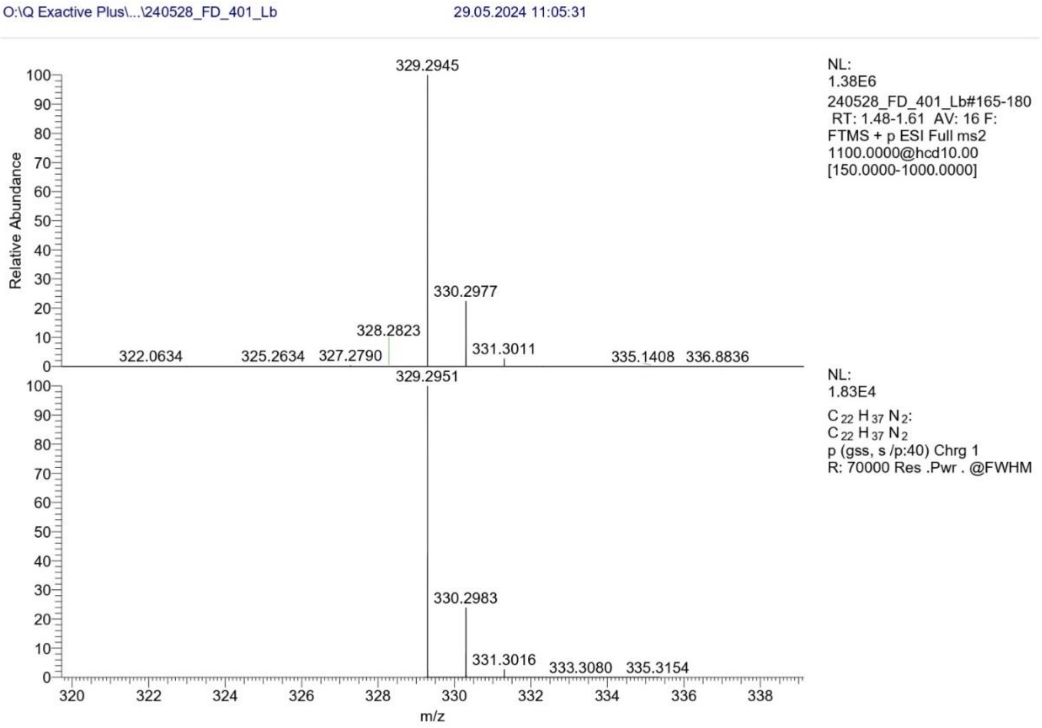

Figure S21. HR-MS spectrum of **6**.

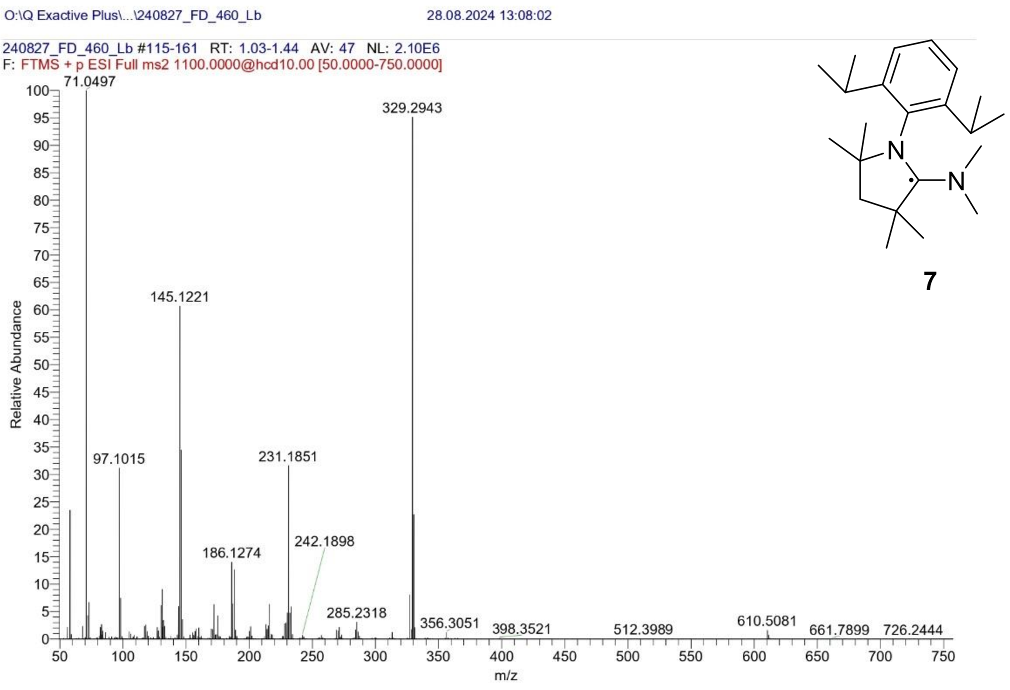

Figure S22. Full HR-MS spectrum of **7**.

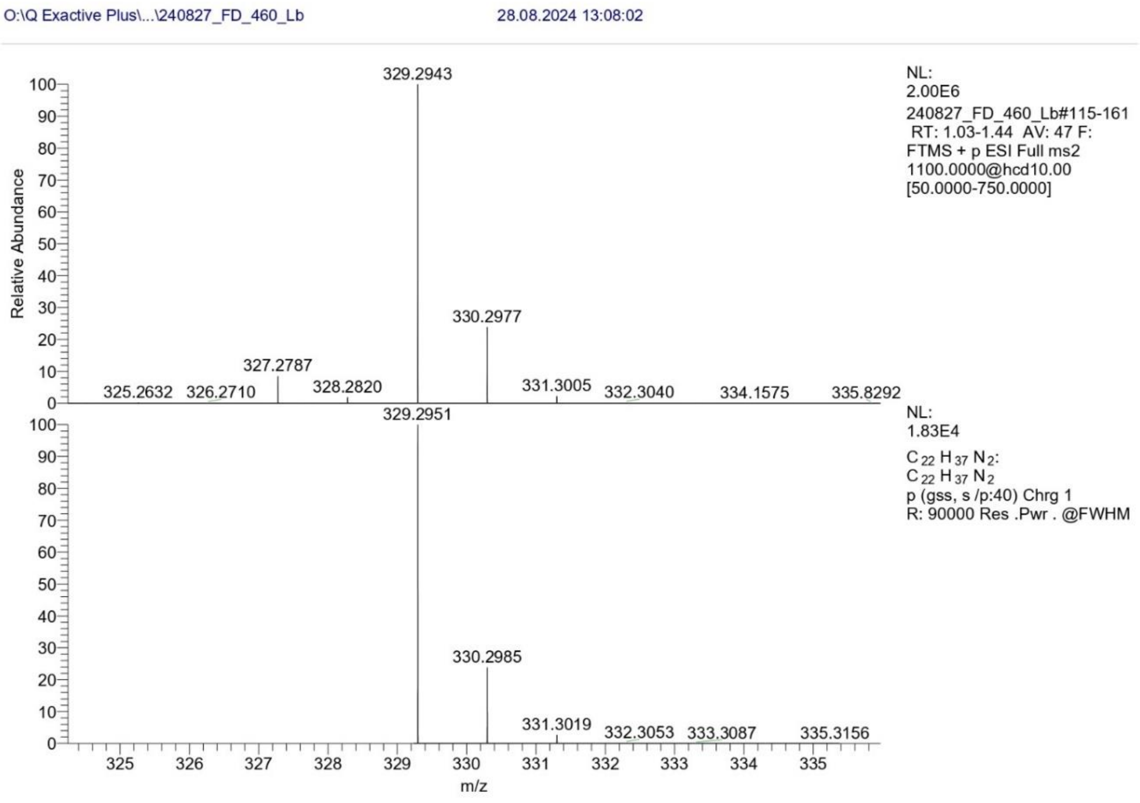

Figure S23. Zoom-in on HR-MS spectrum of **7**.

3. Liquid state UV-Visible spectra of 1 and 3', and 3 and thermochromism of 1

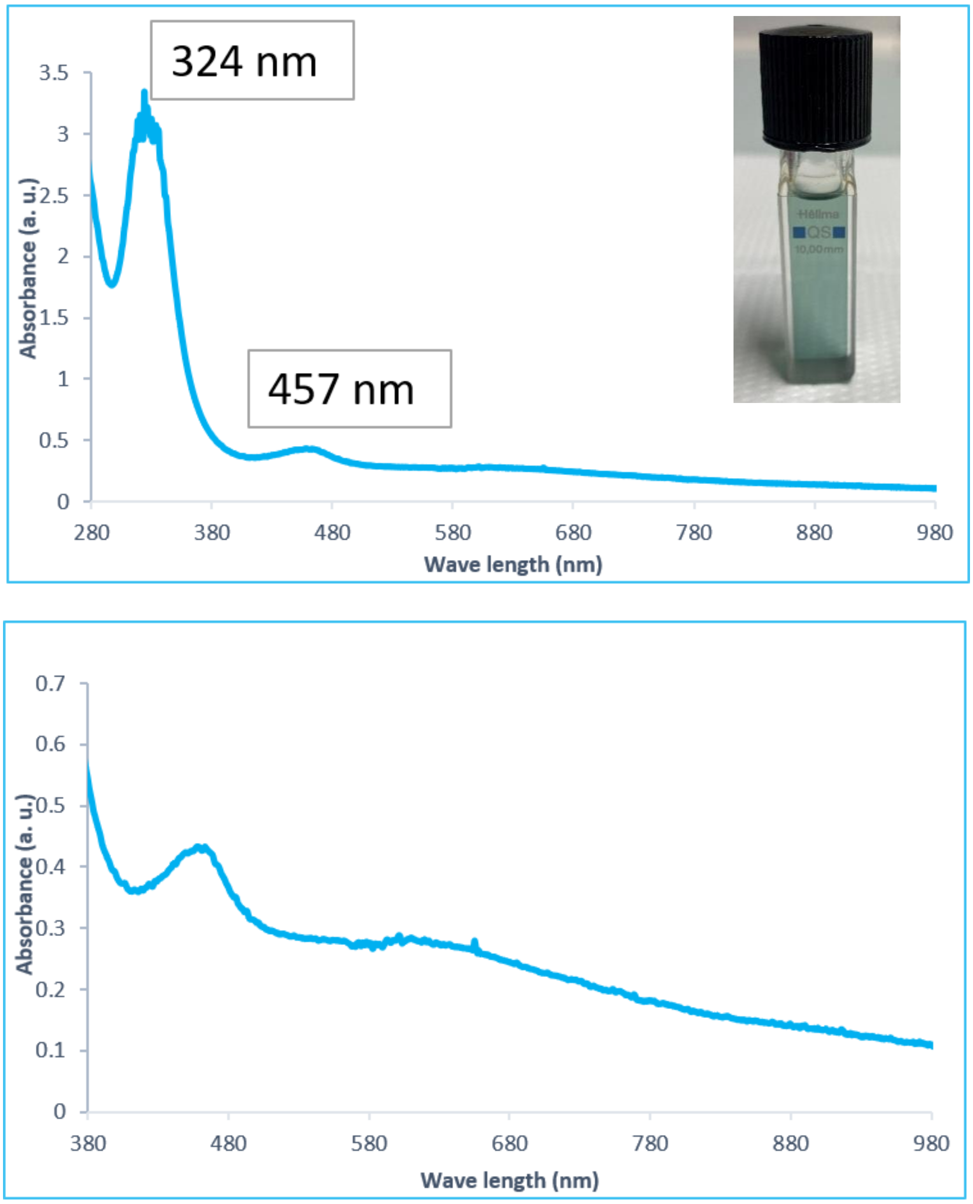

Figure S24. Absorption spectrum of **1** in THF (c =10^{-5} M) at room temperature. Top: full spectrum; bottom: zoom-in on the region corresponding to the wave lengths of visible light. At higher concentrations, the color of the solution appears to show a blue tone.

Analytically pure compound **1** was obtained as a greenish-yellow powder after work-up, when following a previous report, which used NMR spectroscopy as the only means of analysis[14] (Figure S25, left). This compound shows thermochromism: when cooled to –196 °C, it turns colorless (Figure S25, right) and then turns back to pale greenish-yellow color at ambient temperature. Single-crystals of **1** show a green

color and are analytically identical to the powdered sample shown in Figure S25. A much more intense color is observed for concentrated THF solutions of **1**, which show a blue tone.

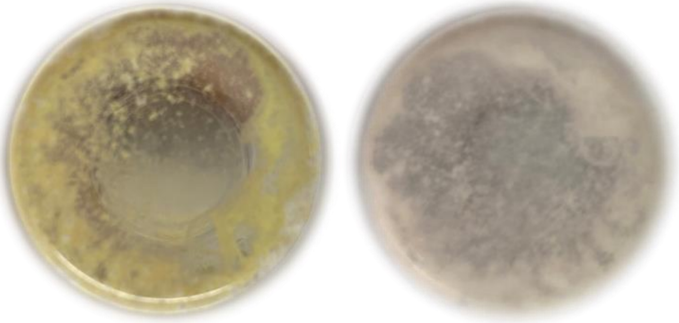

Figure S25. Solid powder sample of **1** at room temperature (left) and at –196 °C (right). The color change is reversible.

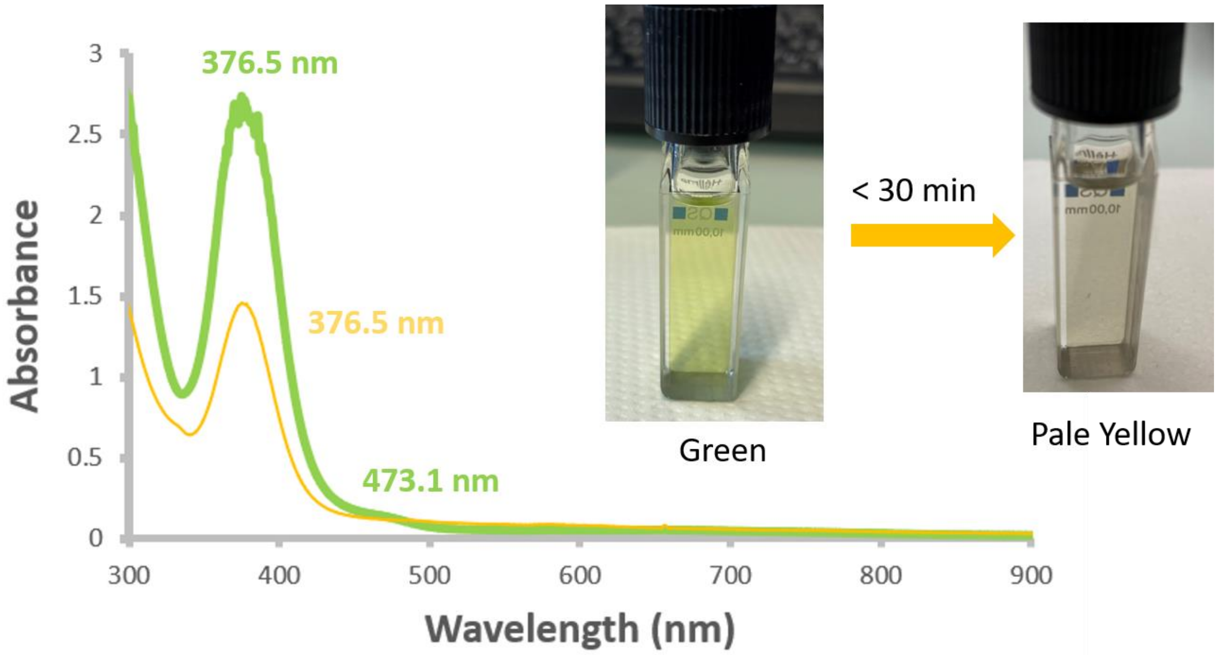

Figure S26. Absorption spectrum of **3'** (spectrum shown in green) and **3** (spectrum shown in yellow) in THF (c =10^{-5} M) at room temperature.

UV-vis spectroscopy of compound 7. The reduction of compound **6** with KC_8 gave a pink oil as a crude product after work-up, which conained compound **7** (shown by EPR spectroscopy), but also diamagnetic side-products (as shown by NMR spectroscopy and magentic susceptibility measurements in solution). UV-vis spectra of the crude product were recorded (pentane solution; absorption maxima at 380, 489, 542, 585 nm). Upon exposure to air, the solutions turn yellow (pentane solution, absorption maximum at 493 nm). However, due to the unknown impurities present in the crude product, these results are not discussed in detail.

4. Cyclic voltammetry of 6

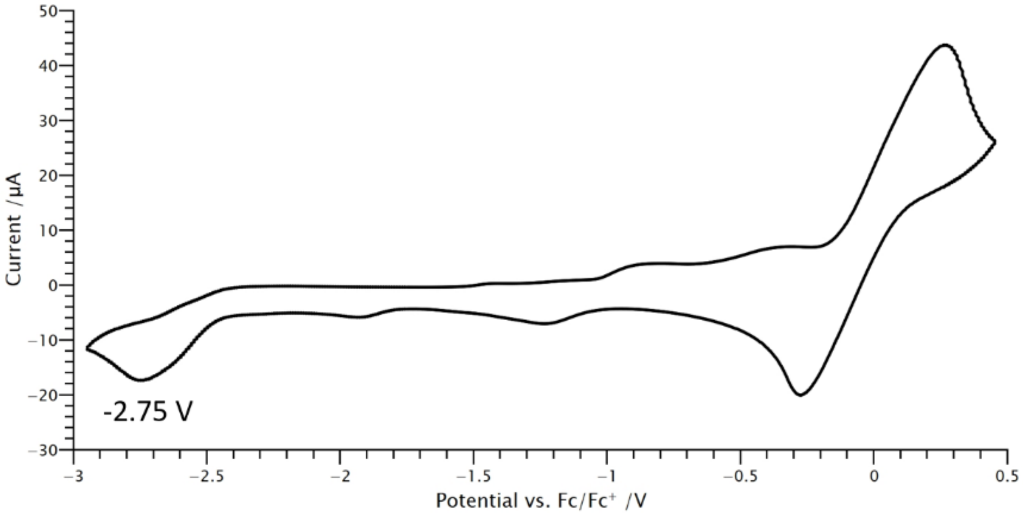

Figure S27. Cyclic voltammogram of **6** in THF with 0.1 M Bu_4NPF_6 as the supporting electrolyte, Ag/AgCl as the reference electrode, and a Pt wire as the counter electrode at 100 mV/S, potentials are reported vs Fc^+/Fc. The cyclic voltammogram shown above contains ferrocene/ferrocenium as a reference.

5. EPR spectroscopy

EPR spectroscopic experiments were performed with an MS5000 X-band CW EPR spectrometer. All experiments were carried out at room temperature under inert conditions. Simulations of the EPR spectra were performed with the EasySpin software package,[60] running in the MATLAB software environment (R2022a; 9.12.0.1927505) provided by MathWorks.

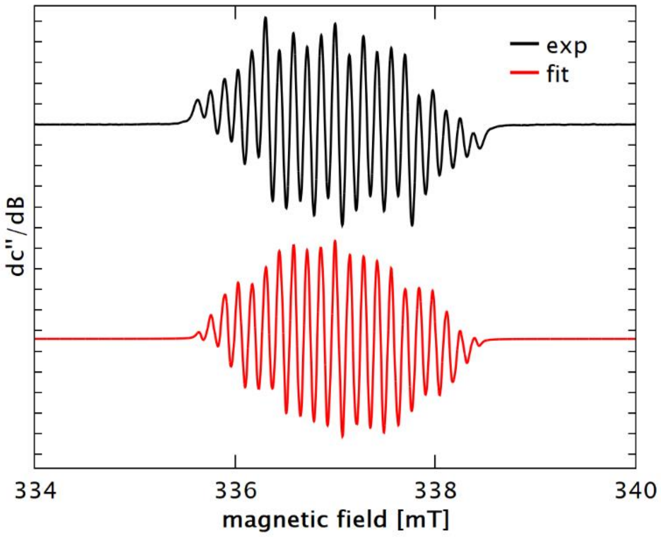

Figure S28. Experimental (black) and simulated (red) continuous-wave (CW) X-band EPR spectra of a solution containing 1 equiv. $BiBr(NMe_2)_2$ ($c = 7.0 \cdot 10^{-2}$ mol/L) and 1 equiv. ^{Me2}CAAC in THF. The observed resonance shows coupling constants of a(1 × ^{14}N) = 15.8 MHz (5.63 G, 0.563 mT), a(1 × ^{14}N) = 11.3 MHz (4.03 G, 0.403 mT), a(3 × ^{1}H) = 4.29 MHz (1.53 G, 0.153 mT) a(3 × ^{1}H) = 3.23 MHz (1.15 G, 0.115 mT) and a g_{iso} value of 2.0026. Spectrometer settings: microwave frequency = 9.447836 GHz, 0.02 mT modulation amplitude at 100 kHz, microwave power = 1 mW, number of accumulated scans = 1, conversion time = 2 ms.

The parameters found for compound **7** are in agreement with those reported for structurally related α-phosphanyl radicals,[54] and show the same trends as those in loosely related oxime ether radical cations stabilized by NHCs.[52] Nevertheless it should be mentioned that fitting parameters with smaller coupling constants for two magnetically inequivalent nitrogen atoms and larger coupling constants for four magnetically inequivalent methyl groups gave results, which were only slightly less satisfactory than those presented above.

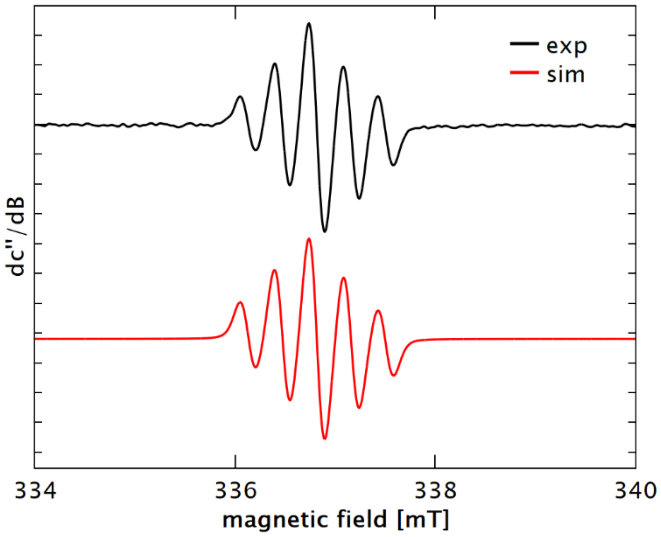

Figure S29. Experimental (black) and simulated (red) continuous-wave (CW) X-band EPR spectra of a solution containing 1 equiv. $BiBr_3$ ($c = 7.1 \cdot 10^{-2}$ mol/L) and 1 equiv. ^{Me2}CAAC in THF. The observed resonance shows coupling constants of $a(2 \times {}^{14}N) = 9.58$ MHz (3.42 G, 0.342 mT) and a g_{iso} value of 2.0039. Spectrometer settings: microwave frequency = 9.447628 GHz, 0.02 mT modulation amplitude at 100 kHz, microwave power = 10 mW, number of accumulated scans = 1, conversion time = 1 ms.

6. Bond lengths and angles of compounds 1-6 as determined by single-crystal XRD

Table S1: Bond Lengths for **1**

Atom	Atom	Length/Å		Atom	Atom	Length/Å
Bi1	Cl1	2.4662(5)		C5	C6	1.382(3)
Bi1	Cl2	2.5951(5)		C6	C7	1.386(3)
Bi1	Cl3	2.7621(5)		C7	C8	1.530(3)
Bi1	C1	2.394(2)		C8	C9	1.536(3)
N1	C1	1.295(3)		C8	C10	1.538(3)
N1	C2	1.466(3)		C11	C12	1.532(3)
N1	C14	1.547(3)		C11	C13	1.537(3)
C1	C18	1.513(3)		C14	C15	1.513(3)
C2	C3	1.402(3)		C14	C16	1.527(3)
C2	C7	1.410(3)		C14	C17	1.532(3)
C3	C4	1.394(3)		C17	C18	1.561(3)
C3	C11	1.521(3)		C18	C19	1.529(3)
C4	C5	1.380(3)		C18	C20	1.539(3)

Table S2: Bond Angles for **1**

Atom	Atom	Atom	Angle/°		Atom	Atom	Atom	Angle/°
Cl1	Bi1	Cl2	92.501(18)		C6	C7	C2	117.2(2)
Cl1	Bi1	Cl3	95.200(18)		C6	C7	C8	118.65(19)
Cl2	Bi1	Cl3	172.096(17)		C7	C8	C9	111.60(18)
C1	Bi1	Cl1	92.74(5)		C7	C8	C10	111.15(19)
C1	Bi1	Cl2	99.31(5)		C9	C8	C10	109.47(19)
C1	Bi1	Cl3	78.58(5)		C3	C11	C12	112.3(2)
C1	N1	C2	122.93(17)		C3	C11	C13	110.24(19)
C1	N1	C14	112.71(17)		C12	C11	C13	108.98(19)
C2	N1	C14	124.17(17)		C15	C14	N1	112.74(18)
N1	C1	Bi1	116.48(14)		C15	C14	C16	110.23(19)
N1	C1	C18	111.04(18)		C15	C14	C17	115.64(19)
C18	C1	Bi1	131.48(14)		C16	C14	N1	107.05(17)
C3	C2	N1	121.66(18)		C16	C14	C17	111.1(2)
C3	C2	C7	122.43(19)		C17	C14	N1	99.37(16)
C7	C2	N1	115.86(18)		C14	C17	C18	106.03(17)
C2	C3	C11	124.1(2)		C1	C18	C17	102.90(17)
C4	C3	C2	117.4(2)		C1	C18	C19	116.11(18)
C4	C3	C11	118.49(19)		C1	C18	C20	106.32(17)
C5	C4	C3	121.3(2)		C19	C18	C17	113.02(18)
C4	C5	C6	120.0(2)		C19	C18	C20	108.31(19)
C5	C6	C7	121.6(2)		C20	C18	C17	109.87(18)
C2	C7	C8	124.11(19)					

Table S3: Bond Lengths for **2**

Atom	Atom	Length/Å		Atom	Atom	Length/Å
Bi1	Cl1	2.562(2)		C31	C32	1.352(9)

Bi1	Cl2	2.549(2)		C33	C32	1.380(9)
Bi1	Cl3	2.538(2)		C33	C34	1.380(8)
Bi1	Cl4	2.888(2)		C34	C29	1.416(8)
Bi1	Cl4’	2.916(2)		C34	C35	1.513(9)
Bi1	O2	2.599(5)		C35	C36	1.515(10)
O2	C45	1.441(8)		C35	C37	1.512(10)
O2	C48	1.434(7)		C40	C38	1.516(9)
C46	C45	1.494(9)		C40	C39	1.525(9)
C47	C46	1.510(9)		C41	C42	1.497(10)
C48	C47	1.507(9)		C43	C42	1.498(10)
Bi2	Cl7	2.534(2)		C44	C43	1.499(10)
Bi2	Cl8	2.814(2)		Cl5	C1	1.657(6)
Bi2	Cl8’’	2.901(2)		N2	C1	1.288(7)
Bi2	Cl9	2.516(2)		N2	C4	1.525(7)
Bi2	Cl10	2.581(4)		N2	C17	1.447(7)
Bi2	Cl11	2.641(5)		C2	C1	1.490(8)
Bi2	O1	2.680(5)		C2	C3	1.527(8)
Cl6	Cl11	2.274(12)		C2	C15	1.520(9)
Cl6	C8	1.518(7)		C2	C16	1.537(9)
O1	C41	1.454(7)		C3	C4	1.522(8)
O1	C44	1.436(7)		C4	C13	1.503(9)
N1	C5	1.539(8)		C14	C4	1.511(8)
N1	C8	1.280(8)		C17	C18	1.398(9)
N1	C29	1.450(7)		C17	C22	1.391(9)
C5	C6	1.540(9)		C18	C19	1.379(9)
C5	C9	1.497(8)		C18	C27	1.510(10)
C5	C10	1.487(9)		C20	C19	1.378(10)
C6	C7	1.533(10)		C20	C21	1.358(10)
C7	C11	1.516(9)		C22	C21	1.404(9)
C7	C12	1.518(8)		C25	C22	1.509(9)
C8	C7	1.496(8)		C25	C23	1.529(9)
C30	C29	1.386(8)		C25	C24	1.528(10)
C30	C40	1.525(8)		C27	C26	1.532(9)
C31	C30	1.387(8)		C27	C28	1.521(9)

Table S4: Bond Angles for **2**.								
**Atom**	**Atom**	**Atom**	**Angle/°**		**Atom**	**Atom**	**Atom**	**Angle/°**
Cl1	Bi1	Cl4	173.57(5)		C12	C7	C6	114.6(6)
Cl1	Bi1	Cl4’	92.61(6)		N1	C8	Cl6	122.9(5)
Cl1	Bi1	O2	84.50(11)		N1	C8	C7	114.4(6)
Cl2	Bi1	Cl1	92.76(6)		C7	C8	Cl6	122.7(5)
Cl2	Bi1	Cl4	90.47(6)		C30	C29	N1	120.5(5)
Cl2	Bi1	Cl4’	98.09(7)		C30	C29	C34	122.8(5)
Cl2	Bi1	O2	176.25(11)		C34	C29	N1	116.7(5)
Cl3	Bi1	Cl1	92.86(7)		C29	C30	C31	116.6(5)
Cl3	Bi1	Cl2	92.10(7)		C29	C30	C40	123.4(5)
Cl3	Bi1	Cl4^{1}	168.18(5)		C31	C30	C40	119.7(5)
Cl3	Bi1	Cl4	92.57(6)		C32	C31	C30	122.1(6)

Cl3	Bi1	O2	85.49(11)		C31	C32	C33	120.6(6)
Cl4	Bi1	Cl4’	81.43(5)		C34	C33	C32	120.9(6)
O2	Bi1	Cl4’	84.61(11)		C29	C34	C35	123.1(6)
O2	Bi1	Cl4	92.50(11)		C33	C34	C29	116.8(6)
Bi1	Cl4	Bi1’	98.56(5)		C33	C34	C35	119.8(6)
C45	O2	Bi1	121.0(4)		C34	C35	C36	109.3(6)
C48	O2	Bi1	118.2(4)		C37	C35	C34	111.9(6)
C48	O2	C45	109.0(5)		C37	C35	C36	111.4(6)
O2	C45	C46	106.1(5)		C38	C40	C30	109.7(5)
C45	C46	C47	103.4(6)		C38	C40	C39	110.7(6)
C48	C47	C46	100.8(5)		C39	C40	C30	112.3(5)
O2	C48	C47	105.8(5)		O1	C41	C42	106.4(5)
Cl7	Bi2	Cl8’’	170.62(7)		C41	C42	C43	106.1(6)
Cl7	Bi2	Cl8	90.64(6)		C42	C43	C44	103.6(6)
Cl7	Bi2	Cl10	101.5(2)		O1	C44	C43	104.7(5)
Cl7	Bi2	Cl11	88.8(2)		C1	N2	C4	111.0(5)
Cl7	Bi2	O1	87.84(11)		C1	N2	C17	125.6(5)
Cl8	Bi2	Cl8’’	81.73(5)		C17	N2	C4	123.3(5)
Cl9	Bi2	Cl7	94.13(7)		N2	C1	Cl5	123.0(5)
Cl9	Bi2	Cl8’’	91.58(6)		N2	C1	C2	115.1(5)
Cl9	Bi2	Cl8	91.90(8)		C2	C1	Cl5	121.8(4)
Cl9	Bi2	Cl10	85.1(2)		C1	C2	C3	100.4(5)
Cl9	Bi2	Cl11	97.4(2)		C1	C2	C15	108.7(5)
Cl9	Bi2	O1	175.70(11)		C1	C2	C16	111.0(5)
Cl10	Bi2	Cl8	167.6(2)		C3	C2	C16	112.1(5)
Cl10	Bi2	O1	98.3(2)		C15	C2	C3	114.1(5)
Cl11	Bi2	Cl8	170.7(2)		C15	C2	C16	110.2(5)
Cl11	Bi2	Cl8’’	97.89(19)		C4	C3	C2	108.1(5)
Cl11	Bi2	O1	86.4(2)		C3	C4	N2	101.2(4)
O1	Bi2	Cl8’’	85.98(10)		C13	C4	N2	108.1(5)
O1	Bi2	Cl8	84.25(11)		C13	C4	C3	113.8(5)
C8	Cl6	Cl11	150.8(3)		C13	C4	C14	109.9(5)
Bi2	Cl8	Bi2’’	98.27(5)		C14	C4	N2	111.0(5)
Cl6	Cl11	Bi2	124.3(4)		C14	C4	C3	112.6(5)
C41	O1	Bi2	123.3(4)		C18	C17	N2	116.8(6)
C44	O1	Bi2	116.0(4)		C22	C17	N2	119.2(5)
C44	O1	C41	106.3(5)		C22	C17	C18	124.0(6)
C8	N1	C5	113.0(5)		C17	C18	C27	123.1(6)
C8	N1	C29	123.1(5)		C19	C18	C17	116.1(6)
C29	N1	C5	124.0(5)		C19	C18	C27	120.5(6)
N1	C5	C6	99.8(5)		C20	C19	C18	122.0(7)
C9	C5	N1	107.6(5)		C21	C20	C19	120.0(6)
C9	C5	C6	112.1(5)		C20	C21	C22	121.8(7)
C10	C5	N1	111.1(5)		C17	C22	C21	115.7(6)
C10	C5	C6	113.5(5)		C17	C22	C25	125.1(6)
C10	C5	C9	111.9(6)		C21	C22	C25	119.0(6)
C7	C6	C5	108.8(5)		C22	C25	C23	109.8(5)
C8	C7	C6	101.3(5)		C22	C25	C24	113.8(6)
C8	C7	C11	108.5(5)		C24	C25	C23	109.9(6)

C8	C7	C12	107.5(5)		C18	C27	C26	112.5(6)
C11	C7	C6	113.3(6)		C18	C27	C28	109.6(6)
C11	C7	C12	110.8(6)		C28	C27	C26	110.3(6)

Table S5: Bond Lengths for **3**						
**Atom**	**Atom**	**Length/Å**		**Atom**	**Atom**	**Length/Å**
Bi1	Br1	2.6966(3)		C5	C6	1.536(4)
Bi1	Br2	2.7301(3)		C6	C21	1.530(4)
Bi1	Br3	2.7571(3)		C7	C12	1.399(3)
Bi1	Br4	3.0191(3)		C8	C9	1.538(4)
Bi1	Br5	3.0522(3)		C8	C13	1.544(4)
Bi1	O1	2.5455(19)		C8	C17	1.530(4)
Br6	C4	1.851(2)		C10	C11	1.537(4)
O1	C1	1.455(3)		C11	C12	1.520(4)
O1	C25	1.457(3)		C11	C22	1.536(4)
C2	C6	1.520(4)		C12	C20	1.404(3)
C2	C7	1.407(3)		C13	C14	1.524(3)
C2	C18	1.398(3)		C14	C15	1.513(4)
N3	C4	1.282(3)		C14	C16	1.521(4)
N3	C7	1.462(3)		C18	C19	1.387(4)
N3	C14	1.545(3)		C19	C20	1.378(4)
C4	C8	1.502(3)		C23	C28	1.454(7)
C1	C26	1.514(4)		C23	$C24^1$	1.428(7)
C26	C3	1.515(4)		C23	C24	1.969(8)
C3	C25	1.513(4)		C28	C24	1.367(8)

Table S6: Bond Angles for **3**.								
**Atom**	**Atom**	**Atom**	**Angle/°**		**Atom**	**Atom**	**Atom**	**Angle/°**
Br1	Bi1	Br2	96.321(10)		C2	C6	C21	112.8(2)
Br1	Bi1	Br3	92.470(9)		C21	C6	C5	109.0(2)
Br1	Bi1	Br4	90.872(10)		C2	C7	N3	118.8(2)
Br1	Bi1	Br5	170.406(7)		C12	C7	C2	123.6(2)
Br2	Bi1	Br3	92.991(8)		C12	C7	N3	117.6(2)
Br2	Bi1	Br4	168.993(8)		C4	C8	C9	108.9(2)
Br2	Bi1	Br5	87.750(9)		C4	C8	C13	100.32(19)
Br3	Bi1	Br4	95.018(7)		C4	C8	C17	110.9(2)
Br3	Bi1	Br5	95.998(7)		C9	C8	C13	113.7(2)
Br4	Bi1	Br5	83.918(9)		C17	C8	C9	110.3(2)
O1	Bi1	Br1	86.84(5)		C17	C8	C13	112.2(2)
O1	Bi1	Br2	88.53(5)		C12	C11	C10	112.5(2)
O1	Bi1	Br3	178.40(5)		C12	C11	C22	110.1(2)
O1	Bi1	Br4	83.55(5)		C22	C11	C10	109.9(2)
O1	Bi1	Br5	84.58(5)		C7	C12	C11	124.6(2)
Bi1	Br4	Bi1’	96.776(12)		C7	C12	C20	116.6(2)
Bi1	Br5	Bi1’	95.388(11)		C20	C12	C11	118.7(2)
C1	O1	Bi1	123.40(16)		C14	C13	C8	108.7(2)

C1	O1	C25	109.5(2)		C13	C14	N3	100.84(18)
C25	O1	Bi1	120.85(17)		C15	C14	N3	110.9(2)
C7	C2	C6	124.6(2)		C15	C14	C13	113.3(2)
C18	C2	C6	118.6(2)		C15	C14	C16	109.7(2)
C18	C2	C7	116.7(2)		C16	C14	N3	108.7(2)
C4	N3	C7	125.5(2)		C16	C14	C13	113.0(2)
C4	N3	C14	111.43(19)		C19	C18	C2	121.1(2)
C7	N3	C14	122.98(18)		C20	C19	C18	120.6(2)
N3	C4	Br6	123.03(18)		C19	C20	C12	121.3(2)
N3	C4	C8	115.4(2)		C28	C23	C24	44.0(3)
C8	C4	Br6	121.45(17)		C24’’	C23	C28	120.7(5)
O1	C1	C26	105.0(2)		C24’’	C23	C24	91.3(4)
C1	C26	C3	102.1(2)		C24	C28	C23	88.5(6)
C25	C3	C26	102.9(2)		C23’’	C24	C23	88.7(4)
O1	C25	C3	105.4(2)		C28	C24	C23	47.6(4)
C2	C6	C5	110.1(2)		C28	C24	C23’’	120.6(6)

Table S7: Bond Lengths for **4**

**Atom**	**Atom**	**Length/Å**		**Atom**	**Atom**	**Length/Å**
Bi1	Bi2	3.549(2)		N4	C7	1.470(9)
Bi1	Br2	2.9489(13)		N4	C8	1.452(10)
Bi1	N3	2.130(6)		N3	C2	1.461(9)
Bi1	N1	2.412(6)		N3	C1	1.467(9)
Bi1	N2	2.261(6)		N1	C4	1.467(9)
Bi2	Br1	2.8347(12)		N1	C3	1.477(9)
Bi2	N4	2.166(6)		N2	C5	1.468(9)
Bi2	N1	2.307(6)		N2	C6	1.465(9)
Bi2	N2	2.514(6)				

Table S8: Bond Angles for **4**

**Atom**	**Atom**	**Atom**	**Angle/˚**		**Atom**	**Atom**	**Atom**	**Angle/˚**
Br2	Bi1	Bi2	129.78(3)		N2	Bi2	Br1	166.26(13)
N3	Bi1	Bi2	107.30(16)		C7	N4	Bi2	112.2(5)
N3	Bi1	Br2	93.23(16)		C8	N4	Bi2	117.5(5)
N3	Bi1	N1	87.6(2)		C8	N4	C7	108.6(6)
N3	Bi1	N2	92.9(2)		C2	N3	Bi1	118.6(4)
N1	Bi1	Bi2	40.14(13)		C2	N3	C1	109.7(6)
N1	Bi1	Br2	168.99(13)		C1	N3	Bi1	115.2(5)
N2	Bi1	Bi2	44.81(14)		Bi2	N1	Bi1	97.5(2)
N2	Bi1	Br2	90.12(15)		C4	N1	Bi1	121.6(4)
N2	Bi1	N1	78.88(19)		C4	N1	Bi2	103.0(4)
Br1	Bi2	Bi1	128.82(3)		C4	N1	C3	108.3(6)
N4	Bi2	Bi1	73.87(16)		C3	N1	Bi1	107.3(4)
N4	Bi2	Br1	96.22(16)		C3	N1	Bi2	119.8(4)
N4	Bi2	N1	93.8(2)		Bi1	N2	Bi2	95.9(2)

N4	Bi2	N2	86.7(2)		C5	N2	Bi1	114.1(4)
N1	Bi2	Bi1	42.36(14)		C5	N2	Bi2	113.0(4)
N1	Bi2	Br1	90.45(14)		C6	N2	Bi1	117.6(4)
N1	Bi2	N2	75.94(19)		C6	N2	Bi2	106.1(4)
N2	Bi2	Bi1	39.34(13)		C6	N2	C5	109.3(6)

Table S9: Bond Lengths for **5**.

**Atom**	**Atom**	**Length/Å**		**Atom**	**Atom**	**Length/Å**
Bi1	Bi2	3.5953(10)		C23	C25	1.50(4)
Bi1	Br4	2.790(3)		C26	C27	1.53(3)
Bi1	N2	2.517(17)		C26	C28	1.52(4)
Bi1	N3	2.258(16)		N4A	C7A	1.49(3)
Bi1	N1	2.190(15)		N4A	C8A	1.47(3)
Bi2	Br1	2.952(2)		N4A	C9A	1.31(2)
Bi2	Br2	2.715(3)		N5A	C9A	1.36(3)
Bi2	Br3	2.862(2)		N5A	C12A	1.53(2)
Bi2	N2	2.309(13)		N5A	C17A	1.48(3)
Bi2	N3	2.488(17)		C9A	C10A	1.54(2)
N2	C3	1.40(3)		C10A	C11A	1.53(3)
N2	C4	1.47(2)		C10A	C13A	1.51(3)
C6	N3	1.46(2)		C10A	C14A	1.54(3)
N3	C5	1.47(2)		C11A	C12A	1.52(3)
C2	N1	1.43(3)		C12A	C15A	1.46(3)
N1	C1	1.44(3)		C12A	C16A	1.55(3)
N4	C7	1.49(3)		C17A	C18A	1.379(17)
N4	C8	1.48(3)		C17A	C22A	1.386(17)
N4	C9	1.31(2)		C18A	C19A	1.378(17)
N5	C9	1.36(3)		C18A	C26A	1.55(3)
N5	C12	1.53(2)		C19A	C20A	1.377(18)
N5	C17	1.48(3)		C20A	C21A	1.378(18)
C9	C10	1.54(2)		C21A	C22A	1.372(17)
C10	C11	1.52(3)		C22A	C23A	1.55(3)
C10	C13	1.50(3)		C23A	C24A	1.51(4)
C10	C14	1.54(3)		C23A	C25A	1.51(4)
C11	C12	1.52(3)		C26A	C27A	1.53(3)
C12	C15	1.46(4)		C26A	C28A	1.52(3)
C12	C16	1.55(3)		O1	C29	1.30(8)
C17	C18	1.385(17)		O1	C32	1.70(8)
C17	C22	1.383(18)		C29	C30	1.68(10)
C18	C19	1.378(17)		C30	C31	1.55(7)
C18	C26	1.55(3)		C31	C32	1.42(9)
C19	C20	1.379(18)		O1A	C29A	1.64(12)
C20	C21	1.379(18)		O1A	C32A	1.22(10)
C21	C22	1.380(17)		C29A	C30A	1.10(13)
C22	C23	1.56(3)		C30A	C31A	1.53(10)
C23	C24	1.51(4)		C31A	C32A	1.63(15)

Table S10: Bond Angles for **5**.								
**Atom**	**Atom**	**Atom**	**Angle/°**		**Atom**	**Atom**	**Atom**	**Angle/°**
Br4	Bi1	Bi2	128.36(7)		C17	C18	C26	123(2)
N2	Bi1	Bi2	39.7(3)		C19	C18	C17	123(3)
N2	Bi1	Br4	164.8(3)		C19	C18	C26	113(2)
N3	Bi1	Bi2	43.2(4)		C18	C19	C20	114(3)
N3	Bi1	Br4	88.3(4)		C19	C20	C21	124(3)
N3	Bi1	N2	77.1(5)		C20	C21	C22	121(3)
N1	Bi1	Bi2	106.9(5)		C17	C22	C23	127(2)
N1	Bi1	Br4	92.6(5)		C21	C22	C17	116(3)
N1	Bi1	N2	84.9(6)		C21	C22	C23	117(2)
N1	Bi1	N3	96.5(7)		C24	C23	C22	112(3)
Br1	Bi2	Bi1	67.04(4)		C25	C23	C22	113(3)
Br2	Bi2	Bi1	108.41(7)		C25	C23	C24	113(3)
Br2	Bi2	Br1	171.97(8)		C27	C26	C18	115(3)
Br2	Bi2	Br3	90.29(8)		C28	C26	C18	112(3)
Br3	Bi2	Bi1	128.88(5)		C28	C26	C27	107(3)
Br3	Bi2	Br1	97.70(6)		C8A	N4A	C7A	110.1(19)
N2	Bi2	Bi1	44.1(4)		C9A	N4A	C7A	124(2)
N2	Bi2	Br1	86.2(4)		C9A	N4A	C8A	125(2)
N2	Bi2	Br2	94.8(4)		C9A	N5A	C12A	110.4(16)
N2	Bi2	Br3	88.5(4)		C9A	N5A	C17A	126.0(19)
N2	Bi2	N3	76.8(5)		C17A	N5A	C12A	119.7(19)
N3	Bi2	Bi1	38.4(4)		N4A	C9A	N5A	120.9(19)
N3	Bi2	Br1	83.6(3)		N4A	C9A	C10A	127(2)
N3	Bi2	Br2	88.8(3)		N5A	C9A	C10A	111.6(16)
N3	Bi2	Br3	165.1(4)		C11A	C10A	C9A	101.2(17)
Bi2	N2	Bi1	96.2(6)		C11A	C10A	C14A	110(2)
C3	N2	Bi1	120.3(13)		C13A	C10A	C9A	115.3(19)
C3	N2	Bi2	111.5(13)		C13A	C10A	C11A	112(2)
C3	N2	C4	109.2(17)		C13A	C10A	C14A	108(2)
C4	N2	Bi1	106.8(12)		C14A	C10A	C9A	111(2)
C4	N2	Bi2	112.3(11)		C12A	C11A	C10A	108.0(17)
Bi1	N3	Bi2	98.4(6)		N5A	C12A	C16A	108.9(19)
C6	N3	Bi1	114.0(11)		C11A	C12A	N5A	102.2(17)
C6	N3	Bi2	105.5(11)		C11A	C12A	C16A	111(2)
C6	N3	C5	109.5(14)		C15A	C12A	N5A	109(2)
C5	N3	Bi1	113.4(13)		C15A	C12A	C11A	115(2)
C5	N3	Bi2	115.6(12)		C15A	C12A	C16A	110(2)
C2	N1	Bi1	113.8(16)		C18A	C17A	N5A	120(2)
C2	N1	C1	111.4(19)		C18A	C17A	C22A	122(2)
C1	N1	Bi1	119.7(15)		C22A	C17A	N5A	117.2(19)
C8	N4	C7	111(3)		C17A	C18A	C26A	125(2)
C9	N4	C7	121(2)		C19A	C18A	C17A	122(3)
C9	N4	C8	123(4)		C19A	C18A	C26A	112(2)
C9	N5	C12	110.0(17)		C20A	C19A	C18A	114(3)
C9	N5	C17	126(2)		C19A	C20A	C21A	124(3)

C17	N5	C12	121(2)		C22A	C21A	C20A	120(3)
N4	C9	N5	123(2)		C17A	C22A	C23A	125(2)
N4	C9	C10	125(2)		C21A	C22A	C17A	116(2)
N5	C9	C10	111.7(17)		C21A	C22A	C23A	118(2)
C11	C10	C9	100.5(18)		C24A	C23A	C22A	113(3)
C11	C10	C14	110(3)		C24A	C23A	C25A	112(3)
C13	C10	C9	115(2)		C25A	C23A	C22A	112(3)
C13	C10	C11	112(3)		C27A	C26A	C18A	114(2)
C13	C10	C14	108(2)		C28A	C26A	C18A	112(3)
C14	C10	C9	111(2)		C28A	C26A	C27A	107(2)
C10	C11	C12	108.6(19)		C29	O1	C32	105(5)
N5	C12	C16	108(2)		O1	C29	C30	106(5)
C11	C12	N5	103.3(18)		C31	C30	C29	106(5)
C11	C12	C16	113(3)		C32	C31	C30	97(5)
C15	C12	N5	112(2)		C31	C32	O1	106(5)
C15	C12	C11	111(3)		C32A	O1A	C29A	119(8)
C15	C12	C16	110(3)		C30A	C29A	O1A	106(9)
C18	C17	N5	119(2)		C29A	C30A	C31A	103(9)
C22	C17	N5	119(2)		C30A	C31A	C32A	112(7)
C22	C17	C18	121(3)		O1A	C32A	C31A	90(8)

Table S11: Bond Lengths for **6**						
**Atom**	**Atom**	**Length/Å**		**Atom**	**Atom**	**Length/Å**
N1	C4	1.4602(16)		C6	C7	1.5377(18)
N1	C5	1.3391(16)		C6	C18	1.5369(18)
N1	C16	1.5256(16)		C6	C22	1.5409(18)
N2	C5	1.3219(16)		C8	C9	1.3945(19)
N2	C20	1.4710(16)		C8	C13	1.5210(18)
N2	C21	1.4680(16)		C9	C10	1.378(2)
C1	C2	1.5411(19)		C10	C11	1.381(2)
C2	C3	1.5239(18)		C13	C14	1.5353(19)
C2	C12	1.5387(19)		C13	C15	1.5363(18)
C3	C4	1.4128(18)		C16	C17	1.5182(18)
C3	C11	1.3966(18)		C16	C18	1.5277(18)
C4	C8	1.4129(18)		C16	C19	1.5306(18)
C5	C6	1.5297(17)				

Table S12: Bond Angles for **6**								
**Atom**	**Atom**	**Atom**	**Angle/°**		**Atom**	**Atom**	**Atom**	**Angle/°**
C4	N1	C16	120.13(10)		C5	C6	C22	112.55(11)
C5	N1	C4	124.95(10)		C7	C6	C22	109.89(11)
C5	N1	C16	111.98(10)		C18	C6	C7	108.66(11)
C5	N2	C20	122.63(11)		C18	C6	C22	111.61(11)
C5	N2	C21	123.66(11)		C4	C8	C13	124.58(12)
C21	N2	C20	113.44(11)		C9	C8	C4	117.92(12)

C3	C2	C1	111.53(12)		C9	C8	C13	117.46(12)
C3	C2	C12	111.42(11)		C10	C9	C8	122.08(13)
C12	C2	C1	108.02(11)		C9	C10	C11	119.02(13)
C4	C3	C2	125.01(12)		C10	C11	C3	122.25(13)
C11	C3	C2	117.34(12)		C8	C13	C14	112.42(11)
C11	C3	C4	117.58(12)		C8	C13	C15	110.68(11)
C3	C4	N1	121.14(11)		C14	C13	C15	109.26(11)
C3	C4	C8	121.03(12)		N1	C16	C18	100.58(10)
C8	C4	N1	117.82(11)		N1	C16	C19	111.50(10)
N1	C5	C6	111.84(11)		C17	C16	N1	112.42(10)
N2	C5	N1	123.82(11)		C17	C16	C18	110.66(11)
N2	C5	C6	124.30(11)		C17	C16	C19	107.85(11)
C5	C6	C7	113.82(10)		C18	C16	C19	113.82(11)
C5	C6	C18	99.93(10)		C16	C18	C6	108.03(10)

7. Crystallographic data for the structural analysis of compounds 1-6

Single-crystals suitable for X-ray diffraction were coated with polyisobutylene or perfluorinated polyether oil, transferred to a nylon loop and then mounted on the goniometer of a diffractometer equipped with a molybdenum (λ = 0.71073Å) X-ray tube. Diffraction data were collected at 100 K (1, 2 3, 4, 6). In Olex2,[61] the structures were solved using intrinsic phasing methods and expanded using Fourier techniques.[62,63] All nonhydrogen atoms were refined anisotropically. Hydrogen atoms were included in structure factors calculations. All hydrogen atoms were assigned to idealized geometric positions. CCDC numbers 2420687-2420692 contain the supplementary crystallographic data for this paper. These data are provided free of charge by the joint Cambridge Crystallographic Data Centre and Fachinformationszentrum Karlsruhe Access Structures service.

Table S13. Crystallographic data of compounds **1-3**.

**Identification code**	**1**	**2**	**3**
**Empirical formula**	$C_{20}H_{28}BiCl_3N$	$C_{48}H_{78}Bi_2Cl_{9.5}N_2O_2$	$C_{27}H_{39}NBr_5BiO$
**Formula weight**	597.76	1469.85	1002.12
**Temperature/K**	100(2)	100(2)	100(2)
**Crystal system**	monoclinic	triclinic	monoclinic
**Space group (number)**	$P2_1/n$ (14)	$P\overline{1}$ (2)	C2/c (15)
**a/Å**	10.3559(5)	9.811(6)	29.5536(18)
**b/Å**	10.2209(4)	16.420(12)	11.0845(8)
**c/Å**	21.9823(9)	19.735(12)	20.5919(15)
**α/°**	90	67.575(16)	90
**β/°**	103.251(2)	86.785(13)	104.316(2)
**γ/°**	90	81.168(19)	90
**Volume/Å^3**	2264.80(17)	2904(3)	6536.2(8)
**Z**	4	2	8
**ρ_{calc} / g·cm^{-3}**	1.753	1.681	2.037
**μ/mm^{-1}**	8.142	6.525	11.528
**F(000)**	1156.0	1447.0	3792.0
**Crystal size/mm^3**	0.339×0.230×0.204	0.131×0.064×0.057	0.21×0.11×0.07
**Radiation**	MoKα (λ = 0.71073 Å)	MoKα (λ = 0.71073 Å)	MoKα (λ = 0.71073 Å)
**2Θ range for data collection/°**	4.416 to 61.238	4.112 to 56.072	4.272 to 61.098
**Index ranges**	–14 ≤ h ≤ 14, –14 ≤ k ≤ 14, –30 ≤ l ≤ 31	–12 ≤ h ≤ 12, –21 ≤ k ≤ 21, –25 ≤ l ≤ 26	–36 ≤ h ≤ 42, –15 ≤ k ≤ 15, –29 ≤ l ≤ 29
**Reflections collected**	93107	77543	102504
**Independent reflections**	6914 [R_{int} = 0.0487, R_{sigma} = 0.0232]	13825 [R_{int} = 0.0898, R_{sigma} = 0.0697]	9979 [R_{int} = 0.0452, R_{sigma} = 0.0254]
**Data/restraints/parameters**	6914/0/234	13825/859/603	9979/0/325
**Goodness-of-fit on F^2**	1.068	1.030	1.021
**Completeness to θ = 25.242°**	99.9%	99.9%	99.9%
**Final R indexes [I>=2σ (I)]**	R_1 = 0.0190, wR_2 = 0.0367	R_1 = 0.0435, wR_2 = 0.0893	R_1 = 0.0225, wR_2 = 0.0435
**Final R indexes [all data]**	R_1 = 0.0255, wR_2 = 0.0378	R_1 = 0.0778, wR_2 = 0.1010	R_1 = 0.0302, wR_2 = 0.0456
**Largest diff. peak/hole / e Å^{-3}**	0.99/–0.73	1.54/–2.60	1.04/–0.94
**CCDC No:**	2420691	2420689	2420692

Table S14. Crystallographic data of compounds **4-6**.

Identification code	4	5	6
**Empirical formula**	$C_8H_{24}Bi_2Br_2N_4$	$C_{32}H_{60.7}N_5Br_4Bi_2O$	$C_{22}H_{37}BrN_2$
**Formula weight**	754.09	1269.15	409.44
**Temperature/K**	100(2)	100(2)	100(2)
**Crystal system**	monoclinic	triclinic	monoclinic
**Space group (number)**	$P2_1/n$ (14)	$P\overline{1}$ (2)	$P\overline{1}$ (2)
**a/Å**	14.960(6)	11.4979(9)	10.3667(3)
**b/Å**	7.831(5)	15.3523(16)	11.4949(3)
**c/Å**	15.981(6)	17.0094(12)	18.4103(5)
**α/°**	90	114.7350(10)	90
**β/°**	117.000(11)	107.1030(10)	90.7220(10)
**γ/°**	90	95.581(2)	90
**Volume/Å^3**	1668.3(14)	2521.1(4)	2193.67(10)
**Z**	4	2	4
**ρ_{calc} / g·cm^{-3}**	3.002	1.672	1.240
**μ/mm^{-1}**	25.844	10.165	1.881
**F(000)**	1344.0	1203.0	872.0
**Crystal size/mm^3**	0.299×0.185×0.165	0.189×0.171×0.089	0.25×0.21×0.18
**Radiation**	MoK_α (λ=0.71073 Å)	MoK_α (λ=0.71073 Å)	MoK_α (λ=0.71073 Å)
**2θ range [°]**	5.046 to 50.048	4.65 to 50.044	4.178 to 56.654
**Index ranges**	$-17 \leq h \leq 17$ $-9 \leq k \leq 9$ $-19 \leq l \leq 19$	$-13 \leq h \leq 13$ $-18 \leq k \leq 18$ $-20 \leq l \leq 20$	$-13 \leq h \leq 13$ $-14 \leq k \leq 15$ $-24 \leq l \leq 24$
**Reflections collected**	40409	30113	89245
**Independent reflections**	2941 [R_{int} = 0.0520, R_{sigma} = 0.0260]	8758 [R_{int} = 0.0722, R_{sigma} = 0.0759]	5460 [R_{int} = 0.0593, R_{sigma} = 0.0213]
**Data/restraints/parameters**	2941/0/147	8758/1641/612	5460/0/236
**Goodness-of-fit on F^2**	1.212	1.049	1.021
**Completeness to θ = 25.242°**	99.9 %	99.9 %	99.9 %
**Final R indexes [I>=2σ (I)]**	R_1 = 0.0307, wR_2 = 0.0809	R_1 = 0.0798, wR_2 = 0.1928	R_1 = 0.0230, wR_2 = 0.0533
**Final R indexes [all data]**	R_1 = 0.0309, wR_2 = 0.0811	R_1 = 0.1148, wR_2 = 0.2196	R_1 = 0.0300, wR_2 = 0.0557
**Largest diff. peak/hole / e Å^{-3}**	3.38/−3.06	4.61/−3.11	0.45/−0.35
**CCDC No:**	2420687	2420688	2420690

8. Quantum chemical calculations

All calculations were carried out with the Amsterdam Density Functional (ADF) program using dispersion-corrected density functional theory at the ZORA-BLYP-D3(BJ)/TZP level of theory. The effect of solvation in THF was simulated by means of the Conductor like Screening Model (COSMO) of solvation as implemented in ADF. UV-Vis spectra has been computed with TD-DFT at the same level of theory.[64–72]

A concentration correction of $\Delta G^{0\to*}$ = RT·ln(24.46) = 1.894 kcal mol^{-1} (T = 298.15 K) was added to the free energies of all calculated species to change the 1.00 atm gas phase values to the condensed phase standard state concentration of 1.00 mol·L^{-1}. For THF, a standard state of 12.3 mol L^{-1} at 298.15 K was used, whereby its $\Delta G^{0\to*}$ correction is 3.38 kcal mol^{-1}.[73,74]

Table S15. Thermodynamic data for the conversion of **1** and **3‘** into thf-stabilized mononuclear species. Values are given in kcal mol^{-1}.

	ΔE	ΔH	ΔG	ΔG^*
**1** + 2 thf → 2 [$BiCl_3$(CAAC)(thf)]	–4.8	–0.6	1.8	–3.1
**3’** + 2 thf → 2 [$BiBr_3$(CAAC)(thf)]	–2.9	2.0	2.4	–2.4

ΔG^* refers to concentration-corrected values reflecting the concentration of 1.00 mol·L^{-1}.

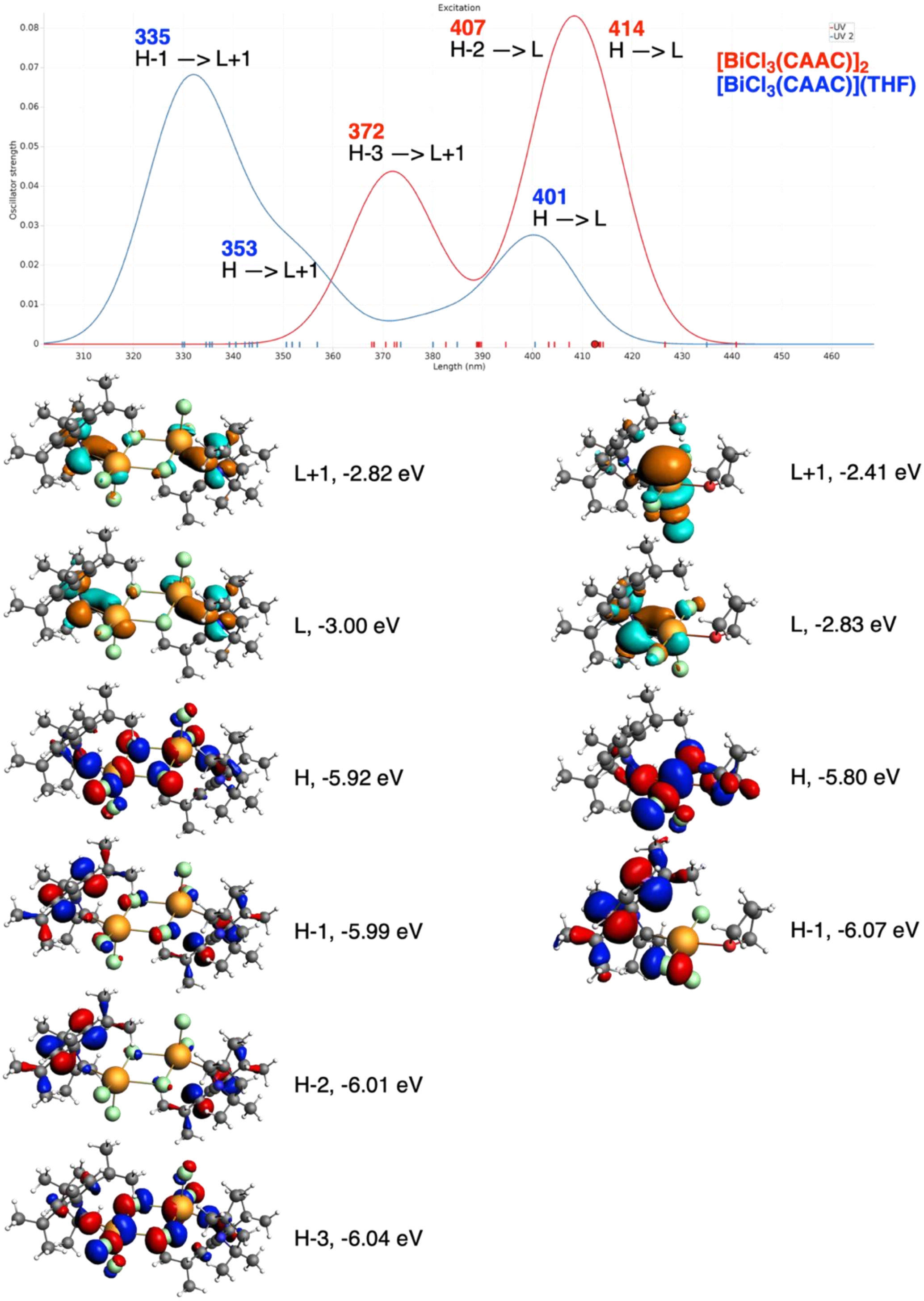

Figure S30. Calculated UV/vis spectra of $[BiCl_3(CAAC)](thf)$ and $[BiCl_3(CAAC)]_2$ (**1**), together with the molecular orbitals involved in the main excitations (H = HOMO, L = LUMO). $[BiCl_3(CAAC)(thf)]$ shows an additional calculated band of weak intensity at 435 nm. The red-shift of the absorption maximum for $[BiCl_3(CAAC)]_2$ is in agreement with visual experimental observations (see Figure cation of Figure S24).

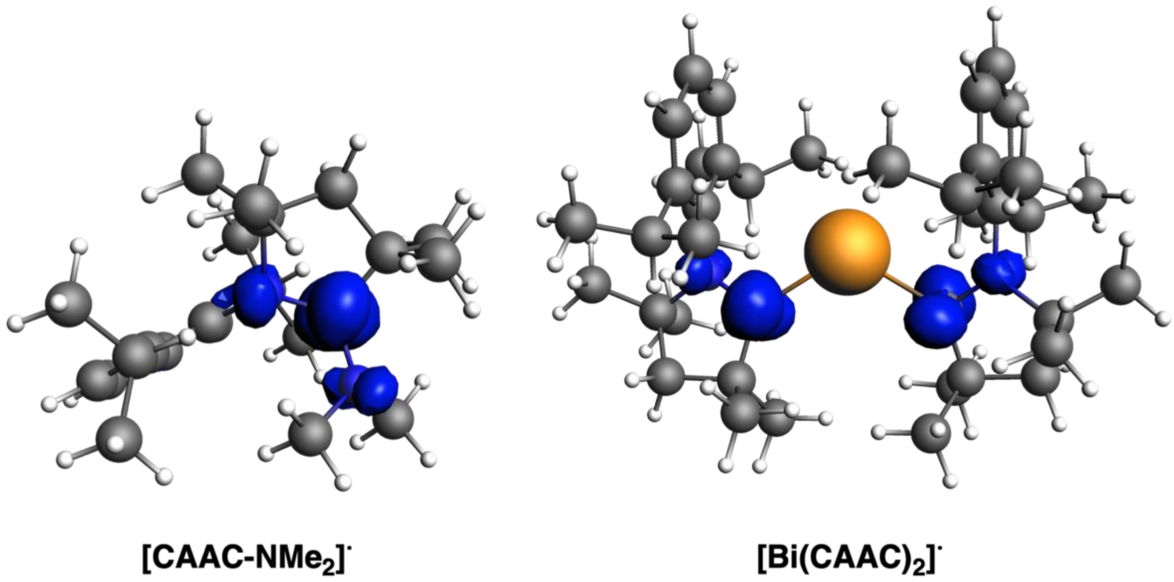

Figure S31. Spin densitiy plot of compounds **7** and **8** at their doublet state (isovalue = 0.005).

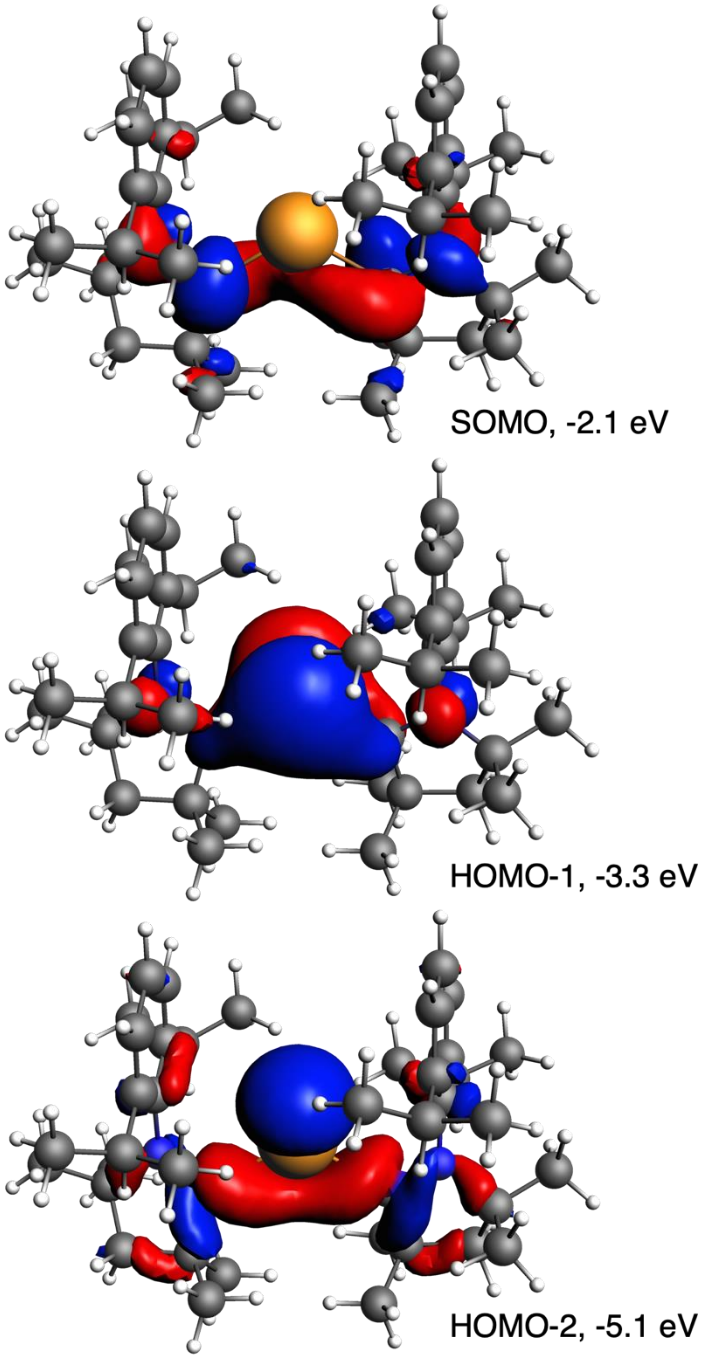

Figure S32. Selected molecular orbitals of compound **8**. SOMO is singly-occupied, whereas both HOMO-1 and HOMO-2 are doubly occupied.

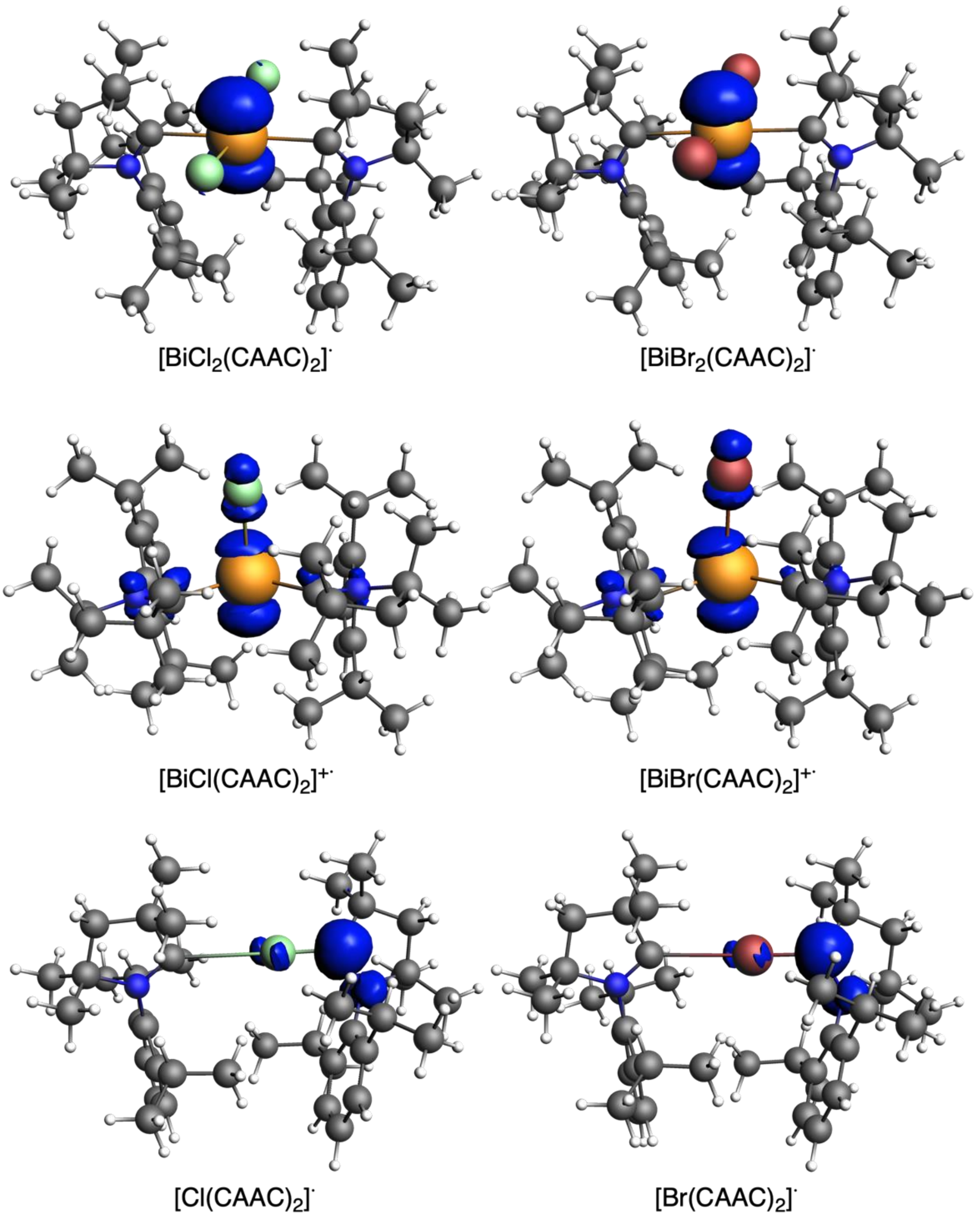

Figure S33. Spin density plot of a series of compounds for comparison to above compounds **7** and **8**. Computed at their doublet state (isovalue = 0.005).

Table S16. Cartesian coordinates (in Å) and ADF total bonding energies (in kcal mol^{-1}) of all systems under analysis. Computed at ZORA-BLYP-D3(BJ)/TZ2P in THF.

Compound 1 (-14043.1)

1	Bi	0.37395405	0.90101279	2.05070947
2	Cl	-0.05627451	3.39738934	1.73744010
3	Cl	2.66195982	1.12716098	3.33603086
4	Cl	-1.74599457	-0.44806720	0.67874453
5	N	-1.16623873	-0.35159409	4.68483615
6	C	-1.24656923	0.69721891	3.91897499
7	C	0.10421810	-1.00053463	5.01857036
8	C	0.58508455	-2.11056565	4.29550560
9	C	1.82586373	-2.64446313	4.68186620
10	H	2.22473830	-3.49692734	4.14024060
11	C	2.55611797	-2.09795070	5.73165257
12	H	3.51743092	-2.52542205	6.00632039
13	C	2.05982744	-0.99474916	6.42445307
14	H	2.64470402	-0.56382616	7.23146584
15	C	0.82748392	-0.42192431	6.09120336
16	C	0.34726421	0.80591303	6.86694277
17	C	1.11063719	2.07790625	6.43524484
18	H	0.74153798	2.94367838	6.99794499
19	H	0.98917396	2.27549681	5.36734629
20	H	2.18349820	1.97053794	6.62872320
21	C	0.44337628	0.60623104	8.39613066
22	H	1.48553945	0.55687157	8.72964446
23	H	-0.05637265	-0.31513296	8.71408078
24	H	-0.02967873	1.45125016	8.90929087
25	C	-0.14435292	-2.74647853	3.11768351
26	H	-1.02097419	-2.14050143	2.87622409
27	C	0.74280728	-2.79362955	1.85520590
28	H	1.15662461	-1.81256385	1.59319333
29	H	0.16046888	-3.13836926	0.99817644
30	H	1.59225479	-3.47185991	1.98922181
31	C	-0.62460671	-4.17567320	3.46485994
32	H	-1.21254872	-4.19575592	4.38811120
33	H	0.23126958	-4.84644966	3.60302727
34	H	-1.24219473	-4.57459702	2.65204341
35	C	-2.53212161	-0.74789967	5.33694302
36	C	-2.36063747	-1.30716628	6.74732369
37	H	-1.95908969	-0.56678316	7.44053761
38	H	-1.70963501	-2.18696246	6.74889059
39	H	-3.34425207	-1.61571860	7.11649397
40	C	-3.19544926	-1.79274397	4.42271641
41	H	-4.21607355	-1.96105892	4.78168004
42	H	-2.66760356	-2.74665215	4.45336319
43	H	-3.24507688	-1.44941124	3.38594174
44	C	-3.25720061	0.61023245	5.26395547
45	C	-2.55483070	1.43882934	4.13478691
46	C	-3.41566126	1.57733851	2.86073758
47	H	-4.31124848	2.15807129	3.10781754
48	H	-3.72202143	0.60600550	2.46843814
49	H	-2.87066394	2.10489798	2.07324886
50	C	-2.19403553	2.86017136	4.65278991
51	H	-3.12537225	3.36810951	4.92839788
52	H	-1.68615075	3.44379191	3.88253964
53	H	-1.55418252	2.81528068	5.53842135
54	Bi	-0.37395405	-0.90101279	-2.05070947
55	Cl	0.05627451	-3.39738934	-1.73744010

56	Cl	-2.66195982	-1.12716098	-3.33603086
57	Cl	1.74599457	0.44806720	-0.67874453
58	N	1.16623873	0.35159409	-4.68483615
59	C	1.24656923	-0.69721891	-3.91897499
60	C	-0.10421810	1.00053463	-5.01857036
61	C	-0.58508455	2.11056565	-4.29550560
62	C	-1.82586373	2.64446313	-4.68186620
63	H	-2.22473830	3.49692734	-4.14024060
64	C	-2.55611797	2.09795070	-5.73165257
65	H	-3.51743092	2.52542205	-6.00632039
66	C	-2.05982744	0.99474916	-6.42445307
67	H	-2.64470402	0.56382616	-7.23146584
68	C	-0.82748392	0.42192431	-6.09120336
69	C	-0.34726421	-0.80591303	-6.86694277
70	C	-1.11063719	-2.07790625	-6.43524484
71	H	-0.74153798	-2.94367838	-6.99794499
72	H	-0.98917396	-2.27549681	-5.36734629
73	H	-2.18349820	-1.97053794	-6.62872320
74	C	-0.44337628	-0.60623104	-8.39613066
75	H	-1.48553945	-0.55687157	-8.72964446
76	H	0.05637265	0.31513296	-8.71408078
77	H	0.02967873	-1.45125016	-8.90929087
78	C	0.14435292	2.74647853	-3.11768351
79	H	1.02097419	2.14050143	-2.87622409
80	C	-0.74280728	2.79362955	-1.85520590
81	H	-1.15662461	1.81256385	-1.59319333
82	H	-0.16046888	3.13836926	-0.99817644
83	H	-1.59225479	3.47185991	-1.98922181
84	C	0.62460671	4.17567320	-3.46485994
85	H	1.21254872	4.19575592	-4.38811120
86	H	-0.23126958	4.84644966	-3.60302727
87	H	1.24219473	4.57459702	-2.65204341
88	C	2.53212161	0.74789967	-5.33694302
89	C	2.36063747	1.30716628	-6.74732369
90	H	1.95908969	0.56678316	-7.44053761
91	H	1.70963501	2.18696246	-6.74889059
92	H	3.34425207	1.61571860	-7.11649397
93	C	3.19544926	1.79274397	-4.42271641
94	H	4.21607355	1.96105892	-4.78168004
95	H	2.66760356	2.74665215	-4.45336319
96	H	3.24507688	1.44941124	-3.38594174
97	C	3.25720061	-0.61023245	-5.26395547
98	C	2.55483070	-1.43882934	-4.13478691
99	C	3.41566126	-1.57733851	-2.86073758
100	H	4.31124848	-2.15807129	-3.10781754
101	H	3.72202143	-0.60600550	-2.46843814
102	H	2.87066394	-2.10489798	-2.07324886
103	C	2.19403553	-2.86017136	-4.65278991
104	H	3.12537225	-3.36810951	-4.92839788
105	H	1.68615075	-3.44379191	-3.88253964
106	H	1.55418252	-2.81528068	-5.53842135
107	H	3.16378958	-1.13329730	-6.22070666
108	H	4.32142510	-0.47468762	-5.05872344
109	H	-0.70603173	0.97261670	6.63439765
110	H	-3.16378958	1.13329730	6.22070666
111	H	-4.32142510	0.47468762	5.05872344
112	H	0.70603173	-0.97261670	-6.63439765

Compound 3’ (-13985.1)

1	Bi	0.31056977	0.96869226	2.11542142

2	Br	-0.12724452	3.61460881	1.76848847
3	Br	2.76560023	1.13777588	3.40473856
4	Br	-1.90113890	-0.44877134	0.62873824
5	N	-1.18439462	-0.33285283	4.77183905
6	C	-1.27729660	0.72731879	4.02165921
7	C	0.08826108	-0.98090255	5.09598737
8	C	0.57121587	-2.08277261	4.36248048
9	C	1.81085038	-2.62129533	4.74586403
10	H	2.21145364	-3.46711675	4.19456053
11	C	2.53825329	-2.08725207	5.80360914
12	H	3.49914264	-2.51783959	6.07614209
13	C	2.03925225	-0.99292047	6.50862454
14	H	2.62233929	-0.57182244	7.32246243
15	C	0.80825201	-0.41585519	6.17852387
16	C	0.32444284	0.80042844	6.96869537
17	C	1.08186377	2.08178719	6.55205983
18	H	0.70793726	2.93901158	7.12436103
19	H	0.96117163	2.29089685	5.48592136
20	H	2.15512589	1.97688929	6.74491722
21	C	0.42272391	0.58470616	8.49555564
22	H	1.46525017	0.53708656	8.82833495
23	H	-0.07225972	-0.34254769	8.80406322
24	H	-0.05438866	1.42174369	9.01829153
25	C	-0.15423285	-2.70327144	3.17603539
26	H	-1.03262580	-2.09666712	2.94293301
27	C	0.73352527	-2.72892834	1.91368917
28	H	1.14374992	-1.74295160	1.66724868
29	H	0.15430126	-3.06745575	1.05186497
30	H	1.58623659	-3.40524115	2.03858360
31	C	-0.63340482	-4.13908440	3.49986945
32	H	-1.21705947	-4.17582838	4.42531126
33	H	0.22333780	-4.81195166	3.62134155
34	H	-1.25461158	-4.52199850	2.68213170
35	C	-2.54444204	-0.74710102	5.42693284
36	C	-2.36112456	-1.33555806	6.82397838
37	H	-1.96147370	-0.60722174	7.53092975
38	H	-1.70333588	-2.21001130	6.80301471
39	H	-3.34037788	-1.65964224	7.19147740
40	C	-3.21153651	-1.77572206	4.49613550
41	H	-4.22941846	-1.95280034	4.85855747
42	H	-2.68171408	-2.72886099	4.50420308
43	H	-3.26844457	-1.41247933	3.46640968
44	C	-3.27508478	0.60743192	5.38718844
45	C	-2.58594543	1.46012348	4.26733480
46	C	-3.46445267	1.62756339	3.00922048
47	H	-4.35541794	2.20370169	3.28213130
48	H	-3.77924315	0.66530453	2.60145386
49	H	-2.93016073	2.17153939	2.22548836
50	C	-2.22122288	2.87064401	4.81340596
51	H	-3.15058437	3.36934576	5.11201593
52	H	-1.72503633	3.47375557	4.05050756
53	H	-1.57026956	2.80775685	5.68952463
54	Bi	-0.31056977	-0.96869226	-2.11542142
55	Br	0.12724452	-3.61460881	-1.76848847
56	Br	-2.76560023	-1.13777588	-3.40473856
57	Br	1.90113890	0.44877134	-0.62873824
58	N	1.18439462	0.33285283	-4.77183905
59	C	1.27729660	-0.72731879	-4.02165921
60	C	-0.08826108	0.98090255	-5.09598737
61	C	-0.57121587	2.08277261	-4.36248048
62	C	-1.81085038	2.62129533	-4.74586403

63	H	-2.21145364	3.46711675	-4.19456053
64	C	-2.53825329	2.08725207	-5.80360914
65	H	-3.49914264	2.51783959	-6.07614209
66	C	-2.03925225	0.99292047	-6.50862454
67	H	-2.62233929	0.57182244	-7.32246243
68	C	-0.80825201	0.41585519	-6.17852387
69	C	-0.32444284	-0.80042844	-6.96869537
70	C	-1.08186377	-2.08178719	-6.55205983
71	H	-0.70793726	-2.93901158	-7.12436103
72	H	-0.96117163	-2.29089685	-5.48592136
73	H	-2.15512589	-1.97688929	-6.74491722
74	C	-0.42272391	-0.58470616	-8.49555564
75	H	-1.46525017	-0.53708656	-8.82833495
76	H	0.07225972	0.34254769	-8.80406322
77	H	0.05438866	-1.42174369	-9.01829153
78	C	0.15423285	2.70327144	-3.17603539
79	H	1.03262580	2.09666712	-2.94293301
80	C	-0.73352527	2.72892834	-1.91368917
81	H	-1.14374992	1.74295160	-1.66724868
82	H	-0.15430126	3.06745575	-1.05186497
83	H	-1.58623659	3.40524115	-2.03858360
84	C	0.63340482	4.13908440	-3.49986945
85	H	1.21705947	4.17582838	-4.42531126
86	H	-0.22333780	4.81195166	-3.62134155
87	H	1.25461158	4.52199850	-2.68213170
88	C	2.54444204	0.74710102	-5.42693284
89	C	2.36112456	1.33555806	-6.82397838
90	H	1.96147370	0.60722174	-7.53092975
91	H	1.70333588	2.21001130	-6.80301471
92	H	3.34037788	1.65964224	-7.19147740
93	C	3.21153651	1.77572206	-4.49613550
94	H	4.22941846	1.95280034	-4.85855747
95	H	2.68171408	2.72886099	-4.50420308
96	H	3.26844457	1.41247933	-3.46640968
97	C	3.27508478	-0.60743192	-5.38718844
98	C	2.58594543	-1.46012348	-4.26733480
99	C	3.46445267	-1.62756339	-3.00922048
100	H	4.35541794	-2.20370169	-3.28213130
101	H	3.77924315	-0.66530453	-2.60145386
102	H	2.93016073	-2.17153939	-2.22548836
103	C	2.22122288	-2.87064401	-4.81340596
104	H	3.15058437	-3.36934576	-5.11201593
105	H	1.72503633	-3.47375557	-4.05050756
106	H	1.57026956	-2.80775685	-5.68952463
107	H	3.17582010	-1.11328398	-6.35259326
108	H	4.34058338	-0.47219862	-5.18864705
109	H	-0.72966168	0.96571284	6.73947201
110	H	-3.17582010	1.11328398	6.35259326
111	H	-4.34058338	0.47219862	5.18864705
112	H	0.72966168	-0.96571284	-6.73947201

[$BiCl_3$(CAAC)(thf)] (-8618.3)

1	Bi	9.21826181	10.29139899	13.44203084
2	Cl	7.61790982	9.74475226	15.34102110
3	Cl	11.37579538	9.20968760	14.64432271
4	Cl	7.25019147	11.25692891	11.79416023
5	N	10.30723079	13.34999163	13.34018713
6	C	9.42164964	12.67901914	14.01976076
7	C	11.54144340	12.72081332	12.85688730
8	C	11.63609597	12.16622698	11.56057783
9	C	12.82517009	11.49281703	11.23257287

10	H	12.92647011	11.05073557	10.24613392
11	C	13.87193069	11.38057529	12.14226421
12	H	14.77754258	10.84615640	11.86578499
13	C	13.75921889	11.95463089	13.40673894
14	H	14.57983851	11.86006562	14.11169845
15	C	12.60091117	12.63802004	13.79491027
16	C	12.54015274	13.24402037	15.19786501
17	C	12.43757136	12.16002388	16.29276688
18	H	12.37648347	12.63518715	17.27905578
19	H	11.56035426	11.52545739	16.14798155
20	H	13.31710278	11.50715673	16.27808148
21	C	13.74729454	14.17255795	15.46752345
22	H	14.68060930	13.60236887	15.52828991
23	H	13.86330986	14.92476036	14.68022083
24	H	13.60991136	14.69024943	16.42383876
25	C	10.55080930	12.28991265	10.49547163
26	H	9.63639355	12.65839248	10.96609016
27	C	10.21015917	10.93402535	9.83934499
28	H	9.96085308	10.17206907	10.58433582
29	H	9.34028614	11.04450121	9.18505416
30	H	11.04512863	10.55419402	9.24166292
31	C	10.98505310	13.30038852	9.40534171
32	H	11.28700766	14.25981495	9.83711508
33	H	11.83895745	12.90875307	8.84043989
34	H	10.16212591	13.47911607	8.70378563
35	C	9.98321585	14.87653120	13.24693520
36	C	11.24296940	15.73866494	13.24151345
37	H	11.78832542	15.68265482	14.18404483
38	H	11.91364229	15.45455055	12.42415495
39	H	10.94704773	16.78116277	13.08516990
40	C	9.17356280	15.10690071	11.95761473
41	H	8.78505920	16.13024894	11.97736947
42	H	9.79580663	15.00203858	11.06814585
43	H	8.32928845	14.41708111	11.88081351
44	C	9.10143495	15.02177051	14.50405854
45	C	8.50911450	13.60146081	14.80218441
46	C	7.01568380	13.47756915	14.42555925
47	H	6.44078079	14.15663582	15.06499324
48	H	6.83655913	13.73855469	13.38104643
49	H	6.65677348	12.45847052	14.58534791
50	C	8.68053389	13.23297810	16.30324865
51	H	8.12101271	13.95987504	16.90308006
52	H	8.29120732	12.23223917	16.50617852
53	H	9.72990381	13.27089881	16.60949396
54	O	8.73999433	7.77276987	12.47282999
55	C	9.82234276	7.25143013	11.62541133
56	C	7.48332899	7.53138611	11.76197306
57	C	9.27599091	7.22059881	10.17475902
58	H	10.68257017	7.91319414	11.76352969
59	H	10.09012569	6.25211607	11.98690907
60	C	7.82906990	7.77248644	10.29223481
61	H	7.16568116	6.49568679	11.94819279
62	H	6.74569544	8.22493199	12.17076401
63	H	9.27307343	6.19417210	9.79577249
64	H	9.88614583	7.82695437	9.49996772
65	H	7.13296831	7.27023483	9.61376598
66	H	7.80166927	8.84699035	10.08752705
67	H	11.64002190	13.85643508	15.27237992
68	H	8.31383121	15.76231806	14.34854015
69	H	9.71311776	15.35340406	15.34913816

[$BiBr_3$(CAAC)(thf)] (-8588.4)

1	Bi	9.21658114	10.27365645	13.44990533
2	Br	7.61613006	9.61602299	15.51319128
3	Br	11.61728171	9.11216167	14.45250076
4	Br	7.12991371	11.27471210	11.69790699
5	N	10.30174092	13.34896903	13.34589616
6	C	9.42563733	12.66841639	14.02970005
7	C	11.55178292	12.75367508	12.86289425
8	C	11.66904048	12.21352839	11.56368526
9	C	12.88816040	11.60168021	11.22650527
10	H	13.00668206	11.17195655	10.23648081
11	C	13.94162206	11.53230581	12.13200881
12	H	14.87148568	11.04531200	11.84865802
13	C	13.80322409	12.08544265	13.40328786
14	H	14.62785301	12.02047680	14.10675197
15	C	12.61498147	12.70984365	13.79944477
16	C	12.52369619	13.29097707	15.21121933
17	C	12.41558103	12.18448296	16.28292899
18	H	12.33836042	12.63783102	17.27834044
19	H	11.54347188	11.54776852	16.11598329
20	H	13.29910649	11.53735667	16.26499969
21	C	13.71064624	14.23132228	15.52294578
22	H	14.65169475	13.67528407	15.59480345
23	H	13.83176902	14.99891788	14.75128160
24	H	13.54473671	14.73029521	16.48462581
25	C	10.57007004	12.27574906	10.50813875
26	H	9.64675969	12.61923750	10.98105406
27	C	10.27677465	10.89135940	9.88917530
28	H	10.06675420	10.13777798	10.65550199
29	H	9.39694228	10.94991838	9.24156218
30	H	11.12178089	10.52971191	9.29415762
31	C	10.94771641	13.27604584	9.38913481
32	H	11.21493401	14.25739555	9.79384226
33	H	11.80931560	12.90755500	8.82026002
34	H	10.10863147	13.40398105	8.69559049
35	C	9.95529271	14.87087114	13.24672612
36	C	11.20192291	15.75139844	13.22416398
37	H	11.76042507	15.70475931	14.15967067
38	H	11.86639405	15.47663578	12.39871800
39	H	10.88822856	16.78915596	13.07064099
40	C	9.13172700	15.07755467	11.96252590
41	H	8.72967971	16.09583682	11.97461993
42	H	9.74807962	14.97140733	11.06894450
43	H	8.29664488	14.37497733	11.89927217
44	C	9.08243054	15.01285901	14.50921341
45	C	8.51461935	13.58638967	14.82451122
46	C	7.01423607	13.44825405	14.48703355
47	H	6.44896997	14.11685057	15.14587820
48	H	6.80473681	13.71398418	13.44961328
49	H	6.66885565	12.42475030	14.65066897
50	C	8.72247190	13.23033943	16.32451244
51	H	8.16528729	13.95472311	16.92964294
52	H	8.35030569	12.22660600	16.54380553
53	H	9.77655595	13.28466991	16.60993039
54	O	8.75432303	7.76734762	12.41236070
55	C	9.80872067	7.19094825	11.56956606
56	C	7.48830481	7.49397562	11.73457676
57	C	9.27004074	7.20989059	10.11664147
58	H	10.70668669	7.79313363	11.72754349
59	H	10.00503905	6.16962983	11.91787989
60	C	7.80024234	7.70280146	10.25096570

61	H	7.18878702	6.45883587	11.95287479
62	H	6.74879369	8.18743183	12.14034689
63	H	9.31352547	6.20576248	9.68444334
64	H	9.85647074	7.87558010	9.47780191
65	H	7.11233677	7.15175551	9.60276122
66	H	7.72537032	8.76829367	10.01458135
67	H	11.61440055	13.89014721	15.28100568
68	H	8.28130366	15.73881576	14.35364907
69	H	9.69470498	15.36266504	15.34651136

thf (-1594.4)

1	H	7.81223791	8.85238204	10.08153424
2	O	8.74104951	7.78827527	12.47024596
3	C	9.81467001	7.25630352	11.63314486
4	C	7.49549204	7.53069938	11.76743896
5	C	9.27449857	7.21774933	10.17654938
6	H	10.67516788	7.92036258	11.76054980
7	H	10.08474962	6.25003972	11.98470645
8	C	7.83029612	7.77706545	10.29274721
9	H	7.17624060	6.48812671	11.93347367
10	H	6.74194312	8.20737925	12.18095637
11	H	9.26238381	6.18918154	9.80118249
12	H	9.88932609	7.81597673	9.49778118
13	H	7.13060091	7.27941049	9.61332644

Compound 7 (-7743.6)

1	C	-0.89579381	-0.81582572	0.66944178
2	N	-2.30840123	-0.71294660	0.59429434
3	N	-0.12331784	-0.82789162	-0.51999782
4	C	-0.46695963	-1.66163559	1.87582799
5	C	-2.92829046	0.48864647	0.08613137
6	C	-2.96251708	-1.56399488	1.66856051
7	C	0.06029238	-3.05666823	1.44003346
8	C	0.63283230	-1.00446641	2.75473890
9	C	-1.79445949	-1.75370612	2.67684972
10	C	-3.89431427	0.39711590	-0.96114441
11	C	-2.58534818	1.77236822	0.61153827
12	C	-4.15242653	-0.85780121	2.33163588
13	C	-3.42719146	-2.92703158	1.09761590
14	H	-0.68037739	-3.61037679	0.85525162
15	H	0.95430619	-2.93853763	0.81717313
16	H	0.32363111	-3.65734987	2.32128894
17	H	1.59494220	-0.97097605	2.23018820
18	H	0.36002378	0.01806790	3.03832466
19	H	0.77867653	-1.58919625	3.67437194
20	H	-1.87955473	-2.69906872	3.22439783
21	H	-1.83124237	-0.94054860	3.41272942
22	C	-4.47847726	1.57290693	-1.45863669
23	C	-4.31557390	-0.93129045	-1.58672718
24	C	-3.17811779	2.91640887	0.05865920
25	C	-1.65099772	1.96618605	1.80526966
26	H	-3.84819539	0.08538033	2.79263288
27	H	-4.95058103	-0.64570346	1.61356303
28	H	-4.56309546	-1.50668505	3.11492618
29	H	-4.34158382	-2.82154950	0.50831922
30	H	-2.65836262	-3.37289229	0.46035392
31	H	-3.63693053	-3.62244898	1.92055843
32	C	-4.12245686	2.83021310	-0.96925181
33	H	-5.21731669	1.50250522	-2.25549671
34	C	-4.13615250	-0.94313699	-3.12425571
35	C	-5.77804739	-1.29223753	-1.23339686

36	H	-3.66331912	-1.70279969	-1.17201297
37	H	-2.91974838	3.89457723	0.46226818
38	C	-0.41801159	2.82411870	1.44380653
39	C	-2.38980602	2.58162630	3.01746943
40	H	-1.28338193	0.98233655	2.09661116
41	H	-4.58136194	3.73123408	-1.37482466
42	H	-4.34100115	-1.94754056	-3.51807121
43	H	-4.82851374	-0.24660247	-3.61382958
44	H	-3.11743763	-0.66281992	-3.41183678
45	H	-5.94304171	-1.31296730	-0.15083239
46	H	-6.03904637	-2.27954263	-1.63776582
47	H	-6.47226582	-0.55735930	-1.66164067
48	H	0.11674242	2.40096613	0.58837647
49	H	-0.70994825	3.85176825	1.19130809
50	H	0.27588828	2.87157033	2.29372111
51	H	-3.27316851	1.99461424	3.29371520
52	H	-2.72292069	3.60554717	2.80452680
53	H	-1.72009789	2.61984625	3.88722634
54	H	1.50877814	0.14243400	0.40253920
55	H	-0.05119329	-1.26962156	-2.56379702
56	C	-0.79129244	-0.95734736	-1.81529932
57	H	-1.57255881	-1.71632438	-1.75333212
58	H	-1.24592196	-0.01177673	-2.15946094
59	C	1.05918634	0.04291067	-0.58612262
60	H	1.79991231	-0.39189251	-1.27074002
61	H	0.80531096	1.05585411	-0.95361227

Compound 8 (-13430.1)

1	C	-0.93892786	-1.35033568	0.57673278
2	N	-2.22553039	-0.86911690	0.72614338
3	H	4.55251816	-4.33996723	-1.93132327
4	C	-0.43462010	-1.84274802	1.93539727
5	C	-2.77073202	0.08482503	-0.21108285
6	C	-2.85360148	-1.22896478	2.05973361
7	C	-0.20268811	-3.37140055	1.98020290
8	C	0.88181740	-1.11123693	2.30215609
9	C	-1.57806779	-1.41871543	2.91661120
10	C	-3.67480502	-0.33069633	-1.22157566
11	C	-2.32398448	1.43610014	-0.14691786
12	C	-3.76508743	-0.11238135	2.58349412
13	C	-3.66180270	-2.54455526	1.96333450
14	H	-1.11431984	-3.92426417	1.73332237
15	H	0.56675663	-3.66572703	1.26441438
16	H	0.12766340	-3.67656508	2.98313598
17	H	1.65586356	-1.29941271	1.55201220
18	H	0.72019708	-0.02862110	2.34639673
19	H	1.25235654	-1.44914043	3.28071649
20	H	-1.72918005	-2.15308363	3.71521296
21	H	-1.31949066	-0.46280864	3.38831189
22	C	-4.13928062	0.62532533	-2.14278617
23	C	-4.15204399	-1.77328665	-1.35285105
24	C	-2.80881399	2.34730144	-1.09586704
25	C	-1.34932921	1.93652366	0.91894530
26	H	-3.21377376	0.81786462	2.74000857
27	H	-4.58905419	0.08354285	1.88721264
28	H	-4.19814571	-0.41959320	3.54310086
29	H	-4.60093373	-2.39606956	1.42520676
30	H	-3.09083456	-3.32561794	1.45313917
31	H	-3.90410356	-2.89886676	2.97334563
32	C	-3.71043484	1.95068616	-2.08821106
33	H	-4.83524919	0.31998285	-2.92199037

34	C	-3.89711050	-2.34711807	-2.76585201
35	C	-5.65142146	-1.89435457	-0.98934585
36	H	-3.56831741	-2.37190217	-0.64830202
37	H	-2.47897782	3.38409030	-1.05775528
38	C	0.01697790	2.32360602	0.30921006
39	C	-1.93519102	3.12251964	1.72168054
40	H	-1.17108780	1.11481696	1.61571449
41	H	-4.07585189	2.67488283	-2.81566848
42	H	-4.15537134	-3.41403925	-2.78938316
43	H	-4.50633976	-1.83811446	-3.52390123
44	H	-2.84227934	-2.24359868	-3.03917926
45	H	-5.86615425	-1.46320556	-0.00459022
46	H	-5.96313828	-2.94723378	-0.98069914
47	H	-6.27030340	-1.36376195	-1.72495493
48	H	0.46440508	1.46827420	-0.20682113
49	H	-0.09196104	3.14487195	-0.41113559
50	H	0.70423231	2.65363950	1.10003430
51	H	-2.92295520	2.88425368	2.13350209
52	H	-2.04198328	4.01522950	1.09258281
53	H	-1.26731463	3.37905462	2.55448428
54	Bi	-0.14090966	-1.31308043	-1.58326684
55	H	3.27652958	-4.93783211	-3.00184192
56	H	1.30372137	-4.23484340	-4.13251239
57	H	1.16496497	-5.29448477	-2.07394239
58	N	2.36066329	-2.25763698	-3.23084598
59	C	1.71619436	-2.59120223	-2.05455657
60	C	1.64372871	-1.62579049	-4.31333328
61	C	1.76787419	-0.23214499	-4.54610747
62	C	1.01363321	0.34638012	-5.58267148
63	H	1.10141585	1.41439645	-5.77336790
64	C	0.14218442	-0.41806190	-6.35705337
65	H	-0.43905412	0.04985837	-7.15104757
66	C	0.01644369	-1.78857466	-6.11047389
67	H	-0.66490791	-2.38014181	-6.71955657
68	C	0.75403655	-2.41625145	-5.09652321
69	C	0.58015639	-3.92049156	-4.88756272
70	C	-0.82597875	-4.26440866	-4.34553557
71	H	-0.91320777	-5.34680178	-4.17975127
72	H	-1.00917401	-3.75064267	-3.39648183
73	H	-1.60614999	-3.96549560	-5.05774244
74	C	0.86660069	-4.71760687	-6.18235863
75	H	0.10779034	-4.51760835	-6.94937261
76	H	1.84502915	-4.46322317	-6.60660678
77	H	0.85205748	-5.79498474	-5.97152434
78	C	2.67852068	0.66160345	-3.71055068
79	H	3.04928473	0.05697266	-2.87843179
80	C	1.92131081	1.86565036	-3.10481209
81	H	1.04963746	1.52894551	-2.53534878
82	H	2.58195613	2.42104937	-2.42602705
83	H	1.58148925	2.56073678	-3.88358193
84	C	3.88420035	1.15616385	-4.54582886
85	H	4.41667174	0.32468408	-5.02203250
86	H	3.54870045	1.83349828	-5.34227406
87	H	4.59457255	1.70366942	-3.91225797
88	C	3.75334708	-2.84973979	-3.34481338
89	C	4.09364952	-3.23822554	-4.78873990
90	H	3.40363844	-3.99154149	-5.17644680
91	H	4.06333276	-2.36441412	-5.45024946
92	H	5.10821754	-3.65339815	-4.82207578
93	C	4.82564357	-1.86993222	-2.81295664
94	H	5.77792197	-2.40190759	-2.69212191

95	H	4.98902062	-1.04279699	-3.50758729
96	H	4.53866497	-1.45472029	-1.84257181
97	C	3.60367807	-4.07432895	-2.40991460
98	C	2.49845877	-3.71052182	-1.36254938
99	C	3.12945273	-3.27469706	-0.01931694
100	H	3.70082334	-4.10516817	0.41889356
101	H	3.80372981	-2.42255787	-0.14927894
102	H	2.35772667	-2.97993147	0.69339365
103	C	1.57190067	-4.93030140	-1.12423627
104	H	2.12802040	-5.74948383	-0.64596406
105	H	0.72634454	-4.66298028	-0.48285664

$[BiCl_2(CAAC)_2]^{\bullet}$ (-13644.6)

1	C	-0.95358401	-0.99752755	0.66729091
2	N	-2.19252052	-0.64277330	0.86667371
3	H	4.40204059	-0.16208499	-5.63299341
4	C	-0.49223372	-1.91055184	1.79069157
5	C	-2.82322018	0.48825119	0.18755800
6	C	-2.92013227	-1.43556488	2.00555297
7	C	-0.10850709	-3.32085872	1.28893558
8	C	0.76109574	-1.24501038	2.41725803
9	C	-1.69165684	-1.92717902	2.79523161
10	C	-3.57000517	0.32373724	-0.99728359
11	C	-2.64159981	1.76018822	0.78702395
12	C	-3.88171086	-0.56405408	2.81374350
13	C	-3.68992338	-2.59827886	1.35524928
14	H	-0.94993357	-3.82998849	0.81384739
15	H	0.69863297	-3.26423305	0.55247626
16	H	0.23510538	-3.92114592	2.13955181
17	H	1.57698041	-1.19218345	1.68937455
18	H	0.54435293	-0.22713618	2.75736422
19	H	1.09138051	-1.83581817	3.28031497
20	H	-1.86227968	-2.92199874	3.21493584
21	H	-1.49068489	-1.24360289	3.62729770
22	C	-4.16172566	1.46803329	-1.55920425
23	C	-3.74027203	-1.00605838	-1.72204589
24	C	-3.25652333	2.86402891	0.18292128
25	C	-1.76248510	1.98592540	2.01706005
26	H	-3.36372117	0.22652675	3.35841302
27	H	-4.64386588	-0.11142900	2.17141361
28	H	-4.39303428	-1.19786265	3.54657643
29	H	-4.54324749	-2.23584505	0.77989123
30	H	-3.04957577	-3.18873973	0.69524300
31	H	-4.07082627	-3.25268548	2.14669666
32	C	-4.01898040	2.72233025	-0.97532542
33	H	-4.73156256	1.36931523	-2.47843895
34	C	-3.23113947	-0.91376503	-3.17633358
35	C	-5.21775704	-1.46577479	-1.72152100
36	H	-3.13808688	-1.76236438	-1.21337430
37	H	-3.12240620	3.85072332	0.61656677
38	C	-0.39376119	2.57194413	1.60557617
39	C	-2.44089435	2.87622447	3.08117571
40	H	-1.56485284	1.01880650	2.48287697
41	H	-4.48450713	3.59304819	-1.43168684
42	H	-3.24079198	-1.90324293	-3.64070095
43	H	-3.85483654	-0.24140505	-3.77325836
44	H	-2.20537789	-0.53510133	-3.22173588
45	H	-5.64599055	-1.47781913	-0.71415028
46	H	-5.29965877	-2.47479611	-2.14270868
47	H	-5.83144216	-0.79317434	-2.33271041

48	H	0.13391978	1.91776232	0.90650286
49	H	-0.52268076	3.54829152	1.12360471
50	H	0.23741515	2.70915852	2.49257244
51	H	-3.43619077	2.50355232	3.34678998
52	H	-2.55052604	3.90872561	2.73104979
53	H	-1.82755547	2.89947860	3.98958107
54	Bi	0.31872153	-0.77930077	-1.64554112
55	Cl	-0.78468172	-3.24327941	-2.19864891
56	H	0.63927951	-1.27210726	-6.48118918
57	Cl	2.11313928	1.11805000	-0.76770183
58	N	1.25203569	0.39987866	-4.87115858
59	C	1.54704686	-0.49761701	-3.97240212
60	C	-0.10667340	0.88965086	-5.10047164
61	C	-0.59330381	2.05850605	-4.47906360
62	C	-1.90380907	2.46253772	-4.78650195
63	H	-2.30523631	3.35333090	-4.31232312
64	C	-2.69973009	1.74095416	-5.66997269
65	H	-3.71201023	2.07333114	-5.88899800
66	C	-2.20545066	0.57540310	-6.25334386
67	H	-2.84280348	-0.00100230	-6.91751087
68	C	-0.90822598	0.12209719	-5.98279741
69	C	-0.44342423	-1.19812292	-6.59800609
70	C	-1.05799098	-2.39600317	-5.84029545
71	H	-0.68562116	-3.33779118	-6.26227407
72	H	-0.80848854	-2.37493297	-4.77631734
73	H	-2.15067570	-2.38885167	-5.93208676
74	C	-0.74761985	-1.29323427	-8.10896738
75	H	-1.82474082	-1.35915263	-8.29896641
76	H	-0.36190552	-0.42574981	-8.65559915
77	H	-0.28393480	-2.19604910	-8.52355093
78	C	0.19933941	2.88485538	-3.47296047
79	H	1.14468618	2.37623064	-3.27008017
80	C	-0.55657815	3.00215841	-2.13178644
81	H	-0.84823604	2.02101176	-1.74636331
82	H	0.08249858	3.47662169	-1.38223636
83	H	-1.46860284	3.59710118	-2.23973010
84	C	0.50295705	4.30073767	-4.01873544
85	H	0.97821975	4.27108729	-5.00428954
86	H	-0.42156510	4.88240568	-4.11601352
87	H	1.16874884	4.83582118	-3.33124685
88	C	2.44801970	0.81017891	-5.79572386
89	C	2.01748824	1.07572374	-7.23840315
90	H	1.62342871	0.18121292	-7.72284094
91	H	1.26368293	1.86786356	-7.28921450
92	H	2.89302416	1.40782834	-7.80704407
93	C	3.09462556	2.07385589	-5.20230630
94	H	4.02379397	2.27918510	-5.74468228
95	H	2.44297804	2.94169338	-5.31266520
96	H	3.33195519	1.94774565	-4.14303394
97	C	3.34279096	-0.43160012	-5.61773967
98	C	2.92010755	-1.08214921	-4.25908752
99	C	3.93035644	-0.81134529	-3.12162372
100	H	4.88980910	-1.27545659	-3.37857629
101	H	4.08677596	0.25721230	-2.95823866
102	H	3.57650463	-1.23875212	-2.17876889
103	C	2.72796403	-2.61472742	-4.40219127
104	H	3.68456961	-3.07543919	-4.67721832
105	H	2.38075274	-3.05211915	-3.46061990
106	H	1.99310937	-2.85410877	-5.17770926
107	H	3.16507476	-1.13478506	-6.43875690

[$BiBr_2(CAAC)_2]^•$ (-13626.0)

1	C	-0.93861988	-0.97418836	0.66867640
2	N	-2.18432535	-0.63643038	0.86109717
3	H	4.38104176	-0.22336761	-5.65639513
4	C	-0.46069089	-1.85090391	1.81577163
5	C	-2.82804656	0.48370505	0.17453717
6	C	-2.90086183	-1.43448153	2.00121246
7	C	-0.02287395	-3.26008348	1.36132261
8	C	0.76440148	-1.12740612	2.43655433
9	C	-1.66832786	-1.88094040	2.81073354
10	C	-3.56618220	0.30753979	-1.01413820
11	C	-2.66606585	1.75977921	0.77105027
12	C	-3.89869629	-0.58331431	2.78610498
13	C	-3.62474845	-2.62947436	1.35501256
14	H	-0.84393144	-3.81769688	0.90574954
15	H	0.78158283	-3.19808569	0.62229277
16	H	0.34400978	-3.81715174	2.23146332
17	H	1.58608404	-1.06734579	1.71602829
18	H	0.51272303	-0.10816416	2.74637332
19	H	1.10221696	-1.68454059	3.31886781
20	H	-1.81837078	-2.87283618	3.24488990
21	H	-1.49254245	-1.17934982	3.63348545
22	C	-4.16185901	1.44419030	-1.58717748
23	C	-3.73115558	-1.02879165	-1.72679242
24	C	-3.28586374	2.85541061	0.15693279
25	C	-1.80593093	2.00026204	2.01161689
26	H	-3.41201173	0.22951296	3.32696328
27	H	-4.66572265	-0.16207997	2.12833675
28	H	-4.40009752	-1.22313328	3.52048657
29	H	-4.48276504	-2.30221188	0.76596598
30	H	-2.95702779	-3.20437263	0.70828781
31	H	-3.99268620	-3.28752927	2.14951328
32	C	-4.03481274	2.70249213	-1.00852848
33	H	-4.72399517	1.33522095	-2.50993366
34	C	-3.23831521	-0.94611389	-3.18700551
35	C	-5.20435392	-1.50142785	-1.70434365
36	H	-3.11732696	-1.77558211	-1.21760523
37	H	-3.16643803	3.84478844	0.58873957
38	C	-0.44170198	2.60647269	1.61704255
39	C	-2.51232980	2.88187425	3.06508508
40	H	-1.60022695	1.03699389	2.48194540
41	H	-4.50259242	3.56741695	-1.47347483
42	H	-3.23173455	-1.94211324	-3.63743551
43	H	-3.88050565	-0.29606099	-3.78906226
44	H	-2.22065637	-0.54871855	-3.24575070
45	H	-5.61978595	-1.50890856	-0.69155093
46	H	-5.28213583	-2.51457498	-2.11632129
47	H	-5.83113856	-0.83876425	-2.31305089
48	H	0.10413342	1.95680841	0.92846433
49	H	-0.57815180	3.57968506	1.13127215
50	H	0.17749997	2.75438097	2.51072892
51	H	-3.50358305	2.49206456	3.32099894
52	H	-2.63694950	3.91103274	2.71023095
53	H	-1.90949012	2.91903312	3.98002545
54	Bi	0.32173778	-0.77284224	-1.64558264
55	Br	-0.77821306	-3.41574670	-2.18688161
56	H	0.62635583	-1.24773240	-6.50800786
57	Br	2.25651448	1.17178624	-0.69756790
58	N	1.24772922	0.39041151	-4.86416341
59	C	1.53390355	-0.52228763	-3.97639214
60	C	-0.10705623	0.89440112	-5.08897594

61	C	-0.58697444	2.05884663	-4.45469274
62	C	-1.89787961	2.47035359	-4.74998804
63	H	-2.29396330	3.35637487	-4.26274469
64	C	-2.70057525	1.76188079	-5.63774491
65	H	-3.71327696	2.09916371	-5.84695133
66	C	-2.21078754	0.60451661	-6.24047734
67	H	-2.85150602	0.03975628	-6.91141137
68	C	-0.91395769	0.14361529	-5.98133112
69	C	-0.45576844	-1.16567235	-6.62394781
70	C	-1.07815161	-2.37515785	-5.89294293
71	H	-0.72427981	-3.31110523	-6.34289770
72	H	-0.81481045	-2.38418056	-4.83236616
73	H	-2.17148700	-2.35087775	-5.96981459
74	C	-0.75928844	-1.22574875	-8.13723881
75	H	-1.83658231	-1.28174245	-8.32927627
76	H	-0.36877129	-0.34823206	-8.66396273
77	H	-0.29970774	-2.12151497	-8.57106090
78	C	0.21589208	2.87747837	-3.45157723
79	H	1.15558694	2.35835022	-3.24796121
80	C	-0.53549299	3.00908132	-2.10953788
81	H	-0.84906591	2.03373354	-1.72581783
82	H	0.11642025	3.46490809	-1.35933126
83	H	-1.43368298	3.62563269	-2.21115679
84	C	0.53776123	4.28661171	-4.00413089
85	H	1.01050430	4.24511055	-4.99054235
86	H	-0.37924861	4.87984618	-4.10255005
87	H	1.21227050	4.81561032	-3.32044689
88	C	2.44902295	0.79662180	-5.78231373
89	C	2.02129410	1.11040093	-7.21576033
90	H	1.61128166	0.23704659	-7.72493332
91	H	1.28085453	1.91627534	-7.24228782
92	H	2.90126183	1.44421158	-7.77650010
93	C	3.12555523	2.02854156	-5.15529237
94	H	4.06223953	2.22328010	-5.68861365
95	H	2.49713079	2.91570674	-5.24569795
96	H	3.35413260	1.86981695	-4.09833179
97	C	3.31603938	-0.46890836	-5.63826960
98	C	2.88978556	-1.13642511	-4.28879218
99	C	3.92441728	-0.92485805	-3.16146623
100	H	4.86084677	-1.42284730	-3.43903991
101	H	4.12671494	0.13350157	-2.98381850
102	H	3.56712214	-1.35349531	-2.22035477
103	C	2.65092646	-2.65978454	-4.46290230
104	H	3.59051905	-3.13937483	-4.76307277
105	H	2.30477802	-3.10819249	-3.52640046
106	H	1.89852054	-2.86147885	-5.23196387
107	H	3.11617480	-1.15059556	-6.47217250

$[BiCl(CAAC)_2]^{+\bullet}$ (-13435.4)

1	C	-1.00918042	-1.66327057	0.37880413
2	N	-2.13064357	-1.00958490	0.60750880
3	H	5.03675921	-2.59385203	-2.45748768
4	C	-0.53712754	-2.37805342	1.64509159
5	C	-2.59549897	0.06857986	-0.26766425
6	C	-2.79847354	-1.37086156	1.96818017
7	C	-0.53616959	-3.92076934	1.53087219
8	C	0.88619718	-1.88587622	2.00788423
9	C	-1.55587929	-1.87384448	2.72429467
10	C	-3.53268657	-0.17405385	-1.29934615
11	C	-1.98651729	1.33860982	-0.07239912
12	C	-3.48077160	-0.16069494	2.60604288

13	C	-3.82719851	-2.49404249	1.73730640
14	H	-1.53616567	-4.31287953	1.33124542
15	H	0.11206867	-4.26196766	0.72309014
16	H	-0.17852750	-4.33645302	2.48055083
17	H	1.63550886	-2.27639800	1.31817275
18	H	0.94756834	-0.79248022	2.01269217
19	H	1.13304945	-2.24600054	3.01379029
20	H	-1.81153017	-2.66674013	3.43192794
21	H	-1.11244719	-1.04765723	3.29095716
22	C	-3.83944940	0.89911624	-2.15610495
23	C	-4.22071960	-1.52022853	-1.51836472
24	C	-2.33045518	2.36514244	-0.96294667
25	C	-0.98305862	1.62402113	1.04868783
26	H	-2.77027572	0.62257079	2.87582999
27	H	-4.24358140	0.26155832	1.94279978
28	H	-3.98037246	-0.49231618	3.52305289
29	H	-4.71590072	-2.12568805	1.22219648
30	H	-3.40518002	-3.32483509	1.16572622
31	H	-4.14010161	-2.87406525	2.71616683
32	C	-3.24249243	2.14893253	-1.99837303
33	H	-4.55066658	0.74499784	-2.96357853
34	C	-4.07853553	-2.03600722	-2.96777831
35	C	-5.71929224	-1.41837140	-1.13925251
36	H	-3.74549678	-2.25882745	-0.86662784
37	H	-1.87327310	3.34487687	-0.84837799
38	C	0.45636124	1.79768953	0.51517717
39	C	-1.39713338	2.85760850	1.88440307
40	H	-0.96935878	0.76534473	1.72259251
41	H	-3.48906887	2.95874345	-2.68238314
42	H	-4.55139209	-3.02107042	-3.05614313
43	H	-4.56032055	-1.36289305	-3.68622677
44	H	-3.02836656	-2.15179751	-3.25484652
45	H	-5.85898571	-1.00273790	-0.13512318
46	H	-6.18748115	-2.40952716	-1.17396354
47	H	-6.24864966	-0.76509224	-1.84398039
48	H	0.77598943	0.92860924	-0.07157598
49	H	0.53179273	2.68290857	-0.12475831
50	H	1.15341662	1.92210353	1.35270236
51	H	-2.42623514	2.76951126	2.25003325
52	H	-1.32676038	3.77925672	1.29497727
53	H	-0.72987833	2.96159014	2.74867783
54	Bi	-0.40574546	-1.45143271	-1.94914197
55	H	4.00037269	-3.90477903	-3.03869070
56	H	1.04600651	-3.74179486	-4.70628790
57	Cl	-1.01362989	-4.24140576	-1.83472478
58	N	2.16448364	-1.69035047	-3.75803039
59	C	1.79671358	-1.98360874	-2.52333556
60	C	1.29265512	-1.05863616	-4.75459051
61	C	1.22173787	0.36112160	-4.81266257
62	C	0.46014449	0.93546163	-5.84359914
63	H	0.39436178	2.01851577	-5.91105904
64	C	-0.21749704	0.15089206	-6.77445272
65	H	-0.79156902	0.62127288	-7.57077765
66	C	-0.18016538	-1.23721236	-6.66478649
67	H	-0.74419472	-1.84293092	-7.36951320
68	C	0.55872296	-1.87753430	-5.65576050
69	C	0.45152961	-3.40027599	-5.56009676
70	C	-1.01824947	-3.81290576	-5.30235560
71	H	-1.07437037	-4.88696236	-5.09221423
72	H	-1.43953404	-3.29134115	-4.44059461
73	H	-1.64106512	-3.59778508	-6.17901143

74	C	0.95582878	-4.11266437	-6.83929698
75	H	0.30121836	-3.88602965	-7.68964414
76	H	1.97224552	-3.81777056	-7.11172275
77	H	0.94023200	-5.19900792	-6.68735178
78	C	1.85812621	1.31108374	-3.79548964
79	H	2.40772878	0.71984511	-3.05720689
80	C	0.76416237	2.10271244	-3.04021169
81	H	0.05625398	1.43996813	-2.53122070
82	H	1.22460312	2.74891105	-2.28540770
83	H	0.19137130	2.73654346	-3.72663745
84	C	2.84013775	2.31214481	-4.45054293
85	H	3.60681767	1.81235986	-5.04841735
86	H	2.30399969	3.00851079	-5.10627904
87	H	3.34085028	2.90298335	-3.67377130
88	C	3.65744772	-2.02346082	-4.06465857
89	C	3.77424823	-2.85438609	-5.34303494
90	H	3.23600345	-3.80026180	-5.25659093
91	H	3.40816456	-2.30269243	-6.21454478
92	H	4.83401521	-3.08037349	-5.50741438
93	C	4.46997352	-0.73215481	-4.22085163
94	H	5.51854140	-1.00641827	-4.38091418
95	H	4.13526192	-0.16660962	-5.09400580
96	H	4.41849417	-0.09234361	-3.33705884
97	C	4.02786789	-2.83585319	-2.80334440
98	C	2.95816175	-2.52269562	-1.71455549
99	C	3.43754463	-1.40234964	-0.74893866
100	H	4.25666157	-1.78630653	-0.12948124
101	H	3.80260348	-0.52907809	-1.29792533
102	H	2.63095415	-1.07142220	-0.08935583
103	C	2.57846962	-3.79446208	-0.93299062
104	H	3.46176110	-4.17883553	-0.40906496
105	H	1.80227472	-3.59219741	-0.19307753
106	H	2.19800788	-4.56857451	-1.60650187

$[BiBr(CAAC)_2]^{+\bullet}$ (-13426.7)

1	C	-1.00875574	-1.64242176	0.37146712
2	N	-2.13668412	-0.99457040	0.57821910
3	H	4.99681480	-2.64828031	-2.43128594
4	C	-0.54171068	-2.33216438	1.65756967
5	C	-2.59155652	0.09900871	-0.28660315
6	C	-2.82537634	-1.36646495	1.92801372
7	C	-0.50277022	-3.87641699	1.59081576
8	C	0.86753057	-1.80270752	2.02820194
9	C	-1.58539785	-1.82820640	2.71273007
10	C	-3.53354378	-0.11550249	-1.31914977
11	C	-1.98034987	1.36333379	-0.06223777
12	C	-3.55476038	-0.17176021	2.54075281
13	C	-3.81103164	-2.52246090	1.67456534
14	H	-1.49402294	-4.30026660	1.41939871
15	H	0.14417410	-4.23015978	0.78763760
16	H	-0.11931936	-4.25031184	2.54772431
17	H	1.63531135	-2.20380076	1.36535306
18	H	0.91315736	-0.70948118	2.00005188
19	H	1.09992971	-2.12755076	3.04930421
20	H	-1.83557562	-2.61346281	3.43062341
21	H	-1.16772357	-0.98293717	3.27063266
22	C	-3.83545242	0.97717920	-2.15293245
23	C	-4.23136661	-1.45048231	-1.56698233
24	C	-2.32001590	2.41006179	-0.93012446
25	C	-0.97684754	1.62256729	1.06447335
26	H	-2.87335476	0.63767038	2.80956672

27	H	-4.32116731	0.21760064	1.86196427
28	H	-4.05615367	-0.50821534	3.45506728
29	H	-4.70509421	-2.18055935	1.15116043
30	H	-3.35308593	-3.33060213	1.09799184
31	H	-4.12317032	-2.92297324	2.64549831
32	C	-3.23276871	2.22038647	-1.96987448
33	H	-4.54821712	0.84302561	-2.96244490
34	C	-4.13255390	-1.91209649	-3.03881801
35	C	-5.71986246	-1.35709987	-1.14702809
36	H	-3.74331975	-2.21544251	-0.95581066
37	H	-1.85950009	3.38535079	-0.79372025
38	C	0.46570122	1.77560388	0.53307389
39	C	-1.36990979	2.85495330	1.91172329
40	H	-0.98103644	0.75916710	1.73219265
41	H	-3.47617334	3.04572879	-2.63623546
42	H	-4.54977038	-2.92053141	-3.13620348
43	H	-4.69038868	-1.24634934	-3.70746566
44	H	-3.09522748	-1.95258987	-3.38717463
45	H	-5.83370072	-0.97850692	-0.12528589
46	H	-6.19384486	-2.34440565	-1.20691948
47	H	-6.26249073	-0.67568829	-1.81403162
48	H	0.77125782	0.90584604	-0.05997754
49	H	0.55461124	2.66307629	-0.10184269
50	H	1.16506296	1.88407111	1.37084848
51	H	-2.40190798	2.78226104	2.27302160
52	H	-1.27936973	3.78224803	1.33418450
53	H	-0.70405628	2.93553606	2.77951716
54	Bi	-0.43871655	-1.48033843	-1.96654401
55	H	3.93383580	-3.94235461	-3.00290757
56	H	1.05208225	-3.77154003	-4.73089695
57	Br	-1.38669602	-4.37300305	-1.74639264
58	N	2.13599915	-1.71689695	-3.74431525
59	C	1.76632692	-1.98435419	-2.50191101
60	C	1.26648797	-1.08250464	-4.74148968
61	C	1.19171678	0.33789551	-4.79102217
62	C	0.41604882	0.91618199	-5.80895578
63	H	0.34793496	1.99943955	-5.86979506
64	C	-0.27309580	0.13550169	-6.73453911
65	H	-0.85999883	0.60881304	-7.51966075
66	C	-0.22713341	-1.25291127	-6.63627157
67	H	-0.79514007	-1.85557165	-7.34024229
68	C	0.52825800	-1.89773228	-5.64189654
69	C	0.44713656	-3.42326923	-5.57478617
70	C	-1.01115119	-3.87738849	-5.32621234
71	H	-1.04534355	-4.96718126	-5.20982091
72	H	-1.42960410	-3.44498886	-4.41400709
73	H	-1.65306110	-3.60446922	-6.17229070
74	C	0.96343406	-4.09471012	-6.87223671
75	H	0.29092404	-3.87650047	-7.71065644
76	H	1.96498988	-3.75781312	-7.15058372
77	H	0.98887709	-5.18335393	-6.73996238
78	C	1.84414668	1.28515452	-3.78175395
79	H	2.39516456	0.69133425	-3.04699004
80	C	0.76504274	2.08834579	-3.01841115
81	H	0.05430351	1.43203232	-2.50583336
82	H	1.23846618	2.72932757	-2.26723553
83	H	0.19403865	2.72929058	-3.69979184
84	C	2.82875708	2.27470649	-4.45078438
85	H	3.58735276	1.76541572	-5.05089243
86	H	2.29232095	2.97003764	-5.10736352
87	H	3.33914016	2.86725363	-3.68163171

88	C	3.62767393	-2.06128194	-4.04050426
89	C	3.75400329	-2.89297450	-5.31702998
90	H	3.21362138	-3.83807876	-5.23715963
91	H	3.39767816	-2.33938658	-6.19130637
92	H	4.81488624	-3.12004391	-5.47217203
93	C	4.44933136	-0.77438028	-4.19257871
94	H	5.49796181	-1.05519937	-4.33979860
95	H	4.12730217	-0.21091351	-5.07174056
96	H	4.39164830	-0.12998510	-3.31274341
97	C	3.98307267	-2.87260850	-2.77463386
98	C	2.92016142	-2.53099382	-1.68771301
99	C	3.41750203	-1.41162603	-0.73086530
100	H	4.22349145	-1.80597022	-0.10084554
101	H	3.80526302	-0.55240571	-1.28611439
102	H	2.61335219	-1.05604396	-0.08135978
103	C	2.51486283	-3.78847834	-0.89695586
104	H	3.39023766	-4.19326115	-0.37521455
105	H	1.75020066	-3.56001962	-0.15287186
106	H	2.11188750	-4.55848980	-1.56288664

[Cl(CAAC)$_2$]$^{\bullet}$ (-13451.4)

1	C	-0.90872076	-1.12249587	0.48271316
2	N	-2.24803703	-0.77404391	0.63021027
3	H	4.42746818	0.77011452	-4.75434358
4	C	-0.60733855	-2.42450673	1.20329149
5	C	-2.73928450	0.52075004	0.23482891
6	C	-2.93541682	-1.61808339	1.68340891
7	C	-0.69559822	-3.66226060	0.27008373
8	C	0.79212046	-2.39311388	1.85455846
9	C	-1.73906304	-2.42147037	2.27170539
10	C	-3.65277665	0.61240817	-0.84191864
11	C	-2.30114590	1.68801495	0.91315985
12	C	-3.61416379	-0.75357813	2.75906223
13	C	-3.99970018	-2.54356279	1.05595279
14	H	-1.65085380	-3.70181826	-0.26071785
15	H	0.10067680	-3.62732205	-0.48029126
16	H	-0.58421359	-4.58375148	0.85534935
17	H	1.56730815	-2.30609198	1.08437304
18	H	0.88500162	-1.53723178	2.53215166
19	H	0.97581482	-3.31383013	2.42374430
20	H	-2.03703596	-3.43502702	2.55742815
21	H	-1.37848262	-1.91242084	3.17269297
22	C	-4.20836560	1.86474837	-1.14772385
23	C	-3.96534605	-0.57500101	-1.74630002
24	C	-2.87160486	2.91849078	0.55909094
25	C	-1.19098164	1.65966394	1.96079504
26	H	-2.89542992	-0.10480716	3.26406077
27	H	-4.40353879	-0.12990994	2.32650330
28	H	-4.06995314	-1.40885668	3.51013403
29	H	-4.83210101	-1.96075933	0.65358435
30	H	-3.58400894	-3.15416024	0.25120345
31	H	-4.39814647	-3.21463868	1.82651501
32	C	-3.83893990	3.00762923	-0.44274632
33	H	-4.91553462	1.94821392	-1.96950147
34	C	-3.33459418	-0.35933419	-3.14132695
35	C	-5.47942660	-0.84868819	-1.87230417
36	H	-3.48995234	-1.45436068	-1.30668899
37	H	-2.55230152	3.82224906	1.07193457
38	C	0.13242583	2.16762707	1.33871912
39	C	-1.53844123	2.45353233	3.23811154
40	H	-1.02281886	0.61852047	2.24479642

41	H	-4.27664647	3.97169593	-0.69453954
42	H	-3.52833607	-1.22670617	-3.78294653
43	H	-3.75305686	0.52500688	-3.63335378
44	H	-2.25265486	-0.22504792	-3.06994072
45	H	-5.95369368	-0.96938691	-0.89182854
46	H	-5.65233107	-1.76450259	-2.45101806
47	H	-5.98955844	-0.02709930	-2.39009379
48	H	0.39666812	1.57673278	0.45732416
49	H	0.03918941	3.21796658	1.03517240
50	H	0.94835349	2.09229958	2.06888293
51	H	-2.49055888	2.12354962	3.66907826
52	H	-1.61458681	3.52915825	3.04000971
53	H	-0.75361093	2.31153279	3.99121500
54	Cl	-0.15469675	-0.81684542	-1.11553324
55	H	0.77121993	-1.50619924	-6.08287605
56	N	1.18248420	0.52857726	-4.84207568
57	C	1.36922014	-0.08450223	-3.70337636
58	C	-0.15446121	0.84816677	-5.31444256
59	C	-0.75236123	2.06925829	-4.93695452
60	C	-2.02295608	2.36440125	-5.45581166
61	H	-2.50912115	3.29580023	-5.17770036
62	C	-2.68684864	1.46824954	-6.29237989
63	H	-3.67122670	1.71596832	-6.68438413
64	C	-2.10973870	0.23193077	-6.58560245
65	H	-2.66239995	-0.48630023	-7.18515603
66	C	-0.84129231	-0.10808452	-6.09469139
67	C	-0.30114556	-1.52344982	-6.28377552
68	C	-0.92740312	-2.45790296	-5.22011213
69	H	-0.50935331	-3.46868177	-5.31147701
70	H	-0.71944243	-2.07950501	-4.21399830
71	H	-2.01448892	-2.52054743	-5.35143550
72	C	-0.49819995	-2.07404439	-7.70931091
73	H	-1.55882628	-2.21061780	-7.95066624
74	H	-0.06398344	-1.40226664	-8.45889213
75	H	-0.01126549	-3.05234419	-7.80239008
76	C	-0.13360043	2.99011920	-3.88893621
77	H	0.90613570	2.69420515	-3.74929832
78	C	-0.84167932	2.77766811	-2.53129712
79	H	-0.76487279	1.73637864	-2.20922530
80	H	-0.38331106	3.40799379	-1.76002279
81	H	-1.90311474	3.03671121	-2.59495887
82	C	-0.14811722	4.47678287	-4.29919977
83	H	0.33971545	4.63266261	-5.26831582
84	H	-1.17063902	4.86627762	-4.36952079
85	H	0.38205142	5.07638302	-3.54923735
86	C	2.44573710	0.92633591	-5.68226929
87	C	2.31986054	0.45738730	-7.13801786
88	H	2.24606510	-0.63035346	-7.20957327
89	H	1.44573602	0.90206425	-7.62557943
90	H	3.21147638	0.77173057	-7.69287663
91	C	2.64351481	2.45084549	-5.66173223
92	H	3.52893127	2.70360534	-6.25619220
93	H	1.78460287	2.96621444	-6.10256889
94	H	2.79753690	2.82970311	-4.64837281
95	C	3.52571912	0.16321322	-4.88345390
96	C	2.87359564	-0.22824888	-3.51211424
97	C	3.28333055	0.73614332	-2.36881484
98	H	4.35807718	0.64161408	-2.16740649
99	H	3.07461790	1.77980858	-2.62834909
100	H	2.72631137	0.49800728	-1.45688557
101	C	3.21197158	-1.67671398	-3.10453438

102	H	4.29567229	-1.79471417	-2.97150131
103	H	2.70830547	-1.93351981	-2.16654484
104	H	2.88176484	-2.38565396	-3.87341224
105	H	3.81277847	-0.74383547	-5.42698643

$[Br(CAAC)_2]^{\bullet}$ (-13443.3)

1	C	-0.95294520	-1.08941508	0.50540656
2	N	-2.28964130	-0.75148951	0.62238049
3	H	4.47795757	0.66216265	-4.95004231
4	C	-0.63954175	-2.35017418	1.29426032
5	C	-2.76108860	0.55367450	0.23430479
6	C	-3.01496245	-1.62144548	1.63267368
7	C	-0.61721606	-3.63446545	0.42380592
8	C	0.72163238	-2.22223152	2.01483136
9	C	-1.82387253	-2.35862760	2.30759227
10	C	-3.65103478	0.67628911	-0.85825805
11	C	-2.31572925	1.70390536	0.93726694
12	C	-3.80716758	-0.77700624	2.64303447
13	C	-3.98343798	-2.60770537	0.94439572
14	H	-1.53716305	-3.74895970	-0.15570758
15	H	0.21760715	-3.59675236	-0.28291493
16	H	-0.49602313	-4.51852083	1.06289184
17	H	1.53088409	-2.13357499	1.28082343
18	H	0.74066962	-1.33240200	2.65350278
19	H	0.91608431	-3.10578604	2.63726048
20	H	-2.09690213	-3.37350344	2.61308771
21	H	-1.52914834	-1.80657275	3.20738444
22	C	-4.16010092	1.94625305	-1.17263989
23	C	-4.00449302	-0.50177575	-1.75881954
24	C	-2.84015180	2.95206576	0.57517587
25	C	-1.25298731	1.63740135	2.03115388
26	H	-3.15785958	-0.08505874	3.18408775
27	H	-4.59366629	-0.19963989	2.14571666
28	H	-4.28215310	-1.44298469	3.37246941
29	H	-4.83133875	-2.07895326	0.50330994
30	H	-3.48688347	-3.18091719	0.15821493
31	H	-4.37473694	-3.31184678	1.68869618
32	C	-3.77294538	3.07541756	-0.45565199
33	H	-4.84635432	2.05294482	-2.00936868
34	C	-3.42943277	-0.28869108	-3.17666246
35	C	-5.52659402	-0.75515204	-1.82573499
36	H	-3.52175277	-1.38779621	-1.34116737
37	H	-2.51247957	3.84281078	1.10535725
38	C	0.10839969	2.12269520	1.47835164
39	C	-1.64249857	2.42092877	3.30312343
40	H	-1.12288196	0.58960748	2.30953832
41	H	-4.17306041	4.05348510	-0.71581521
42	H	-3.63968301	-1.16077245	-3.80653793
43	H	-3.87291770	0.58918299	-3.65761875
44	H	-2.34686789	-0.14544026	-3.14551941
45	H	-5.96457824	-0.86828636	-0.82765705
46	H	-5.73653758	-1.66757308	-2.39765891
47	H	-6.04252382	0.07618716	-2.32195735
48	H	0.39819854	1.53513712	0.60264580
49	H	0.05275957	3.17874991	1.18537787
50	H	0.88792563	2.01920055	2.24403133
51	H	-2.61904732	2.10320498	3.68637523
52	H	-1.68984625	3.50041674	3.11756241
53	H	-0.89516950	2.25323004	4.08842605
54	Br	-0.07452400	-0.80136756	-1.26480272
55	H	0.72233897	-1.56544570	-6.04139231

56	N	1.22603192	0.48391941	-4.86449025
57	C	1.46766494	-0.12880334	-3.73778571
58	C	-0.12550459	0.82227717	-5.28058865
59	C	-0.68639274	2.05854371	-4.89633852
60	C	-1.96171862	2.37903056	-5.38854774
61	H	-2.41964896	3.32311718	-5.10547978
62	C	-2.66422271	1.49456903	-6.20496242
63	H	-3.65077852	1.76320170	-6.57699207
64	C	-2.12398455	0.24245419	-6.50043289
65	H	-2.70707891	-0.46706335	-7.08114423
66	C	-0.85351796	-0.12447518	-6.03540349
67	C	-0.35258300	-1.55355554	-6.22988934
68	C	-0.99356358	-2.47271552	-5.16150155
69	H	-0.59970670	-3.49286271	-5.25549285
70	H	-0.77354133	-2.10151031	-4.15546014
71	H	-2.08243419	-2.51039031	-5.28654842
72	C	-0.58163491	-2.09815028	-7.65325282
73	H	-1.64854011	-2.20704833	-7.88038245
74	H	-0.14039600	-1.43766592	-8.40856667
75	H	-0.12140328	-3.08864100	-7.75263492
76	C	-0.02385369	2.98072948	-3.87641283
77	H	1.00311355	2.64364828	-3.73073158
78	C	-0.73713110	2.84456258	-2.51130392
79	H	-0.71138391	1.81186297	-2.15513670
80	H	-0.24500099	3.47594927	-1.76163938
81	H	-1.78496732	3.15346737	-2.58055720
82	C	0.02135451	4.45467078	-4.33085693
83	H	0.50600970	4.56336649	-5.30754645
84	H	-0.98543070	4.88267199	-4.40458347
85	H	0.58190948	5.05323548	-3.60252419
86	C	2.45285580	0.85629722	-5.76983299
87	C	2.24325650	0.38870119	-7.21586079
88	H	2.14034017	-0.69699067	-7.28065406
89	H	1.35746913	0.85385777	-7.66103814
90	H	3.11418356	0.68097945	-7.81381295
91	C	2.67747706	2.37642173	-5.75932796
92	H	3.54552784	2.61236397	-6.38528577
93	H	1.81263890	2.90489984	-6.17130717
94	H	2.87291828	2.75445154	-4.75286773
95	C	3.55873665	0.07335548	-5.02831378
96	C	2.97507096	-0.30199092	-3.62282814
97	C	3.46542682	0.65673076	-2.50678575
98	H	4.54750088	0.54153074	-2.36574861
99	H	3.26275245	1.70364228	-2.75770980
100	H	2.95598021	0.43097804	-1.56444539
101	C	3.30667929	-1.75620577	-3.22985795
102	H	4.39357015	-1.89408820	-3.15728502
103	H	2.85185844	-2.00109935	-2.26420964
104	H	2.92082473	-2.46038885	-3.97671374
105	H	3.79731149	-0.84054620	-5.58349556

Table S17. Computed EPR data for compound **8** at level B3LYP/6-311++G** & SDD(Bi) with Gaussian16 on the geometries optimized at the ZORA-BLYP-D3(BJ)/TZ2P in THF.

Atom numbering of the couplings listed below for compound **8**.

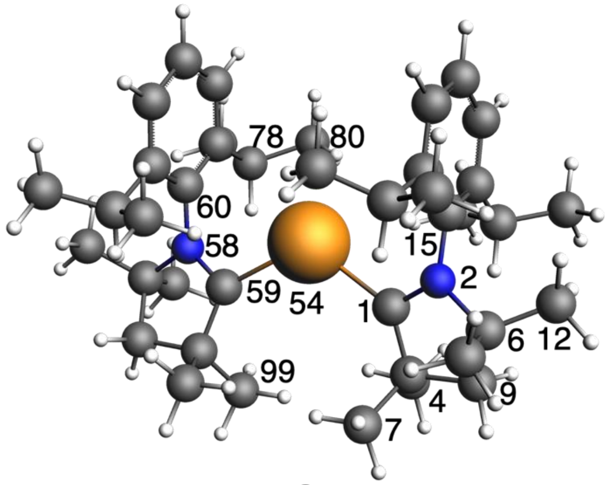

8

Compound 8

Atom		Isotropic Fermi Contact Couplings a.u.	MegaHertz	Gauss	10(-4) cm-1
1	C(13)	0.06388	71.81404	25.62504	23.95459
**2**	**N(14)**	**0.01238**	**3.99957**	**1.42714**	**1.33411**
3	H(1)	-0.00021	-0.94016	-0.33547	-0.31360
4	C(13)	-0.01043	-11.72190	-4.18267	-3.91001
5	C(13)	0.00181	2.03239	0.72521	0.67793
6	C(13)	-0.00109	-1.22407	-0.43678	-0.40831
7	C(13)	0.02560	28.78004	10.26943	9.59999
8	C(13)	0.01759	19.76911	7.05411	6.59427
9	C(13)	0.00390	4.38159	1.56346	1.46154
10	C(13)	0.00585	6.57198	2.34505	2.19218
11	C(13)	0.00468	5.26287	1.87792	1.75550
12	C(13)	0.00017	0.19450	0.06940	0.06488
13	C(13)	0.00465	5.22555	1.86461	1.74306
14	H(1)	-0.00016	-0.70623	-0.25200	-0.23557
15	H(1)	-0.00006	-0.28000	-0.09991	-0.09340
16	H(1)	0.00152	6.77410	2.41717	2.25960
17	H(1)	-0.00013	-0.56424	-0.20134	-0.18821
18	H(1)	0.00001	0.03040	0.01085	0.01014
19	H(1)	0.00181	8.08371	2.88447	2.69643
20	H(1)	-0.00021	-0.93129	-0.33231	-0.31065
21	H(1)	-0.00014	-0.60853	-0.21714	-0.20298
22	C(13)	-0.00047	-0.52284	-0.18656	-0.17440
23	C(13)	-0.00102	-1.15224	-0.41115	-0.38435
24	C(13)	0.00180	2.01933	0.72055	0.67358
25	C(13)	-0.00057	-0.63851	-0.22784	-0.21298
26	H(1)	-0.00003	-0.13327	-0.04755	-0.04445
27	H(1)	-0.00004	-0.17651	-0.06298	-0.05888
28	H(1)	-0.00012	-0.55665	-0.19863	-0.18568
29	H(1)	0.00002	0.08331	0.02973	0.02779
30	H(1)	-0.00015	-0.66230	-0.23632	-0.22092
31	H(1)	0.00058	2.61067	0.93155	0.87083
32	C(13)	0.00038	0.43108	0.15382	0.14379

33	H(1)	0.00007	0.31298	0.11168	0.10440
34	C(13)	-0.00148	-1.66200	-0.59304	-0.55438
35	C(13)	0.00074	0.82844	0.29561	0.27634
36	H(1)	-0.00001	-0.02703	-0.00965	-0.00902
37	H(1)	0.00012	0.54971	0.19615	0.18336
38	C(13)	-0.00112	-1.25953	-0.44943	-0.42013
39	C(13)	-0.00019	-0.21352	-0.07619	-0.07122
40	H(1)	0.00013	0.57283	0.20440	0.19108
41	H(1)	-0.00009	-0.41924	-0.14960	-0.13984
42	H(1)	0.00000	-0.00013	-0.00005	-0.00004
43	H(1)	0.00001	0.04911	0.01752	0.01638
44	H(1)	0.00001	0.04561	0.01627	0.01521
45	H(1)	-0.00002	-0.09301	-0.03319	-0.03102
46	H(1)	0.00005	0.22462	0.08015	0.07492
47	H(1)	-0.00005	-0.21654	-0.07727	-0.07223
48	H(1)	0.00013	0.57102	0.20376	0.19047
49	H(1)	0.00017	0.76818	0.27411	0.25624
50	H(1)	-0.00003	-0.13630	-0.04863	-0.04546
51	H(1)	-0.00002	-0.07826	-0.02793	-0.02611
52	H(1)	0.00001	0.04894	0.01746	0.01633
53	H(1)	0.00000	0.01463	0.00522	0.00488
**54**	**Bi(209**	**0.00000**	**-0.00064**	**-0.00023**	**-0.00021**
55	H(1)	-0.00014	-0.61236	-0.21851	-0.20426
56	H(1)	0.00013	0.58695	0.20944	0.19578
57	H(1)	0.00001	0.05487	0.01958	0.01830
**58**	**N(14)**	**0.01245**	**4.02393**	**1.43584**	**1.34224**
59	C(13)	0.06399	71.93284	25.66742	23.99421
60	C(13)	0.00179	2.01096	0.71756	0.67078
61	C(13)	0.00591	6.64435	2.37087	2.21632
62	C(13)	-0.00047	-0.53211	-0.18987	-0.17749
63	H(1)	0.00007	0.31980	0.11411	0.10667
64	C(13)	0.00039	0.44110	0.15739	0.14713
65	H(1)	-0.00010	-0.42944	-0.15324	-0.14325
66	C(13)	0.00181	2.03446	0.72595	0.67862
67	H(1)	0.00012	0.55670	0.19865	0.18570
68	C(13)	0.00475	5.33525	1.90375	1.77965
69	C(13)	-0.00057	-0.63976	-0.22828	-0.21340
70	C(13)	-0.00116	-1.30631	-0.46612	-0.43574
71	H(1)	-0.00003	-0.13371	-0.04771	-0.04460
72	H(1)	0.00013	0.56828	0.20278	0.18956
73	H(1)	0.00017	0.76112	0.27158	0.25388
74	C(13)	-0.00019	-0.20901	-0.07458	-0.06972
75	H(1)	0.00001	0.05521	0.01970	0.01842
76	H(1)	-0.00002	-0.07923	-0.02827	-0.02643
77	H(1)	0.00000	0.01583	0.00565	0.00528
78	C(13)	-0.00105	-1.17899	-0.42069	-0.39327
79	H(1)	-0.00001	-0.03061	-0.01092	-0.01021
80	C(13)	-0.00139	-1.56293	-0.55769	-0.52134
81	H(1)	0.00000	0.02113	0.00754	0.00705
82	H(1)	0.00000	-0.00496	-0.00177	-0.00165
83	H(1)	0.00001	0.04143	0.01478	0.01382
84	C(13)	0.00076	0.84917	0.30301	0.28325
85	H(1)	-0.00002	-0.08900	-0.03176	-0.02969
86	H(1)	-0.00005	-0.21924	-0.07823	-0.07313
87	H(1)	0.00005	0.22778	0.08128	0.07598
88	C(13)	-0.00113	-1.27555	-0.45515	-0.42548
89	C(13)	0.00018	0.20094	0.07170	0.06703
90	H(1)	-0.00003	-0.13417	-0.04788	-0.04475
91	H(1)	-0.00004	-0.17927	-0.06397	-0.05980
92	H(1)	-0.00013	-0.56105	-0.20020	-0.18715
93	C(13)	0.00467	5.24855	1.87281	1.75073

94	H(1)	0.00058	2.61167	0.93191	0.87116
95	H(1)	0.00002	0.08649	0.03086	0.02885
96	H(1)	-0.00015	-0.67068	-0.23931	-0.22371
97	C(13)	0.00389	4.37386	1.56070	1.45896
98	C(13)	-0.01051	-11.81821	-4.21703	-3.94213
99	C(13)	0.02564	28.82497	10.28547	9.61498
100	H(1)	0.00151	6.74295	2.40605	2.24920
101	H(1)	-0.00016	-0.72356	-0.25819	-0.24136
102	H(1)	-0.00006	-0.28585	-0.10200	-0.09535
103	C(13)	0.01790	20.12637	7.18159	6.71343
104	H(1)	0.00182	8.12776	2.90019	2.71113
105	H(1)	-0.00014	-0.61613	-0.21985	-0.20552

Table S18. Computed EPR data for compound **8** at ZORA-BLYP-D3(BJ)/TZ2P in THF.

Compound **8**

==== g-tensor

	X	Y	Z
X	1.998738	0.006101	-0.015884
Y	0.006101	2.012943	0.022995
Z	-0.015884	0.022995	1.994790

===== principal axes g-tensor

	11	22	33
X	0.497686	-0.853622	0.153747
Y	-0.460401	-0.410218	-0.787243
Z	0.735078	0.321015	-0.597168

	g11	g22	g33
Principal g-values	1.969633	2.007643	2.029194
g-g_e:	-0.032687	0.005324	0.026875

Isotropic g-value: 2.002157, isotropic g-g_e: -0.000163

Effective spin S = 0.5 used (relevant for g-tensor and A-tensor)
